# Thirty-five new species of the spider genus *Pimoa* (Araneae, Pimoidae) from Pan-Himalaya

**DOI:** 10.3897/zookeys.1029.64080

**Published:** 2021-04-08

**Authors:** Hao Xu, Xiaoqing Zhang, Zhiyuan Yao, Abid Ali, Shuqiang Li

**Affiliations:** 1 College of Life Science, Shenyang Normal University, Shenyang 110034, Liaoning, China Shenyang Normal University Shenyang China; 2 Institute of Zoology, Chinese Academy of Sciences, Beijing 100101, China Institute of Zoology, Chinese Academy of Sciences Beijing China; 3 Department of Entomology, Faculty of Agriculture, University of Agriculture, Faisalabad-38040, Punjab, Pakistan University of Agriculture Faisalabad Pakistan

**Keywords:** Asia, description, diagnosis, DNA barcodes, taxonomy

## Abstract

Thirty-five new species of the *Pimoa* Chamberlin & Ivie, 1943 are described from Pan-Himalaya: *P. anning* Zhang & Li, **sp. nov.** (♂♀), *P. bomi* Zhang & Li, **sp. nov.** (♂♀), *P. cawarong* Zhang & Li, **sp. nov.** (♀), *P. daman* Zhang & Li, **sp. nov.** (♀), *P. danba* Zhang & Li, **sp. nov.** (♀), *P. deqen* Zhang & Li, s**p. nov.** (♀), *P. dongjiu* Zhang & Li, **sp. nov.** (♂♀), *P. guiqing* Zhang & Li, **sp. nov.** (♀), *P. gyaca* Zhang & Li, **sp. nov.** (♀), *P. gyara* Zhang & Li, **sp. nov.** (♂♀), *P. gyirong* Zhang & Li, **sp. nov.** (♂♀), *P. heishui* Zhang & Li, **sp. nov.** (♂♀), *P. jinchuan* Zhang & Li, **sp. nov.** (♂♀), *P. khaptad* Zhang & Li, **sp. nov.** (♀), *P. koshi* Zhang & Li, **sp. nov.** (♀), *P. lhatog* Zhang & Li, **sp. nov.** (♀), *P. mechi* Zhang & Li, **sp. nov.** (♂♀), *P. miandam* Zhang & Li, **sp. nov.** (♂♀), *P. miero* Zhang & Li, **sp. nov.** (♂♀), *P. mude* Zhang & Li, **sp. nov.** (♀), *P. muli* Zhang & Li, **sp. nov.** (♂♀), *P. naran* Zhang & Li, **sp. nov.** (♀), *P. ninglang* Zhang & Li, **sp. nov.** (♀), *P. nyalam* Zhang & Li, **sp. nov.** (♂♀), *P. phaplu* Zhang & Li, **sp. nov.** (♂♀), *P. putou* Zhang & Li, **sp. nov.** (♀), *P. rara* Zhang & Li, **sp. nov.** (♀), *P. sangri* Zhang & Li, **sp. nov.** (♂♀), *P. shigatse* Zhang & Li, **sp. nov.** (♀), *P. tengchong* Zhang & Li, **sp. nov.** (♂♀), *P. xiahe* Zhang & Li, **sp. nov.** (♂♀), *P. yejiei* Zhang & Li, **sp. nov.** (♀), *P. yele* Zhang & Li, **sp. nov.** (♂♀), *P. zayu* Zhang & Li, **sp. nov.** (♂♀), *P. zhigangi* Zhang & Li, **sp. nov.** (♀). The DNA barcodes of the thirty-five new species are provided.

## Introduction

The spider family Pimoidae Wunderlich, 1986 was considered as a subfamily of Linyphiidae Blackwall, 1859 until Hormiga (1993) elevated it to family level. It is a small family with 53 named species in four genera: *Nanoa* Hormiga, Buckle & Scharff, 2005, *Pimoa* Chamberlin & Ivie, 1943, *Putaoa* Hormiga & Tu, 2008, and *Weintrauboa* Hormiga, 2003 (Li 2020). *Pimoa* is the most species-rich genus in Pimoidae, with the type species *Pimoa hespera* (Gertsch & Ivie, 1936) from the USA, and contains 41 described species before the current study (WSC 2021).

The genus *Pimoa* has a disjunct distribution: It’s known from the west coast of the USA, from Washington to California in the Nearctic, the Alps, the Cantabrian Mountains of northern Spain, and East Asia (Himalaya to Beijing) in the Palaearctic (Mammola et al. 2016; Zhang and Li 2020; WSC 2021). More than half of all pimoids are known from Asia. Seventeen species have thus far been described from China, most distributed in Tibet and Sichuan Province, and others in Beijing, Hunan, and Yunnan Provinces (Hormiga 1994a; Griswold et al. 1999; Xu and Li 2007; Xu and Li 2009; Yuan et al. 2019; Zhang and Li 2019; Zhang et al. 2020). Pimoids mainly occur in wet and cold environments, such as in the crevices of mossy rocks or tree hollows.

After examining specimens collected from Pan-Himalaya, part of an ongoing project about the phylogeny of Pimoidae, we recognized 35 new species, of which 26 are from China, seven are from Nepal, and two are from Pakistan. DNA barcodes were also obtained for the new species.

## Materials and methods

Specimens were examined with a LEICA M205C stereomicroscope. Images were captured with an Olympus C7070 wide zoom digital camera (7.1 megapixels) mounted on an Olympus SZX12 dissecting microscope, subsequently assembled using Helicon Focus 3.10.3 image stacking software (Khmelik et al. 2006). Epigynes and male palps were examined after dissection from the spiders’ bodies. The left palps were illustrated unless otherwise noted. Epigynes were removed and treated in a warmed 10% potassium hydroxide (KOH) solution.

All measurements were obtained using a LEICA M205C stereomicroscope and are given in millimeters. We measured the length of the legs and body using a ruler in the eyepiece. Eye sizes were measured as the maximum diameter from either dorsal or frontal views. Leg measurements are shown as total length (femur, patella + tibia, metatarsus, tarsus). The terminology used in the text and the figure legends follows Hormiga (1994a). The distribution map was generated using ArcView GIS 3.2 software (ESRI 2002).

Abbreviations used in this paper and in the figure legends:

**ALE** anterior lateral eye;

**AME** anterior median eye;

**AME-ALE** distance between AME and ALE;

**AME-AME** distance between AMEs;

**AS** alveolar sclerite;

**C** conductor;

**CDP** cymbial denticulate process;

**CO** copulatory opening;

**DP** dorsal plate of the epigyne;

**E** embolus;

**FD** fertilization duct;

**MA** median apophysis;

**P** paracymbium;

**PCS** pimoid cymbial sclerite;

**PEP** pimoid embolic process;

**PLE** posterior lateral eye;

**PME** posterior median eye;

**PME-PLE** distance between PME and PLE;

**PME-PME** distance between PMEs;

**S** spermatheca;

**T** tegulum;

**VP** ventral plate of epigyne.

## Taxonomy

### Family Pimoidae Wunderlich, 1986

#### Genus Pimoa

Taxon classificationAnimaliaAraneaePimoidae

Chamberlin & Ivie, 1943

92FCF6B6-1AD6-5026-9B6D-F052CB713889

Pimoa : Chamberlin and Ivie 1943: 9; Hormiga 1994a: 4; Hormiga and Lew 2014: 1; Mammola et al. 2016: 1.

##### Type species.

*Labulla hespera* Gertsch & Ivie, 1936, from California, USA.

##### Diagnosis.

**Figure 1.**
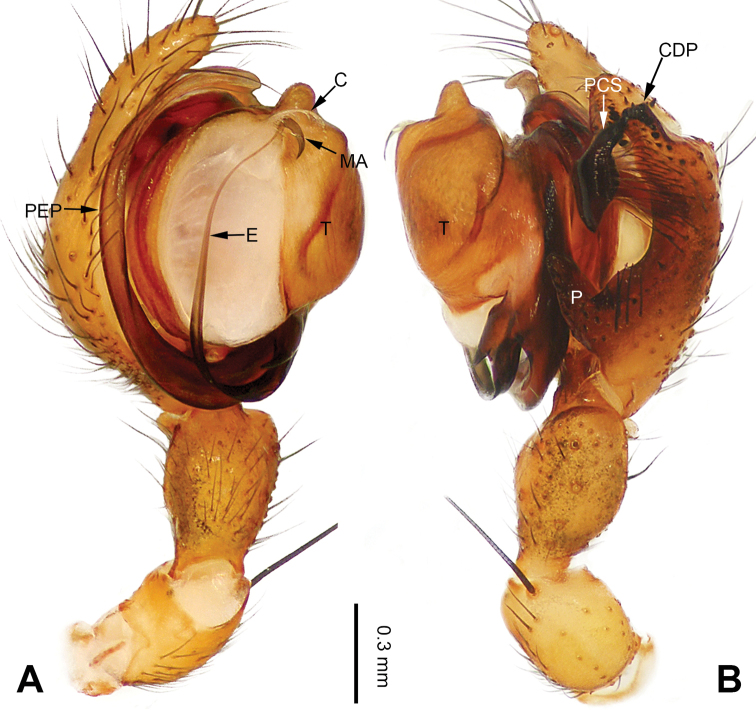
Left palp of *Pimoa anning* sp. nov., holotype **A** prolateral view **B** retrolateral view. Abbreviations: C = conductor; CDP = cymbial denticulate process; E = embolus; MA = median apophysis; P = paracymbium; PCS = pimoid cymbial sclerite; PEP = pimoid embolic process; T = tegulum. Scale bar: equal for **A, B**.

**Figure 2.**
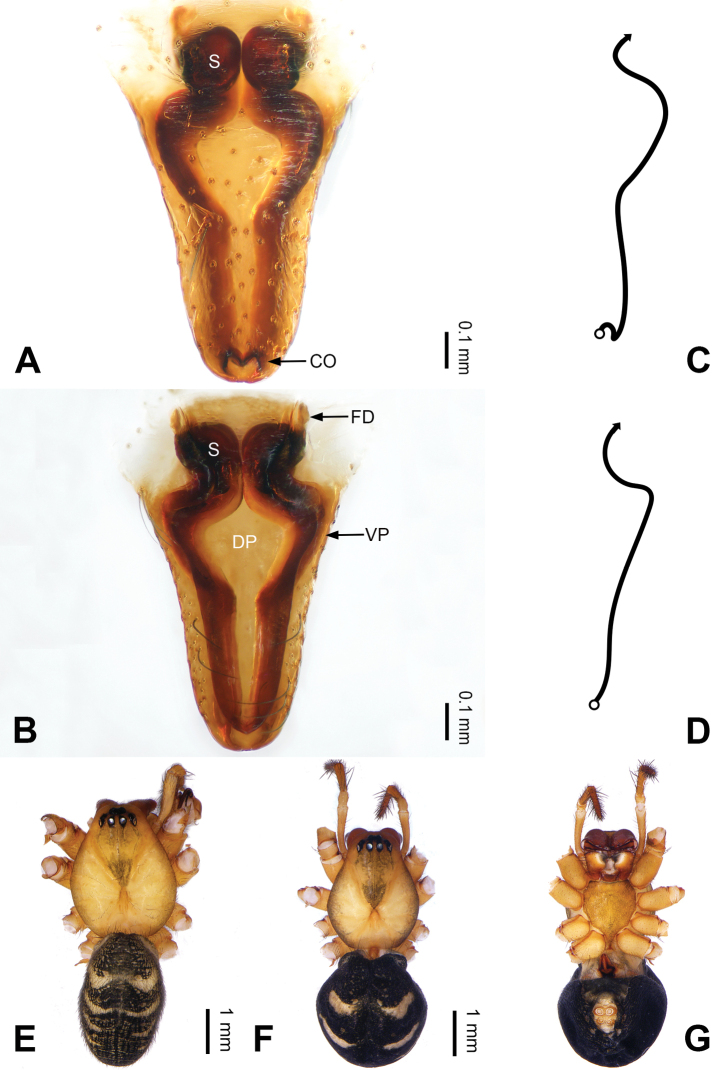
Epigyne and habitus of *Pimoa anning* sp. nov., female paratype and male holotype **A** epigyne, ventral view **B** vulva, dorsal view **C** schematic course of internal duct system, ventral view **D** schematic course of internal duct system, dorsal view **E** male habitus, dorsal view **F** female habitus, dorsal view **G** female habitus, ventral view. Abbreviations: CO = copulatory opening; DP = dorsal plate of the epigyne; FD = fertilization duct; S = spermatheca; VP = ventral plate of epigyne. Scale bars: equal for **F, G**.

*Pimoa* is larger in size than other genera occurring in the region, 4.0–12.0 mm. The chelicerae of most species of *Pimoa* (including the type species) have three promarginal and three retromarginal teeth. Males of *Pimoa* can be distinguished from *Nanoa* by the small median apophysis and the elongate cymbial denticulate process with many cuspules (vs. the large median apophysis and short cymbial process only with one strong cuspule). (Fig. 1A, B; Hormiga 2005: figs 1, 2), from *Putaoa* by the slender embolus and the absence of distinctly large macrosetae on the palpal tibia (vs. the thick embolus and many robust macrosetae on the pedipalpal tibia) (Fig. 1A, B; Hormiga and Tu 2008: figs 3, 5, 6), and from *Weintrauboa* by the clockwise pimoid embolic process and the absence of an embolic flap (vs. counterclockwise pimoid embolic process and a membranous embolic flap arising from the embolus) (Fig. 1A, B; Hormiga 2003: figs 1, 2). Females of *Pimoa* can be distinguished from *Nanoa* the absence of a ventral scape-like septum (vs. epigynum with a distinct septum) (Fig. 2A; Hormiga 2005: figs 3, 5), from *Putaoa* by the protruding epigynum and the absence of lateral openings on the epigyne (vs. relatively flat epigynum with two lateral openings) (Fig. 2A–D; Hormiga and Tu 2008: figs 2, 4, 8), and from *Weintrauboa* by the epigyne with a distinct groove at the margin of the dorsal plate (vs. dorsal plate absent) (Fig. 2A–D; Hormiga 2003: figs 2, 3).

##### Composition.

Seventy-six valid species of *Pimoa* are currently known from Asia (58), Europe (4) and North America (14) (WSC 2021).

**Table 1.** Voucher specimen information.

Species	GenBank accession number	Sequence length	Collection locality
*Pimoa anning* sp. nov.	MW727904	621 bp	Jinchuan County, Sichuan, China
*Pimoa bomi* sp. nov.	MW727915	621 bp	Bomi County, Nyingchi, Tibet, China
*Pimoa cawarong* sp. nov.	MW727894	621 bp	Zayu County, Nyingchi, Tibet, China
*Pimoa daman* sp. nov.	MW727922	621 bp	Daman, Narayani District, Nepal
*Pimoa danba* sp. nov.	MW727903	621 bp	Danba County, Sichuan, China
*Pimoa deqen* sp. nov.	MW727899	621 bp	Diqing Tibetan Autonomous Prefecture, Yunnan, China
*Pimoa dongjiu* sp. nov.	MW727897	621 bp	Bayi District, Nyingchi, Tibet, China
*Pimoa guiqing* sp. nov.	MW727927	621 bp	Tianshui, Gansu, China
*Pimoa gyaca* sp. nov.	MW727920	621 bp	Gyaca County, Lhoka, Tibet, China
*Pimoa gyara* sp. nov.	MW727916	621 bp	Gongbo’gyamda County, Nyingchi, Tibet, China
*Pimoa gyirong* sp. nov.	MW727913	621 bp	Gyirong County, Shigatse, Tibet, China
*Pimoa heishui* sp. nov.	MW727923	621 bp	Heishui County, Sichuan, China
*Pimoa jinchuan* sp. nov.	MW727901	621 bp	Jinchuan County, Sichuan, China
*Pimoa khaptad* sp. nov.	MW727930	621 bp	Khaptad National Park, Karnali District, Nepal
*Pimoa koshi* sp. nov.	MW727918	621 bp	Tamaphok, Koshi District, Nepal
*Pimoa lhatog* sp. nov.	MW727925	621 bp	Nang County, Nyingchi, Tibet, China
*Pimoa mechi* sp. nov.	MW727919	621 bp	Taplejung, Mechi District, Nepal
*Pimoa miandam* sp. nov.	MW727896	621 bp	Swat, Khyber Pakhtunkhwa, Pakistan
*Pimoa miero* sp. nov.	MW727902	621 bp	Li County, Sichuan, China
*Pimoa mude* sp. nov.	MW727929	621 bp	Mude, Baghmati District, Nepal
*Pimoa muli* sp. nov.	MW727924	621 bp	Muli County, Sichuan, China
*Pimoa naran* sp. nov.	MW727898	621 bp	Naran, Khyber Pakhtunkhwa, Pakistan
*Pimoa ninglang* sp. nov.	MW727893	621 bp	Ninglang Yi Autonomous County, Lijiang, Yunnan, China
*Pimoa nyalam* sp. nov.	MW727912	621 bp	Nyalam County, Shigatse, Tibet, China
*Pimoa phaplu* sp. nov.	MW727917	621 bp	Phaplu Airport, Sagarmatha District, Nepal
*Pimoa putou* sp. nov.	MW727900	621 bp	Li County, Sichuan, China
*Pimoa rara* sp. nov.	MW727907	621 bp	Rara National Park, Karnali District, Nepal
*Pimoa sangri* sp. nov.	MW727911	621 bp	Lhoka, Tibet, China
*Pimoa shigatse* sp. nov.	MW727921	621 bp	Shigatse, Tibet, China
*Pimoa tengchong* sp. nov.	MW727906	621 bp	Xincheng District, Tengchong, Yunnan, China
*Pimoa xiahe* sp. nov.	MW727910	621 bp	Xiahe County, Gannan Tibetan Autonomous Prefecture, Gansu, China
*Pimoa yejiei* sp. nov.	MW727928	621 bp	Mei County, Paoki, Shaanxi, China
*Pimoa yele* sp. nov.	MW727905	621 bp	Mianning County, Liangshan, Sichuan, China
*Pimoa zayu* sp. nov.	MW727895	621 bp	Zayu County, Nyingchi, Tibet, China
*Pimoa zhigangi* sp. nov.	MW727914	621 bp	Bayi District, Nyingchi, Tibet, China

#### Pimoa anning

Taxon classificationAnimaliaAraneaePimoidae

Zhang & Li sp. nov.

029EA2EB-0D55-5BAB-A719-15D280A91F34

http://zoobank.org/36605D46-1DA7-463C-BDBF-D5FB0C7687DC

Figures 1 , 2 , 54 , 59

##### Type material.

***Holotype*:** ♂ (IZCAS-Ar41921), China, Sichuan, Jinchuan County, Anning Township, Dujiaogou Valley, Mt. Gada, 31.26°N, 101.97°E, ca. 3048 m, 24.XI.2019, Z. Chen leg. ***Paratypes***: 1♂2♀ (IZCAS-Ar41922-Ar41924), same data as holotype.

##### Etymology.

The specific name is a noun in apposition taken from the type locality.

##### Diagnosis.

The male of *Pimoa anning* sp. nov. resembles those of *P. lata* Xu & Li, 2009 (see Zhang and Li 2019: 6, fig. 3A–C) and *P. yele* sp. nov. (Figs 49A, B, 58A) but can be distinguished by the pimoid embolic process that is wider distally than the rest of the process (Fig. 54A) (vs. pimoid embolic process with two jagged tips in *P. lata*, and a broad and robust embolic process, with a distally bifurcate, scaled apex in *P. yele* sp. nov.). Additionally, *P. anning* sp. nov. can be distinguished from *P. lata* by the embolus, which has a short, slender spine proximally (Figs 1A, 54A) (vs. without a spine), and distinguished from *P. yele* sp. nov. by the embolus beginning at the 5:00 o’clock position (Fig. 54A) (vs. 8:00). The female of *P. anning* sp. nov. also resembles those of *P. lata* (see Xu and Li 2009: 57, figs 1–8; Zhang and Li 2019: 6, fig. 4A, B) and *P. yele* sp. nov. (Fig. 50A–D) but can be distinguished from *P. lata* by having the dorsal plate shorter than the ventral plate (Fig. 2B) (vs. dorsal plate longer than the ventral plate) and by the unseparated spermathecae (Fig. 2A–D) (vs. spermathecae separated), and can be distinguished from *P. yele* sp. nov. by the dorsal plate being blunt distally (Fig. 2A–D) (vs. pointed distally).

##### Description.

**Male (*holotype*)**: Total length 5.91. Carapace 2.97 long, 2.38 wide. Abdomen 2.94 long, 1.94 wide. Eye sizes and interdistances: AME 0.15, ALE 0.17, PME 0.16, PLE 0.16; AME-AME 0.09, AME-ALE 0.11, PME-PME 0.13, PME-PLE 0.16. Leg measurements: I: 31.24 (9.03, 10.27, 9.53, 2.41); II: 24.54 (6.63, 7.97, 7.63, 2.31); III: 16.19 (4.94, 4.97, 4.59, 1.69); IV: 21.06 (6.31, 6.75, 6.16, 1.84). Habitus as in Fig. 2E. Carapace brownish with greyish lateral margins; thoracic fovea and radial grooves distinct; sternum yellow. Abdomen black with yellow transverse chevrons, nearly oval. Legs brownish without annulations. Palp (Figs 1A, B, 54A): patella short, ca. 1/2 of tibial length, with a macroseta; tibia short, ca. 1/2 of cymbial length, with several macrosetae and a dorsal process; paracymbium short, ca. 1/3 of cymbial length, finger shaped; pimoid cymbial sclerite U-shaped, ca. 1/3 of cymbial length; cymbial denticulate process short, with more than 7 cuspules; median apophysis slender; conductor distinct; pimoid embolic process slightly wider distally, longer than embolus; embolus beginning at the 5:00 o’clock position, with a short, slender spine proximally; embolic tooth absent.

**Female (*paratype*)**: Total length 5.97. Carapace 2.56 long, 2.34 wide. Abdomen 3.41 long, 2.88 wide. Eye sizes and interdistances: AME 0.16, ALE 0.18, PME 0.14, PLE 0.17; AME-AME 0.06, AME-ALE 0.14, PME-PME 0.11, PME-PLE 0.17. Leg measurements: I: 21.42 (6.01, 7.29, 5.84, 2.28); II: 18.46 (5.16, 6.26, 5.13, 1.91); III: 13.19 (3.81, 4.25, 3.66, 1.47); IV: 17.29 (5.28, 5.66, 4.66, 1.69). Habitus as in Fig. 2F, G. Carapace yellowish with black lateral margins; thoracic fovea and radial grooves distinct; sternum yellow. Abdomen black with yellowish transverse bands. Legs brownish without annulations. Epigyne (Fig. 2A–D): tongue-shaped; ventral plate broad, width ca. 1/2 of length; dorsal plate triangular; copulatory openings distinct; spermathecae nearly rectangular, close to each other; fertilization ducts yellow, laterally oriented.

##### Distribution.

Known only from the type locality, Sichuan, China (Fig. 59).

#### Pimoa bomi

Taxon classificationAnimaliaAraneaePimoidae

Zhang & Li sp. nov.

F70E4DC1-6C54-5540-AA08-A663A93993B7

http://zoobank.org/0DF3A574-A35C-452B-8002-A756A7D34BC9

**Figure 3.**
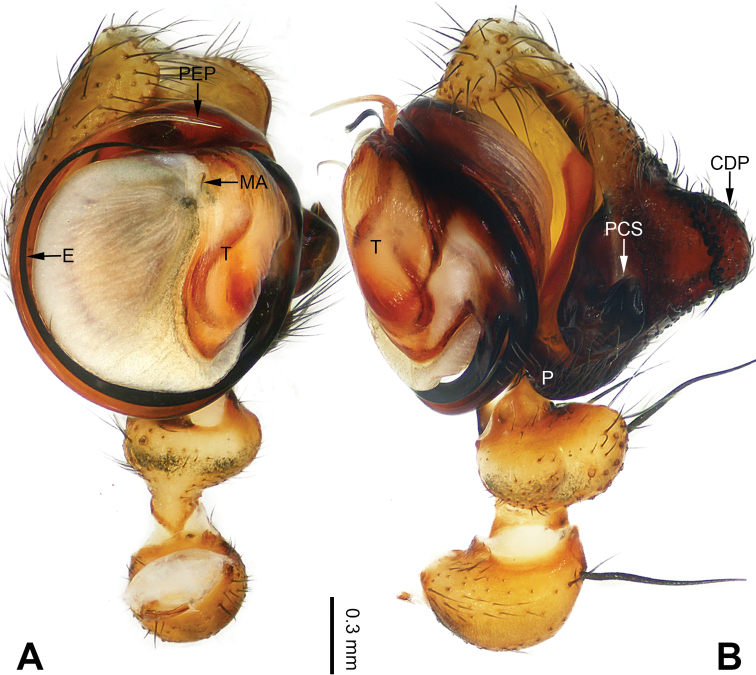
Left palp of *Pimoa bomi* sp. nov., holotype **A** prolateral view **B** retrolateral view. Abbreviations: CDP = cymbial denticulate process; E = embolus; MA = median apophysis; P = paracymbium; PCS = pimoid cymbial sclerite; PEP = pimoid embolic process; T = tegulum. Scale bar: equal for **A, B**.

**Figure 4.**
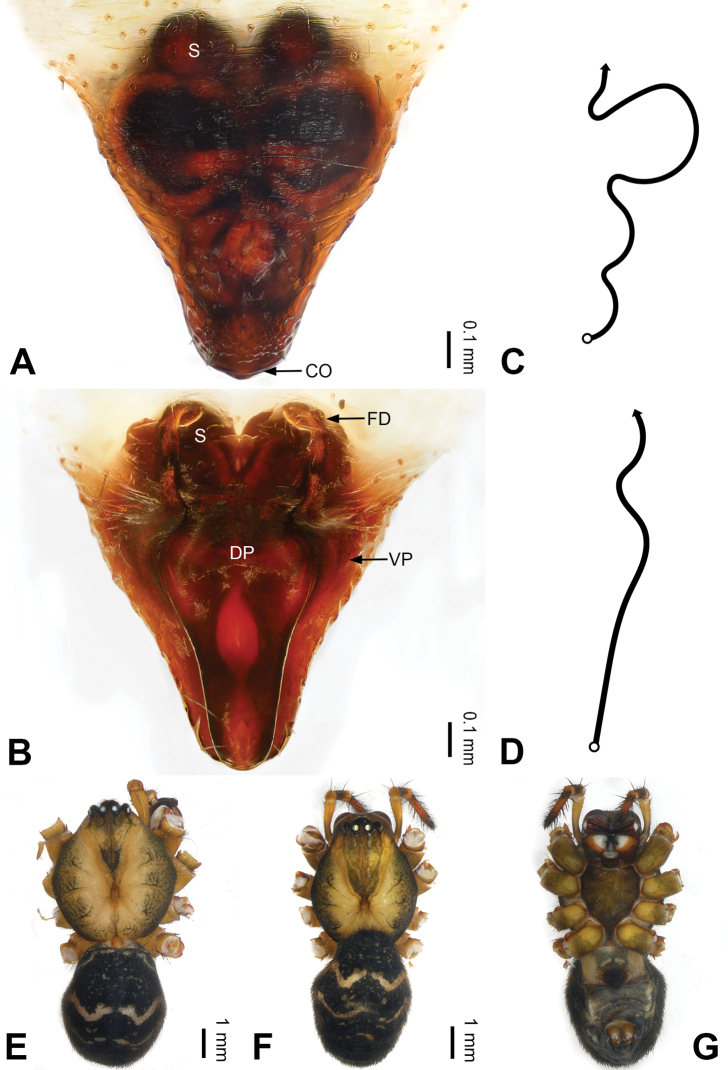
Epigyne and habitus of *Pimoa bomi* sp. nov., female paratype and male holotype **A** epigyne, ventral view **B** vulva, dorsal view **C** schematic course of internal duct system, ventral view **D** schematic course of internal duct system, dorsal view **E** male habitus, dorsal view **F** female habitus, dorsal view **G** female habitus, ventral view. Abbreviations: CO = copulatory opening; DP = dorsal plate of the epigyne; FD = fertilization duct; S = spermatheca; VP = ventral plate of epigyne. Scale bars: equal for **F, G**.

Figures 3 , 4 , 54 , 59

##### Type material.

***Holotype*:** ♂ (IZCAS-Ar41925), China, Tibet, Nyingchi, Bomi County, Karlung Village, 30.04°N, 95.56°E, ca. 3147 m, 26.VII.2019, X. Zhang, Z. Bai and J. Liu leg. ***Paratypes***: 1♂2♀ (IZCAS-Ar41926-Ar41928), same data as holotype.

##### Etymology.

The specific name is a noun in apposition taken from the type locality.

##### Diagnosis.

The male of *Pimoa bomi* sp. nov. resembles those of *P. gyara* sp. nov. (Figs 13A, B, 54D), *P. nyingchi* Zhang & Li, 2020 (see Zhang et al. 2020: 91, fig. 8A–C), and *P. reniformis* Xu & Li, 2007 (see Xu and Li 2007: 493, figs 36–41) but can be distinguished by the short and broad cymbial denticulate process (Fig. 54B) (vs. narrow and distally curved in *P. gyara* sp. nov., short, distally wide, and bent inward in *P. nyingchi*, and robust, broad, and distally curved in *P. reniformis*), and additionally from *P. gyara* sp. nov. and *P. nyingchi* by the wide, V-shaped pimoid cymbial sclerite (Fig. 54B) (vs. narrow and U-shaped in *P. gyara* sp. nov. and narrow in *P. nyingchi*). The female of *P. bomi* sp. nov. also resembles those of *P. nyingchi* (see Zhang et al. 2020: 91, fig. 9A–D) and *P. reniformis* (see Xu and Li 2007: 493, figs 42–47) but can be distinguished from *P. nyingchi* by the distally blunt dorsal plate (Fig. 4B) (vs. pointed) and from *P. reniformis* by the round spermathecae (Fig. 4A) (vs. bean-shaped).

##### Description.

**Male (*holotype*)**: Total length 7.17. Carapace 3.76 long, 3.40 wide. Abdomen 3.41 long, 2.96 wide. Eye sizes and interdistances: AME 0.22, ALE 0.17, PME 0.17, PLE 0.18; AME-AME 0.15, AME-ALE 0.15, PME-PME 0.16, PME-PLE 0.22. Leg measurements: I: 29.04 (8.02, 9.06, 8.56, 3.40); II: 25.64 (7.08, 8.19, 7.56, 2.81); III: 16.29 (5.00, 5.16, 4.63, 1.50); IV: 21.47 (6.09, 6.88, 6.44, 2.06). Habitus as in Fig. 4E. Carapace yellowish with black lateral margins; thoracic fovea and radial grooves distinct; sternum brownish. Abdomen black with yellowish transverse chevrons. Legs brownish with black annulations, especially distinct on legs III and IV. Palp (Figs 3A, B, 54B): patella short, subequal to tibial length, with one retrolateral macroseta; tibia short, ca. 1/3 of cymbial length, with several macrosetae and a dorsal process; paracymbium short, ca. 1/4 of cymbial length, finger shaped; pimoid cymbial sclerite V-shaped, ca. 1/3 of cymbial length; cymbial denticulate process short and broad, with more than 25 cuspules; median apophysis slender; conductor distinct; pimoid embolic process length subequal to embolus; embolus beginning at the 4:00 o’clock position; embolic tooth absent.

**Female (*paratype*)**: Total length 8.20. Carapace 3.88 long, 3.48 wide. Abdomen 4.32 long, 3.20 wide. Eye sizes and interdistances: AME 0.24, ALE 0.25, PME 0.20, PLE 0.22; AME-AME 0.15, AME-ALE 0.19, PME-PME 0.20, PME-PLE 0.24. Leg measurements: I: 25.63 (7.25, 8.78, 6.72, 2.88); II: 21.19 (6.19, 7.22, 5.47, 2.31); III: 15.00 (4.84, 4.85, 3.72, 1.59); IV: 18.60 (5.72, 6.47, 4.53, 1.88). Habitus as in Fig. 4F, G. Carapace yellowish with black lateral margins; thoracic fovea and radial grooves distinct; sternum brownish. Abdomen black with yellowish transverse chevrons. Legs brownish with black annulations. Epigyne (Fig. 4A–D): triangular; ventral plate broad, width subequal to length; dorsal plate tongue shaped; copulatory openings distinct; spermathecae nearly oval, unseparated; fertilization ducts yellowish, medially oriented.

##### Distribution.

Known only from the type locality, Tibet, China (Fig. 59).

#### Pimoa cawarong

Taxon classificationAnimaliaAraneaePimoidae

Zhang & Li sp. nov.

06D5B504-2E31-5032-93C0-A232A355BC49

http://zoobank.org/B201800C-4A44-4624-809E-05793BEB81F9

Figures 5 , 59

##### Type material.

***Holotype*:** ♀ (IZCAS-Ar41929), China, Tibet, Nyingchi, Zayu County, Cawarong Township, 28.55°N, 98.48°E, ca. 4033 m, 31.VII.2019, X. Zhang, Z. Bai and J. Liu leg. ***Paratypes***: 2♀ (IZCAS-Ar41930-Ar41931), same data as holotype.

##### Etymology.

The specific name is a noun in apposition taken from the type locality.

##### Diagnosis.

**Figure 5.**
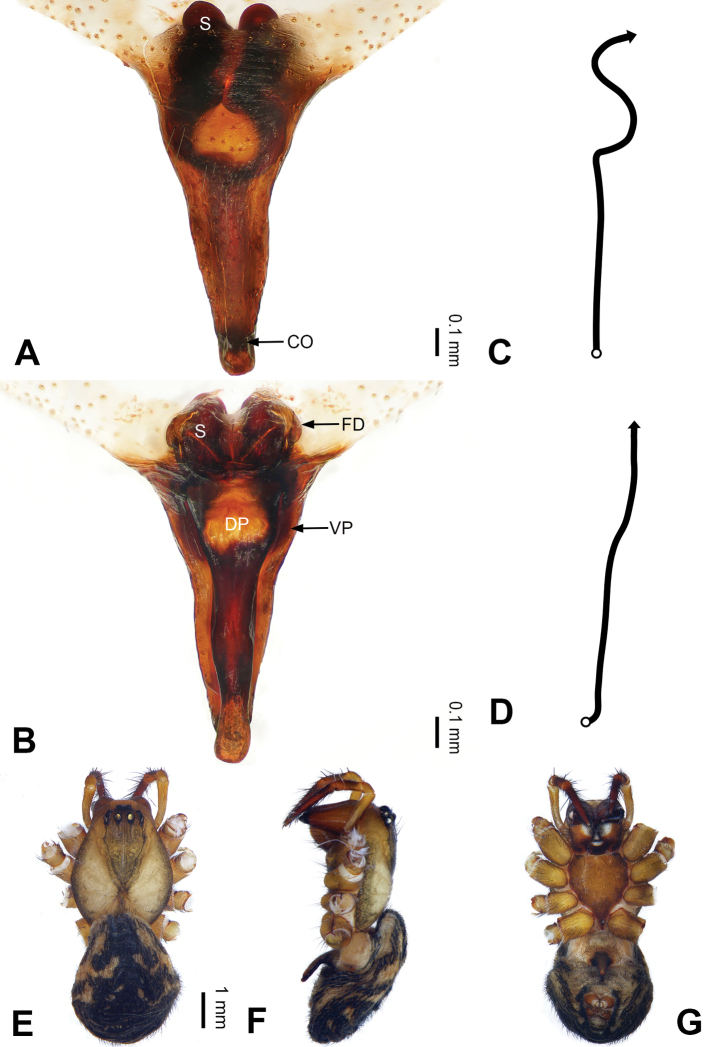
Epigyne and habitus of *Pimoa cawarong* sp. nov., female holotype **A** epigyne, ventral view **B** schematic course of internal duct system, ventral view **C** vulva, dorsal view **D** schematic course of internal duct system, dorsal view **E** female habitus, dorsal view **F** female habitus, lateral view **G** female habitus, ventral view. Abbreviations: CO = copulatory opening; DP = dorsal plate of the epigyne; FD = fertilization duct; S = spermatheca; VP = ventral plate of epigyne. Scale bars: equal for **E–G**.

*Pimoa cawarong* sp. nov. resembles those of *P. nyingchi* (see Zhang et al. 2020: 91, fig. 8A–C) and *P. reniformis* (see Xu and Li 2007: 493, figs 36–41) but can be distinguished from *P. nyingchi* by the width of the ventral plate ca. 1/2 of length (Fig. 5A) (vs. length subequal to width) and by the narrow, distally blunt dorsal plate (Fig. 5B) (vs. wide medially and pointed distally), and can be distinguished from *P. reniformis* by the funnel-shaped epigyne (Fig. 5A) (vs. triangular).

##### Description.

**Female (*holotype*)**: Total length 6.96. Carapace 3.12 long, 2.84 wide. Abdomen 3.84 long, 3.08 wide. Eye sizes and interdistances: AME 0.18, ALE 0.23, PME 0.18, PLE 0.21; AME-AME 0.10, AME-ALE 0.14, PME-PME 0.21, PME-PLE 0.23. Leg measurements: I: 21.44 (6.28, 7.19, 5.53, 2.44); II: 19.09 (5.63, 6.28, 5.02, 2.16); III: 13.07 (3.94, 4.25, 3.41, 1.47); IV: 16.89 (5.28, 5.30, 4.47, 1.84). Habitus as in Fig. 5E–G. Carapace yellowish with black lateral margins; thoracic fovea and radial grooves distinct; sternum brownish. Abdomen black with yellowish transverse bands. Legs brownish with distinct black annulations. Epigyne (Fig. 5A–D): funnel-shaped; ventral and dorsal plates narrow, width ca. 1/2 length; copulatory openings distinct; spermathecae subtriangular, unseparated; fertilization ducts brownish, medially oriented.

**Male**: Unknown.

##### Distribution.

Known only from the type locality, Tibet, China (Fig. 59).

#### Pimoa daman

Taxon classificationAnimaliaAraneaePimoidae

Zhang & Li sp. nov.

B6BE2F90-6D4E-5B83-8354-B1FEFA310996

http://zoobank.org/2ED0107E-F48E-4035-B3DC-95F64025B9A8

Figures 6 , 59

##### Type material.

***Holotype*:** ♀ (IZCAS-Ar41932), Nepal, Narayani District, Daman, Forest near Panorama Resort, 27.60°N, 85.09°E, ca. 2401 m, 22.XI.2016, C. Shrestha leg.

##### Etymology.

The specific name is a noun in apposition taken from the type locality.

##### Diagnosis.

**Figure 6.**
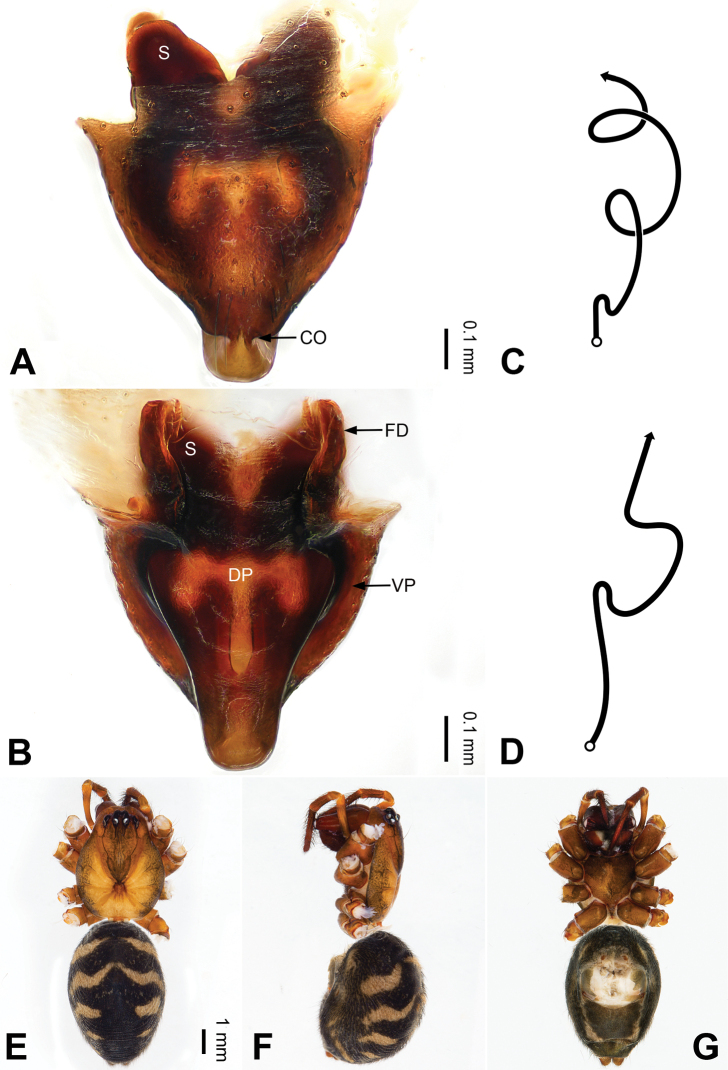
Epigyne and habitus of *Pimoa daman* sp. nov., female holotype **A** epigyne, ventral view **B** schematic course of internal duct system, ventral view **C** vulva, dorsal view **D** schematic course of internal duct system, dorsal view **E** female habitus, dorsal view **F** female habitus, lateral view **G** female habitus, ventral view. Abbreviations: CO = copulatory opening; DP = dorsal plate of the epigyne; FD = fertilization duct; S = spermatheca; VP = ventral plate of epigyne. Scale bars: equal for **E–G**.

*Pimoa daman* sp. nov. resembles those of *P. cona* Zhang & Li, 2020 (see Zhang et al. 2020: 82, fig. 2A–D) and *P. lemenba* Zhang & Li, 2020 (see Zhang et al. 2020: 87, fig. 5A–D) but can be distinguished by the triangular, unseparated spermathecae (Fig. 6A) (vs. oval, separated by ca. 1/3 width of spermatheca in *P. cona*, round and close in *P. lemenba*) and the distally blunt dorsal plate (Fig. 6B) (vs. distally narrow in *P. cona*, distally pointed in *P. lemenba*).

##### Description.

**Female (*holotype*)**: Total length 10.52. Carapace 4.60 long, 3.60 wide. Abdomen 5.92 long, 4.02 wide. Eye sizes and interdistances: AME 0.26, ALE 0.24, PME 0.24, PLE 0.27; AME-AME 0.17, AME-ALE 0.25, PME-PME 0.22, PME-PLE 0.31. Legs missing. Habitus as in Fig. 6E–G. Carapace yellowish with black lateral margins; thoracic fovea and radial grooves distinct; sternum brownish. Abdomen black with yellowish transverse bands. Epigyne (Fig. 6A–D): subtriangular; ventral plate broad, length subequal to width; dorsal plates tongue-shaped, distally blunt, length subequal to width; copulatory openings distinct; spermathecae triangular, unseparated; fertilization ducts yellowish, anteriorly oriented.

**Male**: Unknown.

##### Distribution.

Known only from the type locality, Narayani District, Nepal (Fig. 59).

#### Pimoa danba

Taxon classificationAnimaliaAraneaePimoidae

Zhang & Li sp. nov.

D75B0E36-BC85-5C25-A22F-5382FD99C396

http://zoobank.org/16DA8792-ADB2-490E-97A5-E0687DB32383

Figures 7 , 59

##### Type material.

***Holotype*:** ♀ (IZCAS-Ar41933), China, Sichuan Province, Danba County, Geshizha Township, Jintou Stockaded Village, 31.07°N, 101.66°E, ca. 2639 m, 25.XI.2019, Z. Chen leg. ***Paratypes***: 2♀ (IZCAS-Ar41934-Ar41935), same data as holotype.

##### Etymology.

The specific name is a noun in apposition taken from the type locality.

##### Diagnosis.

**Figure 7.**
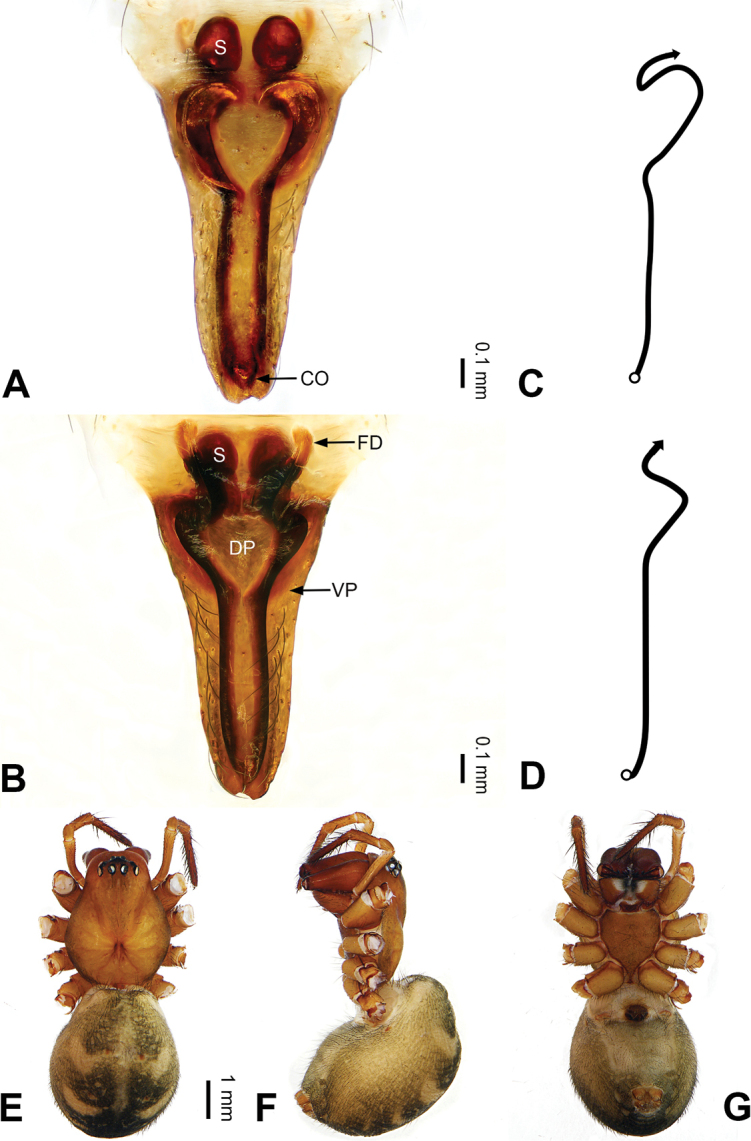
Epigyne and habitus of *Pimoa danba* sp. nov., female holotype **A** epigyne, ventral view **B** schematic course of internal duct system, ventral view **C** vulva, dorsal view **D** schematic course of internal duct system, dorsal view **E** female habitus, dorsal view **F** female habitus, lateral view **G** female habitus, ventral view. Abbreviations: CO = copulatory opening; DP = dorsal plate of the epigyne; FD = fertilization duct; S = spermatheca; VP = ventral plate of epigyne. Scale bars: equal for **E–G**.

*Pimoa danba* sp. nov. resembles those of *P. cawarong* sp. nov. (Fig. 5A–D) and *P. indiscreta* Hormiga, 1994 (see Hormiga 1994a: 66, figs 248–255) but can be distinguished by the nearly round spermathecae separated by ca. 1/4 width of a spermatheca (Fig. 7A) (vs. subtriangular, unseparated in *P. cawarong* sp. nov., and nearly oval, separated by a short distance in *P. indiscreta*), by the distally narrow ventral plate with a depression (Fig. 7A) (vs. without depression in *P. cawarong* sp. nov., and triangular, without depression in *P. indiscreta*), and also from *P. indiscreta* by the laterally oriented fertilization ducts (Fig. 7B) (vs. medially oriented).

##### Description.

**Female (*holotype*)**: Total length 6.73. Carapace 3.24 long, 2.78 wide. Abdomen 3.49 long, 3.16 wide. Eye sizes and interdistances: AME 0.16, ALE 0.21, PME 0.17, PLE 0.24; AME-AME 0.12, AME-ALE 0.15, PME-PME 0.15, PME-PLE 0.18. Leg measurements: I: 26.53 (7.69, 9.15, 6.97, 2.72); II: 22.09 (6.27, 7.66, 6.19, 1.97); III: 16.17 (4.78, 5.29, 4.41, 1.69); IV: 21.75 (6.41, 7.88, 5.52, 1.94). Habitus as in Fig. 7E–G. Carapace yellowish with black lateral margins; thoracic fovea and radial grooves distinct; sternum brownish. Abdomen grayish with yellowish transverse chevrons. Legs brownish without annulations. Epigyne (Fig. 7A–D): subtriangular; ventral plate tongue-shaped, with a depression distally, width ca. 1/2 length; dorsal plates narrow; copulatory openings distinct; spermathecae nearly round, separated by ca. 1/4 width of spermatheca; fertilization ducts yellowish, laterally oriented.

**Male**: Unknown.

##### Distribution.

Known only from the type locality, Sichuan, China (Fig. 59).

#### Pimoa deqen

Taxon classificationAnimaliaAraneaePimoidae

Zhang & Li sp. nov.

2F9DC13E-BA13-5F4E-BF7F-F8E482FBE8B9

http://zoobank.org/A7375F2E-C799-4FFA-95EA-42B92CEAFCDA

Figures 8 , 59

##### Type material.

***Holotype*:** ♀ (IZCAS-Ar41936), China, Yunnan, Diqing Tibetan Autonomous Prefecture, Shangrila to Deqen County, Tongduishui, 28.30°N, 99.15°E, ca. 3309 m, 17.IX.2020, Z. Chen leg. ***Paratype***: 1♀ (IZCAS-Ar41937), same data as holotype.

##### Etymology.

The specific name is a noun in apposition taken from the type locality.

##### Diagnosis.

**Figure 8.**
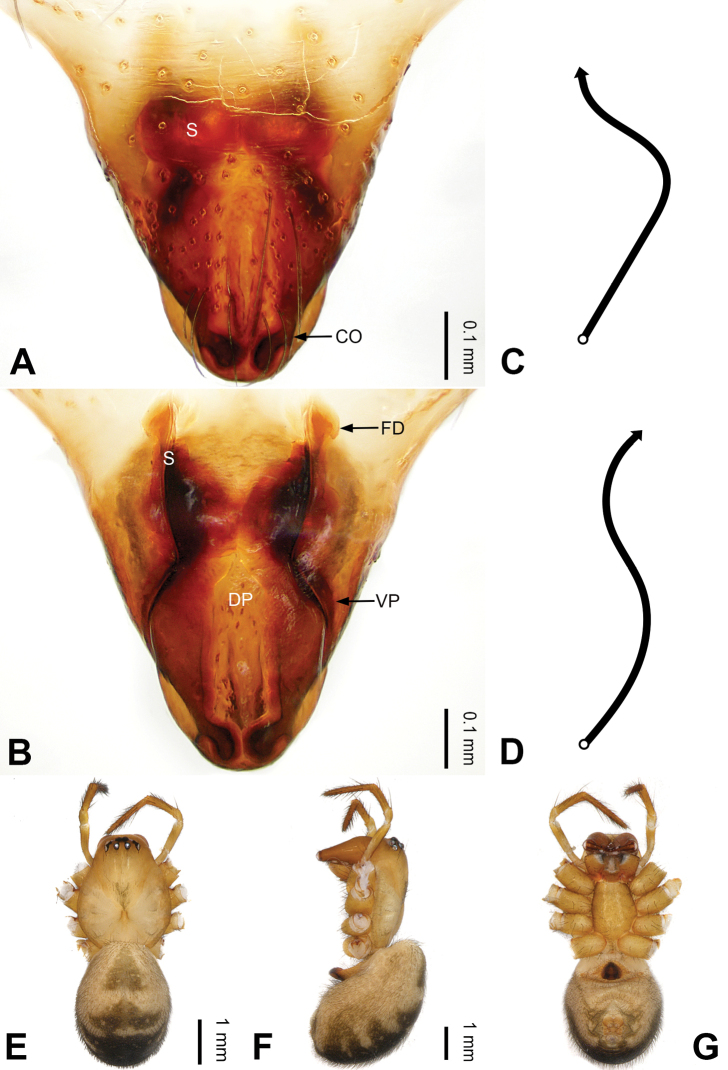
Epigyne and habitus of *Pimoa deqen* sp. nov., female holotype **A** epigyne, ventral view **B** schematic course of internal duct system, ventral view **C** vulva, dorsal view **D** schematic course of internal duct system, dorsal view **E** female habitus, dorsal view **F** female habitus, lateral view **G** female habitus, ventral view. Abbreviations: CO = copulatory opening; DP = dorsal plate of the epigyne; FD = fertilization duct; S = spermatheca; VP = ventral plate of epigyne. Scale bars: equal for **E–G**.

*Pimoa deqen* sp. nov. resembles those of *P. lihengae* Griswold, Long & Hormiga, 1999 (see Griswold et al. 1999: 91–97, figs 18–21) and *P. wanglangensis* Yuan, Zhao & Zhang, 2019 (see Yuan et al. 2019: 27, fig. 22G, H) but can be distinguished from *P. lihengae* by the tongue-shaped dorsal plate (Fig. 8B) (vs. triangular) and from *P. wanglangensis* by the unseparated spermathecae (Fig. 8A) (vs. separated by a short distance).

##### Description.

**Female (*holotype*)**: Total length 5.51. Carapace 2.19 long, 2.09 wide. Abdomen 3.32 long, 2.22 wide. Eye sizes and interdistances: AME 0.13, ALE 0.13, PME 0.11, PLE 0.14; AME-AME 0.08, AME-ALE 0.12, PME-PME 0.15, PME-PLE 0.16. Leg measurements: I: 15.85 (4.50, 5.66, 3.97, 1.72); II: 14.29 (4.13, 4.78, 3.75, 1.63); III: 11.36 (3.38, 3.74, 3.02, 1.22); IV: 14.20 (4.28, 4.66, 3.72, 1.54). Habitus as in Fig. 8E–G. Carapace yellowish; thoracic fovea and radial grooves distinct; sternum brownish. Abdomen black with yellowish transverse bands. Legs brownish without annulations. Epigyne (Fig. 8A–D): subtriangular; ventral plate broad, length subequal to width; dorsal plates tongue-shaped, length subequal to width; copulatory openings distinct; spermathecae oval, unseparated; fertilization ducts yellowish, medially oriented.

**Male**: Unknown.

##### Distribution.

Known only from the type locality, Yunnan, China (Fig. 59).

#### Pimoa dongjiu

Taxon classificationAnimaliaAraneaePimoidae

Zhang & Li sp. nov.

6D193C6E-71C1-572B-8099-2C91CB8F90CE

http://zoobank.org/12EB1E82-3EE0-4060-9E75-496C980BBCF4

**Figure 9.**
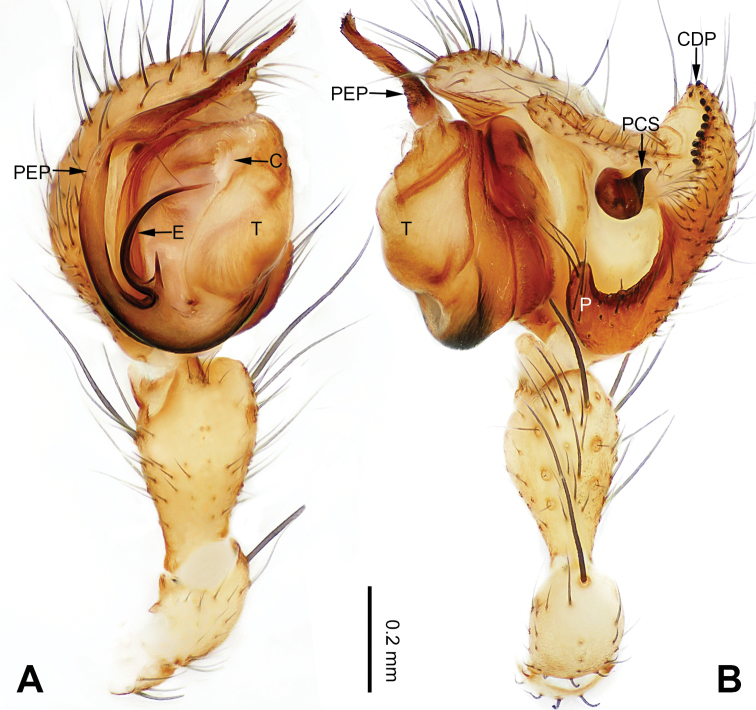
Left palp of *Pimoa dongjiu* sp. nov., holotype **A** prolateral view **B** retrolateral view. Abbreviations: C = conductor; CDP = cymbial denticulate process; E = embolus; P = paracymbium; PCS = pimoid cymbial sclerite; PEP = pimoid embolic process; T = tegulum. Scale bar: equal for **A, B**.

**Figure 10.**
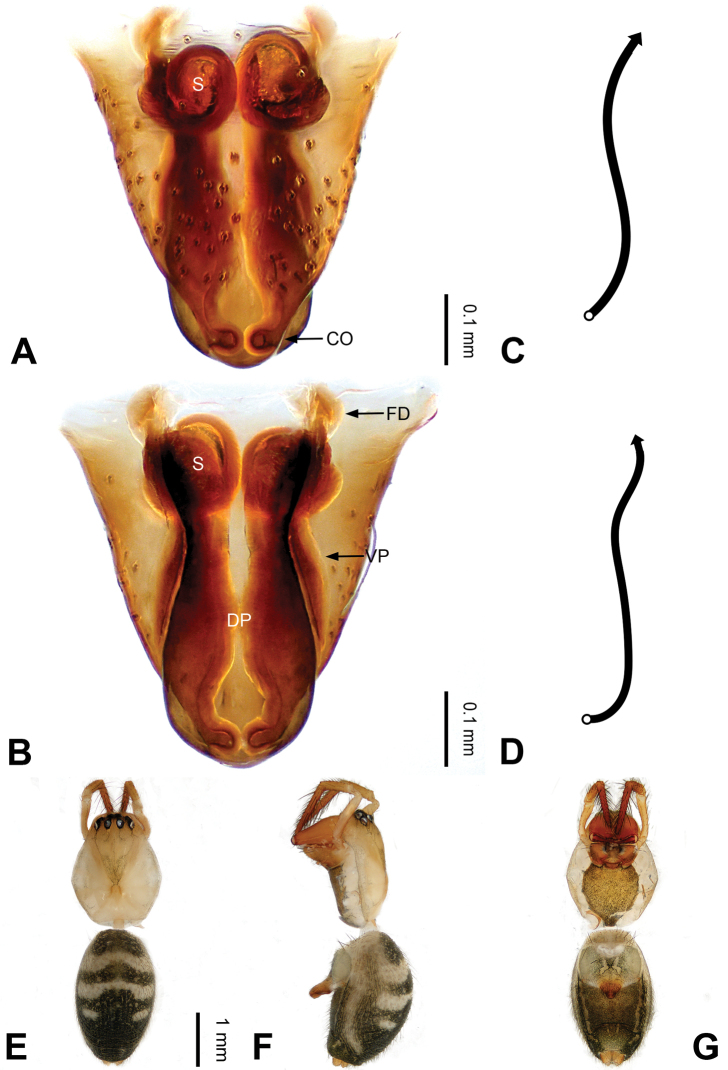
Epigyne and habitus of *Pimoa dongjiu* sp. nov., female holotype **A** epigyne, ventral view **B** schematic course of internal duct system, ventral view **C** vulva, dorsal view **D** schematic course of internal duct system, dorsal view **E** female habitus, dorsal view **F** female habitus, lateral view **G** female habitus, ventral view. Abbreviations: CO = copulatory opening; DP = dorsal plate of the epigyne; FD = fertilization duct; S = spermatheca; VP = ventral plate of epigyne. Scale bars: equal for **E–G**.

Figures 9 , 10 , 54 , 59

##### Type material.

***Holotype*:** ♂ (IZCAS-Ar41938), China, Tibet, Nyingchi, Bayi District, Lunang Town, Dongjiu Village, 29.82°N, 94.74°E, ca. 3117 m, 29.IX.2020, Z. Chen leg. ***Paratype***: 1♀ (IZCAS-Ar41939), China, Tibet, Nyingchi, Bomi County, Yuri Township, the bridge in Dazeshan Village, 30.28°N, 95.28°E, ca. 3199 m, 27.IX.2020, Z. Chen leg.

##### Etymology.

The specific name is a noun in apposition taken from the type locality.

##### Diagnosis.

The male of *Pimoa dongjiu* sp. nov. resembles those of *P. anatolica* Hormiga, 1994 (see Xu and Li 2007: 484, figs 1–8) and *P. lihengae* (see Griswold et al. 1999: 93, figs 15–17) but can be distinguished by the embolus beginning at the 6:30 o’clock position with a short, slender spine proximally (Fig. 54C) (vs. beginning at the 6:00 o’clock position, without a spine in *P. anatolica* and *P. lihengae*), by having the pimoid embolic process longer than the embolus, distally serrate and with scales (Fig. 54C) (vs. shorter than embolus, with a short, sharp branch in *P. anatolica* and shorter than embolus in *P. lihengae*), and also distinguished from *P. lihengae* by the broad cymbial denticulate process (Fig. 54C) (vs. distally bent). The female of *P. dongjiu* sp. nov. resembles those of *P. lihengae* (see Griswold et al. 1999: 93, figs 18–21) and *P. wanglangensis* (see Yuan et al. 2019: 27, fig. 22A–H) but can be distinguished by the distally blunt dorsal plate (Fig. 10B) (vs. distally narrow in *P. lihengae* and *P. wanglangensis*) and also from *P. wanglangensis* by the unseparated spermathecae (Fig. 10A) (vs. slightly separated).

##### Description.

**Male (*holotype*)**: Total length –. Carapace missing. Abdomen 2.38 long, 1.44 wide. Legs missing. Abdomen grey with yellowish transverse bands, nearly oval. Palp (Figs 9A, B, 54C): patella short, ca. 1/2 of tibial length, with one retrolateral macroseta; tibia short, subequal to cymbial length, with several macrosetae and a dorsal process; paracymbium short, ca. 1/3 of cymbial length, hook-shaped; pimoid cymbial sclerite U-shaped, ca. 1/3 of cymbial length; cymbial denticulate process broad, with more than 8 cuspules; median apophysis slender; conductor distinct; pimoid embolic process broad, robust, distally serrate and with scales, longer than embolus; embolus beginning at the 7:00 o’clock position, with short, slender spine proximally; embolic tooth absent.

**Female (*paratype*)**: Total length 4.51. Carapace 2.02 long, 1.69 wide. Abdomen 2.49 long, 1.47 wide. Eye sizes and interdistances: AME 0.10, ALE 0.15, PME 0.12, PLE 0.14; AME-AME 0.07, AME-ALE 0.05, PME-PME 0.11, PME-PLE 0.13. Legs missing. Habitus as in Fig. 10E–G. Carapace yellowish; thoracic fovea and radial grooves distinct; sternum brownish. Abdomen greyish with yellowish transverse bands. Epigyne (Fig. 10A–D): subtriangular; ventral plate broad, width subequal to length; dorsal plate tongue shaped, distally blunt; copulatory openings distinct; spermathecae oval, unseparated; fertilization ducts yellowish, laterally oriented.

##### Distribution.

Known only from the type locality, Tibet, China (Fig. 59).

#### Pimoa guiqing

Taxon classificationAnimaliaAraneaePimoidae

Zhang & Li sp. nov.

B5E52E7A-587A-5B2C-85AD-99CF1AB20C90

http://zoobank.org/CC819285-03F2-4F81-94BB-2997F472D861

Figures 11 , 59

##### Type material.

***Holotype*:** ♀ (IZCAS-Ar41940), China, Gansu, Tianshui, Wushan County, Mt. Guiqing, 34.65°N, 104.49°E, ca. 1922 m, 13.VII.2020, Y. Lin and Z. Wang leg. ***Paratype***: 1♀ (IZCAS-Ar41941), same data as holotype.

##### Etymology.

The specific name is a noun in apposition taken from the type locality.

##### Diagnosis.

**Figure 11.**
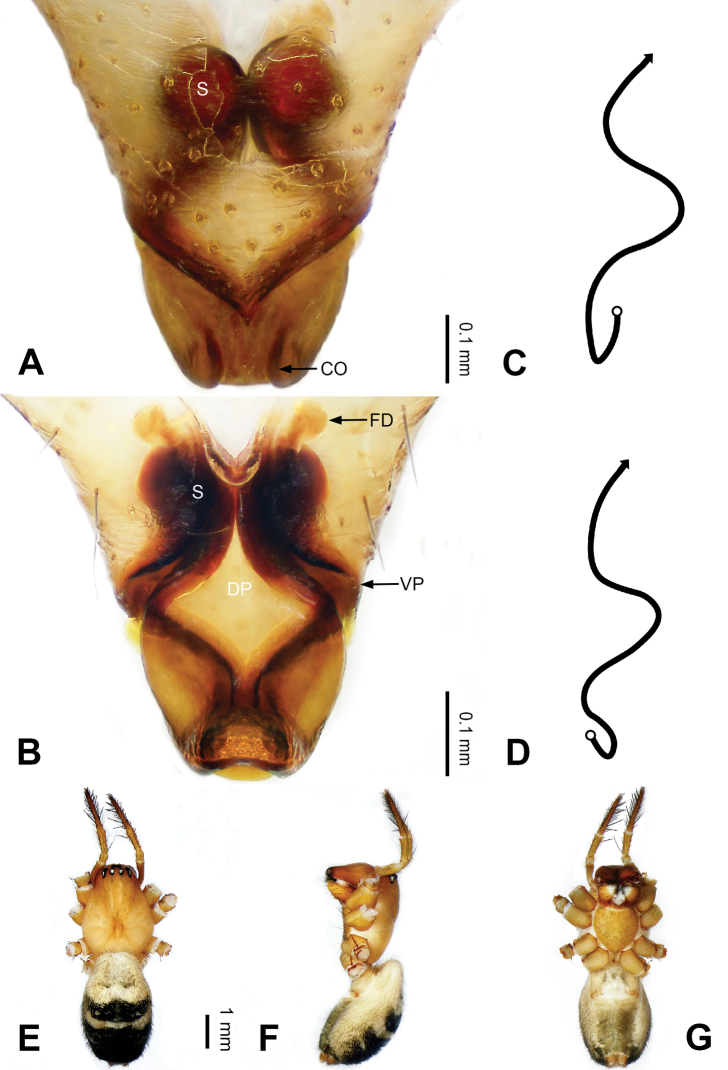
Epigyne and habitus of *Pimoa guiqing* sp. nov., female holotype **A** epigyne, ventral view **B** schematic course of internal duct system, ventral view **C** vulva, dorsal view **D** schematic course of internal duct system, dorsal view **E** female habitus, dorsal view **F** female habitus, lateral view **G** female habitus, ventral view. Abbreviations: CO = copulatory opening; DP = dorsal plate of the epigyne; FD = fertilization duct; S = spermatheca; VP = ventral plate of epigyne. Scale bars: equal for **E–G**.

*Pimoa guiqing* sp. nov. can be distinguished from other congeners except for *P. binchuanensis* Zhang & Li, 2019 (see Zhang and Li 2019: 3, fig. 2A, B) by the tongue-shaped, distally curved dorsal plate (Fig. 11B) (vs. with an oval tip and trapezoidal basal part in *P. binchuanensis*) and by the unseparated spermathecae (Fig. 11A) (vs. separated by ca. 1/2 the width in *P. binchuanensis*).

##### Description.

**Female (*holotype*)**: Total length 5.79. Carapace 2.63 long, 2.16 wide. Abdomen 3.16 long, 2.13 wide. Eye sizes and interdistances: AME 0.12, ALE 0.14, PME 0.12, PLE 0.16; AME-AME 0.08, AME-ALE 0.14, PME-PME 0.14, PME-PLE 0.15. Leg measurements: I: – (5.59, –, –, –); II: 17.64 (4.90, 6.12, 4.81, 1.81); III: 13.07 (3.97, 4.10, 3.56, 1.44); IV: – (5.12, –, –, –). Habitus as in Fig. 11E–G. Carapace yellowish; thoracic fovea and radial grooves distinct; sternum yellow. Abdomen proximally yellowish with grayish transverse bands. Legs brownish without annulations. Epigyne (Fig. 11A–D): triangular; ventral plate broad, length subequal to width; dorsal plate tongue-shaped, with a curved tip distally, length subequal to width; copulatory openings distinct; spermathecae round, unseparated; fertilization ducts yellow, laterally oriented.

**Male**: Unknown.

##### Distribution.

Known only from the type locality, Gansu, China (Fig. 59).

#### Pimoa gyaca

Taxon classificationAnimaliaAraneaePimoidae

Zhang & Li sp. nov.

888B57AE-49A0-5A9A-B05F-AE1BAF9AA94D

http://zoobank.org/1C178C6A-BCDB-4E7D-8E9D-9F8476D9A008

Figures 12 , 59

##### Type material.

***Holotype*:** ♀ (IZCAS-Ar41942), China, Tibet, Lhoka, Gyaca County, on the way to the Lhamo Latso Lake, 29.39°N, 92.75°E, ca. 4435 m, 11.VIII.2019, X. Zhang, Z. Bai and J. Liu leg. ***Paratypes***: 2♀ (IZCAS-Ar41943-Ar41944), same data as holotype.

##### Etymology.

The specific name is a noun in apposition taken from the type locality.

##### Diagnosis.

**Figure 12.**
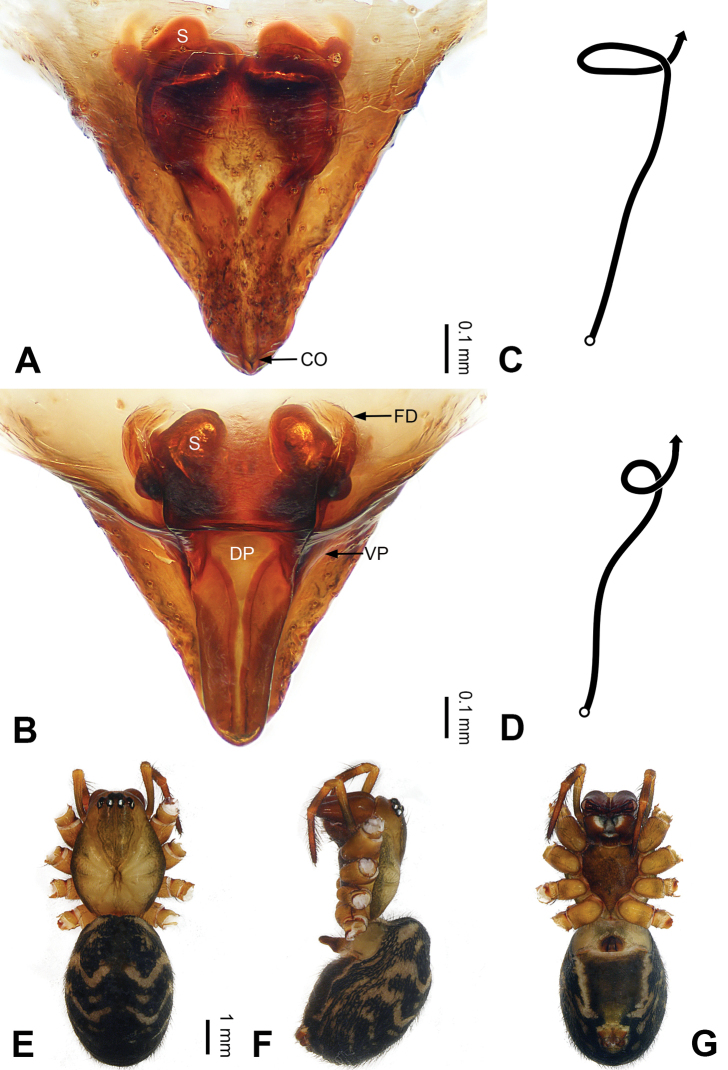
Epigyne and habitus of *Pimoa gyaca* sp. nov., female holotype **A** epigyne, ventral view **B** schematic course of internal duct system, ventral view **C** vulva, dorsal view **D** schematic course of internal duct system, dorsal view **E** female habitus, dorsal view **F** female habitus, lateral view **G** female habitus, ventral view. Abbreviations: CO = copulatory opening; DP = dorsal plate of the epigyne; FD = fertilization duct; S = spermatheca; VP = ventral plate of epigyne. Scale bars: equal for **E–G**.

*Pimoa gyaca* sp. nov. resembles those of *P. crispa* (Fage, 1946) (see Hormiga 1994a: 63, figs 239–247) and *P. mainling* Zhang & Li, 2020 (see Zhang et al. 2020: 89, fig. 7A–D) but can be distinguished from *P. crispa* by the triangular ventral plate (Fig. 12A) (vs. distally blunt) and from *P. mainling* by the triangular epigyne (Fig. 12A) (vs. funnel shaped) and also by the spermathecae separated by the width of a spermatheca (Fig. 12A) (vs. ca. 1/3 width of a spermatheca).

##### Description.

**Female (*holotype*)**: Total length 8.00. Carapace 3.68 long, 2.78 wide. Abdomen 4.32 long, 3.36 wide. Eye sizes and interdistances: AME 0.19, ALE 0.20, PME 0.18, PLE 0.19; AME-AME 0.14, AME-ALE 0.19, PME-PME 0.15, PME-PLE 0.19. Leg measurements: I: 26.76 (7.74, 9.00, 7.46, 2.56); II: 22.66 (6.44, 7.47, 6.31, 2.44); III: 15.82 (4.82, 5.15, 4.47, 1.38); IV: 20.78 (6.47, 6.50, 5.78, 2.03). Habitus as in Fig. 12E–G. Carapace yellowish with black lateral margins; thoracic fovea and radial grooves distinct; sternum brownish. Abdomen black with yellowish transverse bands. Legs brownish with black annulations. Epigyne (Fig. 12A–D): triangular; ventral plates broad, distally pointed, length subequal to width; dorsal plate narrow, tongue-shaped; copulatory openings distinct; spermathecae oval, separated by width of spermatheca; fertilization ducts yellowish, laterally oriented.

**Male**: Unknown.

##### Distribution.

Known only from the type locality, Tibet, China (Fig. 59).

#### Pimoa gyara

Taxon classificationAnimaliaAraneaePimoidae

Zhang & Li sp. nov.

83720DBC-7EC6-57F4-89A0-6F213F736F6B

http://zoobank.org/6F5D6566-2A51-4B2C-9BCB-A2ABDEC0C097

**Figure 13.**
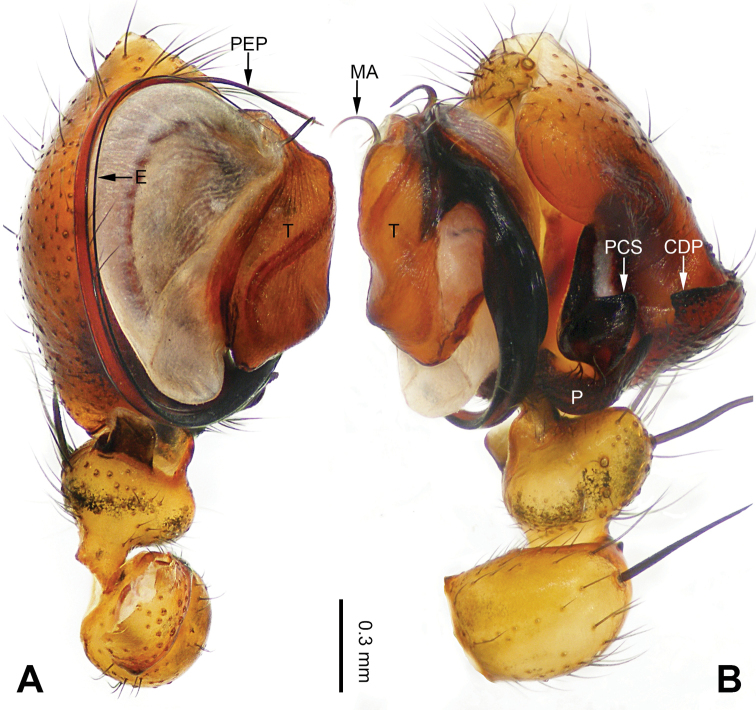
Left palp of *Pimoa gyara* sp. nov., holotype **A** prolateral view **B** retrolateral view. Abbreviations: CDP = cymbial denticulate process; E = embolus; MA = median apophysis; P = paracymbium; PCS = pimoid cymbial sclerite; PEP = pimoid embolic process; T = tegulum. Scale bar: equal for **A, B**.

**Figure 14.**
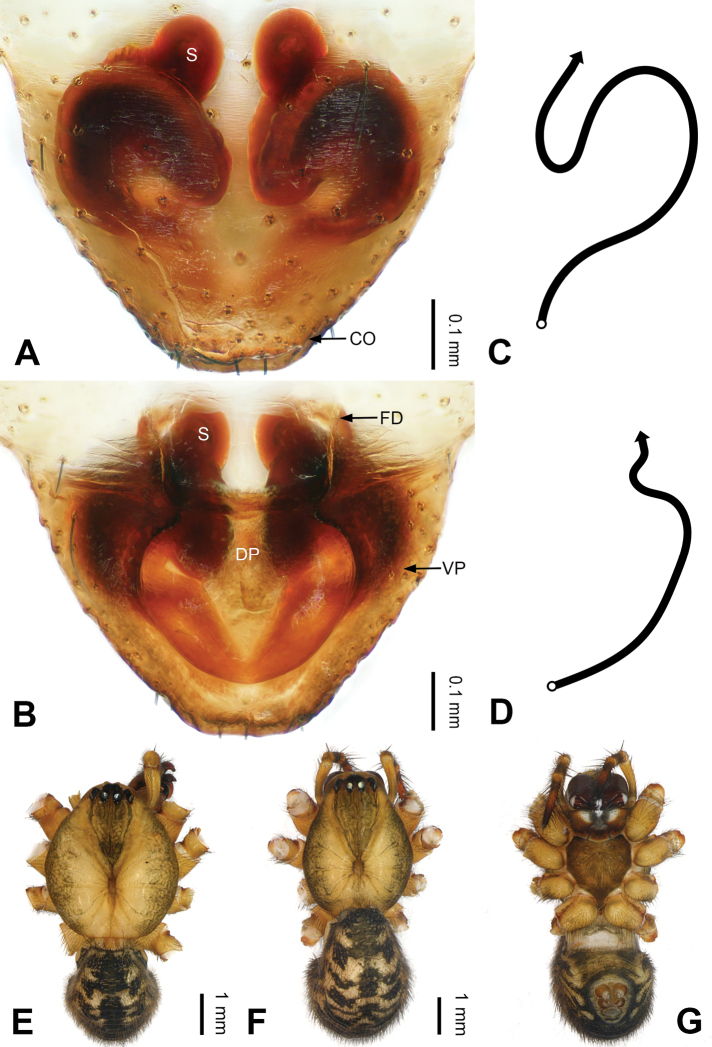
Epigyne and habitus of *Pimoa gyara* sp. nov., female paratype and male holotype **A** epigyne, ventral view **B** vulva, dorsal view **C** schematic course of internal duct system, ventral view **D** schematic course of internal duct system, dorsal view **E** male habitus, dorsal view **F** female habitus, dorsal view **G** female habitus, ventral view. Abbreviations: CO = copulatory opening; DP = dorsal plate of the epigyne; FD = fertilization duct; S = spermatheca; VP = ventral plate of epigyne. Scale bars: equal for **F, G**.

Figures 13 , 14 , 54 , 59

##### Type material.

***Holotype*:** ♂ (IZCAS-Ar41945), China, Tibet, Nyingchi, Gongbo’gyamda County, Gyara Village, 30.01°N, 93.78°E, ca. 3460 m, 14.VII.2019, X. Zhang, Z. Bai and J. Liu leg. ***Paratypes***: 1♂2♀ (IZCAS-Ar41946-Ar41948), same data as holotype.

##### Etymology.

The specific name is a noun in apposition taken from the type locality.

##### Diagnosis.

The male of *Pimoa gyara* sp. nov. resembles those of *P. nyingchi* (see Zhang et al. 2020: 91, fig. 8A–C) and *P. reniformis* (see Xu and Li 2007: 493, figs 36–41) but can be distinguished from *P. nyingchi* by the narrow and distally curved cymbial denticulate process (Fig. 54D) (vs. flat distally and wide) and from *P. reniformis* by the U-shaped pimoid cymbial sclerite (Fig. 54D) (vs. triangular). The female of *P. gyara* sp. nov. also resembles *P. nyingchi* (see Zhang et al. 2020: 91, fig. 9A–D) but can be distinguished by the distally blunt dorsal plate (Fig. 14B) (vs. pointed) and by the spermathecae separated by ca. 1/2 width of spermatheca (Fig. 14A) (vs. close to each other).

##### Description.

**Male (*holotype*)**: Total length 6.25. Carapace 3.81 long, 3.28 wide. Abdomen 2.44 long, 2.28 wide. Eye sizes and interdistances: AME 0.20, ALE 0.21, PME 0.17, PLE 0.18; AME-AME 0.11, AME-ALE 0.14, PME-PME 0.17, PME-PLE 0.21. Leg measurements: I: 25.04 (6.88, 8.22, 7.13, 2.81); II: 21.63 (6.03, 7.16, 6.03, 2.41); III: 15.38 (4.72, 4.91, 4.34, 1.41); IV: 19.62 (5.66, 6.47, 5.65, 1.84). Habitus as in Fig. 14E. Carapace yellowish with black lateral margins; thoracic fovea and radial grooves distinct; sternum brownish. Abdomen black with yellow transverse chevrons, nearly oval. Legs brownish without annulations. Palp (Figs 13A, B, 54D): patella short, almost as long as tibial length, with one retrolateral macroseta; tibia short, ca. 1/3 of cymbial length, with several macrosetae and a dorsal process; paracymbium short, ca. 1/4 of cymbial length, finger-shaped; pimoid cymbial sclerite U-shaped, ca. 1/2 of cymbial length; cymbial denticulate process short, distally narrow and curved, with more than 20 cuspules; median apophysis slender; conductor distinct; pimoid embolic process distally pointed, length subequal to embolus; embolus beginning at the 3:00 o’clock position; embolic tooth absent.

**Female (*paratype*)**: Total length 7.40. Carapace 3.44 long, 2.97 wide. Abdomen 3.96 long, 2.59 wide. Eye sizes and interdistances: AME 0.22, ALE 0.23, PME 0.16, PLE 0.22; AME-AME 0.09, AME-ALE 0.16, PME-PME 0.15, PME-PLE 0.24. Leg measurements: I: 19.60 (5.47, 6.78, 5.13, 2.22); II: 16.89 (4.94, 5.75, 4.39, 1.81); III: 12.04 (3.84, 4.16, 3.01, 1.03); IV: 16.82 (5.09, 5.69, 4.38, 1.66). Habitus as in Fig. 14F, G. Carapace yellowish with black lateral margins; thoracic fovea and radial grooves distinct; sternum brownish. Abdomen black with yellowish transverse bands. Legs brownish without annulations. Epigyne (Fig. 14A–D): triangular; ventral plate broad, width subequal to length; dorsal plate tongue-shaped, shorter than ventral plate; copulatory openings indistinct; spermathecae oval, separated by ca. 1/3 width of spermatheca; fertilization ducts yellowish, anteriorly oriented.

##### Distribution.

Known only from the type locality, Tibet, China (Fig. 59).

#### Pimoa gyirong

Taxon classificationAnimaliaAraneaePimoidae

Zhang & Li sp. nov.

351E7E4A-AC8F-5EE8-A18B-FFBC807A5A94

http://zoobank.org/20F3098C-37B3-44A5-89BC-637AAEE09AC2

**Figure 15.**
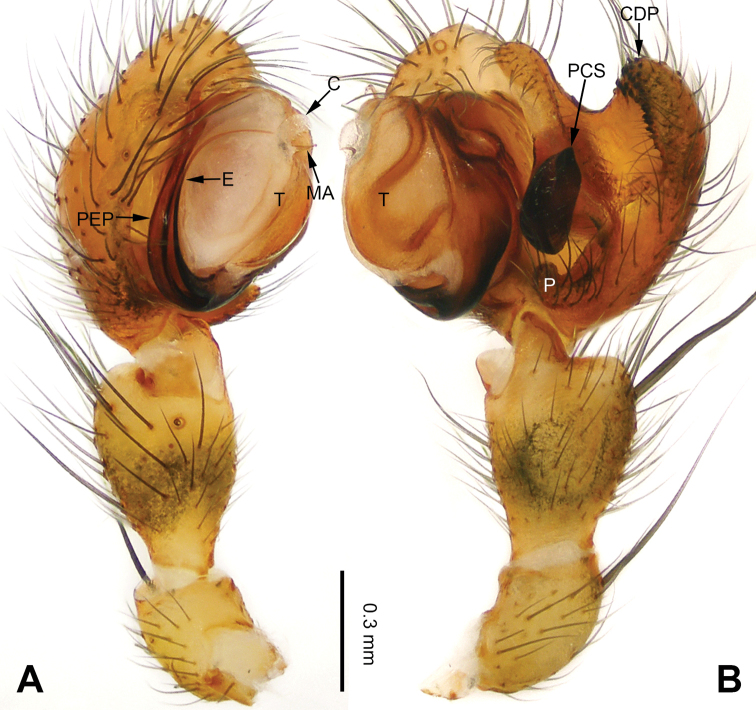
Left palp of *Pimoa gyirong* sp. nov., holotype **A** prolateral view **B** retrolateral view. Abbreviations: C = conductor; CDP = cymbial denticulate process; E = embolus; MA = median apophysis; P = paracymbium; PCS = pimoid cymbial sclerite; PEP = pimoid embolic process; T = tegulum. Scale bar: equal for **A, B**.

**Figure 16.**
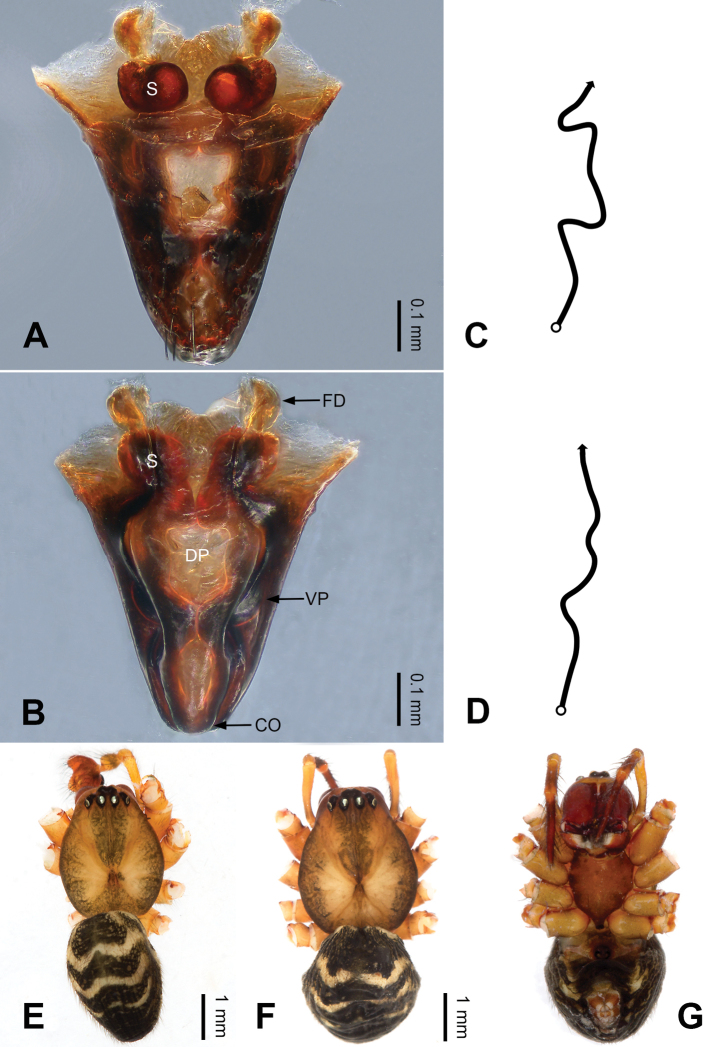
Epigyne and habitus of *Pimoa gyirong* sp. nov., female paratype and male holotype **A** epigyne, ventral view **B** vulva, dorsal view **C** schematic course of internal duct system, ventral view **D** schematic course of internal duct system, dorsal view **E** male habitus, dorsal view **F** female habitus, dorsal view **G** female habitus, ventral view. Abbreviations: CO = copulatory opening; DP = dorsal plate of the epigyne; FD = fertilization duct; S = spermatheca; VP = ventral plate of epigyne. Scale bars: equal for **F, G**.

Figures 15 , 16 , 55 , 59

##### Type material.

***Holotype*:** ♂ (IZCAS-Ar41949), China, Tibet, Shigatse, Gyirong County, Gyirong Town, near Lhanggyi Tso Holy Lake, 28.41°N, 85.40°E, ca. 3909 m, 7.VII.2019, X. Zhang, Z. Bai and J. Liu leg. ***Paratypes***: 1♂2♀ (IZCAS-Ar41950-Ar41952), same data as holotype.

##### Etymology.

The specific name is a noun in apposition taken from the type locality.

##### Diagnosis.

The male of *Pimoa gyirong* sp. nov. resembles those of *P. crispa* (see Hormiga 1994a: 63, figs 233–238; Hormiga 1994b: fig. 1A, B) and *P. rongxar* Zhang & Li, 2020 (see Zhang et al. 2020: 94, fig. 10A–C) but can be distinguished from *P. crispa* by the distally narrower cymbial denticulate process (Fig. 55A) (vs. wider) and distinguished from *P. rongxar* by the broad cymbial denticulate process, with many cuspules distally (Figs 15B, 55A) (vs. distally narrow, with few cuspules) and the shorter pimoid cymbial sclerite (Fig. 55A) (vs. large and wide subdistally). The female of *P. gyirong* sp. nov. resembles those of *P. nyingchi* (see Zhang et al. 2020: 91, fig. 9A–D) and *P. reniformis* (see Xu and Li 2007: 493, figs 42–47) but can be distinguished from *P. nyingchi* by the distally blunt dorsal plate (Fig. 16B) (vs. pointed) and distinguished from *P. reniformis* by the spermathecae separated by ca. 1/4 the width of a spermatheca (Fig. 16A) (vs. unseparated).

##### Description.

**Male (*holotype*)**: Total length 5.41. Carapace 2.63 long, 2.31 wide. Abdomen 2.78 long, 2.03 wide. Eye sizes and interdistances: AME 0.18, ALE 0.19, PME 0.19, PLE 0.14; AME-AME 0.13, AME-ALE 0.14, PME-PME 0.11, PME-PLE 0.18. Leg measurements: I: 28.22 (7.75, 9.19, 8.34, 2.94); II: 24.19 (6.66, 7.91, 7.03, 2.59); III: 14.61 (4.34, 4.59, 4.34, 1.34); IV: 19.46 (5.59, 6.25, 5.59, 2.03). Habitus as in Fig. 16E. Carapace yellowish with black lateral margins; thoracic fovea and radial grooves distinct; sternum brownish. Abdomen black with yellow chevrons, nearly oval. Legs brownish with black annulations, especially distinct on legs III and IV. Palp (Figs 15A, B, 55A): patella short, ca. 1/2 of tibial length, with one retrolateral macroseta; tibia long, ca. 1/2 of cymbial length, with several macrosetae and a dorsal process; paracymbium short, ca. 1/3 of cymbial length, hook-shaped; pimoid cymbial sclerite V-shaped, distally pointed, ca. 1/2 of cymbial length; cymbial denticulate process long and distally blunt, with more than 47 cuspules; median apophysis slender; conductor distinct; pimoid embolic process distally pointed, longer than embolus; embolus beginning at the 6:30 o’clock position; embolic tooth absent.

**Female (*paratype*)**: Total length 6.99. Carapace 3.31 long, 2.48 wide. Abdomen 3.68 long, 2.94 wide. Eye sizes and interdistances: AME 0.12, ALE 0.19, PME 0.19, PLE 0.18; AME-AME 0.11, AME-ALE 0.14, PME-PME 0.17, PME-PLE 0.21. Leg measurements: I: 18.88 (5.28, 6.35, 5.16, 2.09); II: 14.53 (5.06, 4.44, 3.28, 1.75); III: 13.31 (4.16, 4.18, 3.41, 1.56); IV: 17.08 (5.13, 5.89, 4.25, 1.81). Habitus as in Fig. 16F, G. Carapace yellowish; sternum brownish. Abdomen black with yellow chevrons. Legs brownish with black annulations. Epigyne (Fig. 16A–D): trapezoidal; ventral plate broad, longer than wide; dorsal plate nearly tongue-shaped; copulatory openings indistinct; spermathecae oval, separated by ca. 1/4 width of spermatheca; fertilization ducts brownish, laterally oriented.

##### Distribution.

Known only from the type locality, Tibet, China (Fig. 59).

#### Pimoa heishui

Taxon classificationAnimaliaAraneaePimoidae

Zhang & Li sp. nov.

7E330B94-90C3-57BB-989A-B8BE35F85C62

http://zoobank.org/D22099BA-306A-4372-9A40-79410BCEA25B

**Figure 17.**
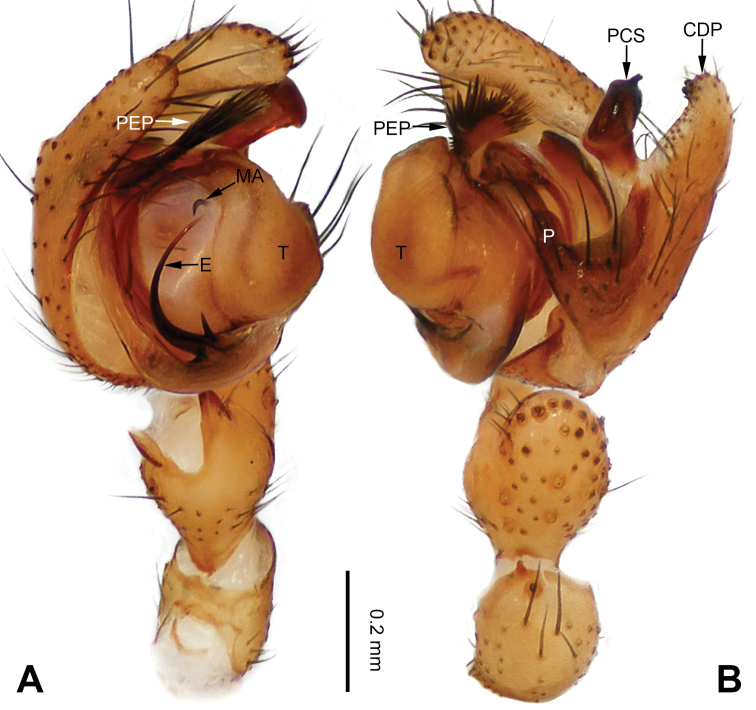
Left palp of *Pimoa heishui* sp. nov., holotype **A** prolateral view **B** retrolateral view. Abbreviations: CDP = cymbial denticulate process; E = embolus; MA = median apophysis; P = paracymbium; PCS = pimoid cymbial sclerite; PEP = pimoid embolic process; T = tegulum. Scale bar: equal for **A, B**.

**Figure 18.**
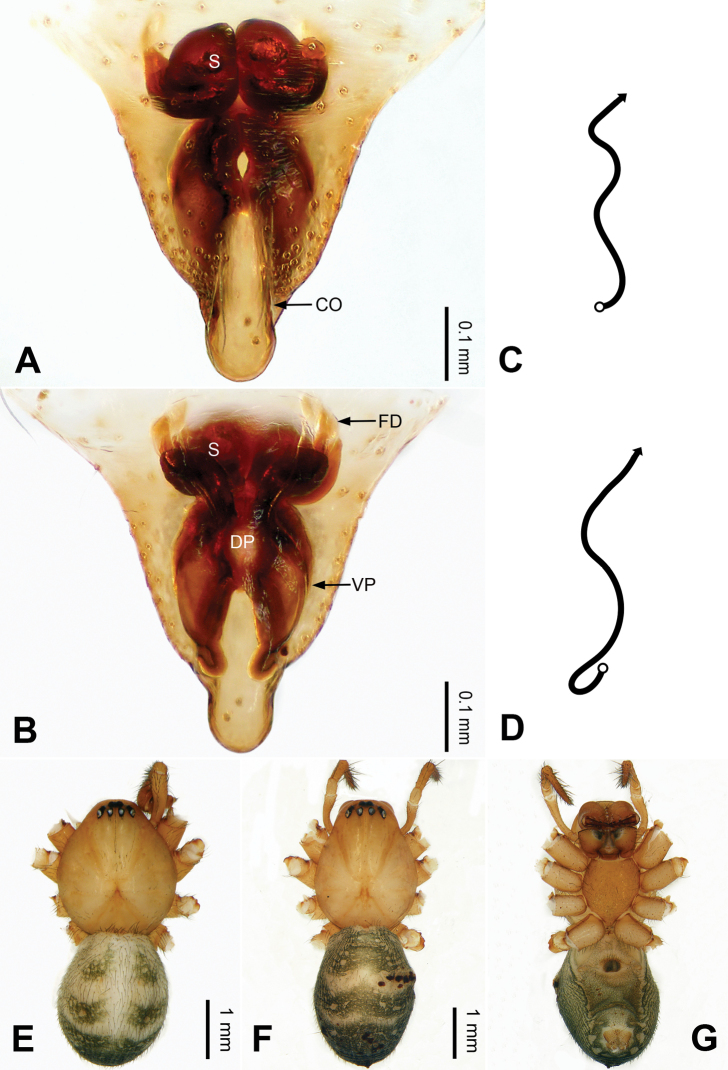
Epigyne and habitus of *Pimoa heishui* sp. nov., female paratype and male holotype **A** epigyne, ventral view **B** vulva, dorsal view **C** schematic course of internal duct system, ventral view **D** schematic course of internal duct system, dorsal view **E** male habitus, dorsal view **F** female habitus, dorsal view **G** female habitus, ventral view. Abbreviations: CO = copulatory opening; DP = dorsal plate of the epigyne; FD = fertilization duct; S = spermatheca; VP = ventral plate of epigyne. Scale bars: equal for **F, G**.

Figures 17 , 18 , 55 , 59

##### Type material.

***Holotype*:** ♂ (IZCAS-Ar41953), China, Sichuan, Heishui County, Deshiwo Village, the cave in the first grade power station, 32.03°N, 102.95°E, ca. 2429 m, 18.XI.2019, Z. Chen leg. ***Paratypes***: 1♂2♀ (IZCAS-Ar41954-Ar41956), same data as holotype.

##### Etymology.

The specific name is a noun in apposition taken from the type locality.

##### Diagnosis.

**Figure 19.**
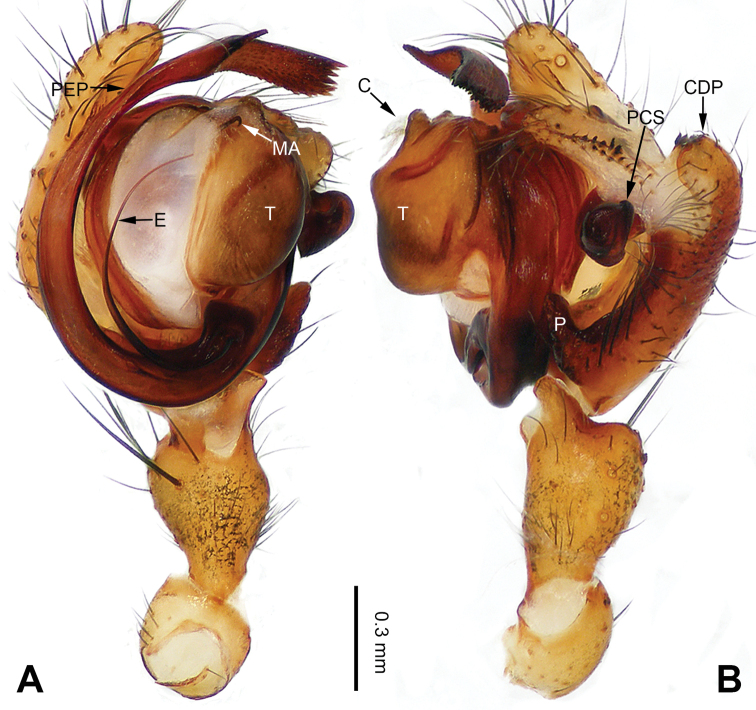
Left palp of *Pimoa jinchuan* sp. nov., holotype **A** prolateral view **B** retrolateral view. Abbreviations: C = conductor; CDP = cymbial denticulate process; E = embolus; MA = median apophysis; P = paracymbium; PCS = pimoid cymbial sclerite; PEP = pimoid embolic process; T = tegulum. Scale bar: equal for **A, B**.

The male of *Pimoa heishui* sp. nov. resembles those of *P. jinchuan* sp. nov. (Figs 19A, B, 55C) and *P. yele* sp. nov. (Figs 49A, B, 58A) but can be distinguished by the distally broad pimoid embolic process with spines (Fig. 55B) (vs. distally with scales and a short, slender spine subdistally in *P. jinchuan* sp. nov. and broad, robust, distally bifurcate apex with scales in *P. yele* sp. nov.) and also from *P. jinchuan* sp. nov. by the embolus with a short, slender spine proximally (Fig. 55B) (vs. without a spine) and by the short and wide cymbial denticulate process (Fig. 55B) (vs. distally long and narrow). The female of *P. heishui* sp. nov. resembles those of *P. lihengae* (see Griswold et al. 1999: 91–97, figs 18–21) and *P. wanglangensis* (see Yuan et al. 2019: 27, fig. 22G, H) but can be distinguished by the dorsal plate with a distally blunt tip (Fig. 18B) (vs. without a distal tip in *P. lihengae* and *P. wanglangensis*) and also distinguished from *P. wanglangensis* by the unseparated spermathecae (Fig. 18A) (vs. spermathecae with small separation).

##### Description.

**Male (*holotype*)**: Total length 4.35. Carapace 2.09 long, 1.94 wide. Abdomen 2.26 long, 1.88 wide. Eye sizes and interdistances: AME 0.10, ALE 0.15, PME 0.09, PLE 0.12; AME-AME 0.06, AME-ALE 0.06, PME-PME 0.11, PME-PLE 0.11. Leg measurements: I: 14.45 (4.09, 5.17, 3.47, 1.72); II: 13.09 (3.59, 4.65, 3.38, 1.47); III: 10.03 (3.13, 3.28, 2.56, 1.06); IV: 13.18 (3.91, 4.32, 3.51, 1.44). Habitus as in Fig. 18E. Carapace yellowish; thoracic fovea and radial grooves distinct; sternum brownish. Abdomen brown with yellow transverse chevrons, nearly oval. Legs yellowish without annulations. Palp (Figs 17A, B, 55B): patella short, almost as long as tibial length; tibia short, ca. 1/2 of cymbial length, with several macrosetae and a dorsal process; paracymbium short, ca. 1/3 of cymbial length, finger-shaped; pimoid cymbial sclerite L-shaped, ca. 1/3 of cymbial length; cymbial denticulate process short and broad, with more than 9 cuspules; median apophysis slender; conductor indistinct; pimoid embolic process membranous, distally broad with spines, longer than embolus; embolus beginning at the 8:30 o’clock position, with a short, slender spine proximally; embolic tooth absent.

**Female (*paratype*)**: Total length 4.91. Carapace 2.19 long, 1.84 wide. Abdomen 2.72 long, 1.78 wide. Eye sizes and interdistances: AME 0.11, ALE 0.13, PME 0.12, PLE 0.13; AME-AME 0.04, AME-ALE 0.11, PME-PME 0.12, PME-PLE 0.12. Leg measurements: I: 13.44 (3.78, 4.75, 3.22, 1.69); II: 12.22 (3.69, 4.25, 2.94, 1.34); III: 9.55 (2.94, 3.14, 2.34, 1.13); IV: 12.08 (3.59, 4.15, 3.03, 1.31). Habitus as in Fig. 18F, G. Carapace yellowish; thoracic fovea and radial grooves distinct; sternum brownish. Abdomen brown with yellow transverse chevrons. Legs yellowish without annulations. Epigyne (Fig. 18A–D): subtriangular; ventral plate broad, width subequal to length; dorsal plate broad, with a distally blunt tip; copulatory openings distinct; spermathecae nearly triangular, unseparated; fertilization ducts yellowish, laterally oriented.

##### Distribution.

Known only from the type locality, Sichuan, China (Fig. 59).

#### Pimoa jinchuan

Taxon classificationAnimaliaAraneaePimoidae

Zhang & Li sp. nov.

5CDC0A82-E64E-58ED-AB9C-45C850DC7A47

http://zoobank.org/27C2FD57-5A94-45CA-B1D5-B3A599F2BA63

**Figure 20.**
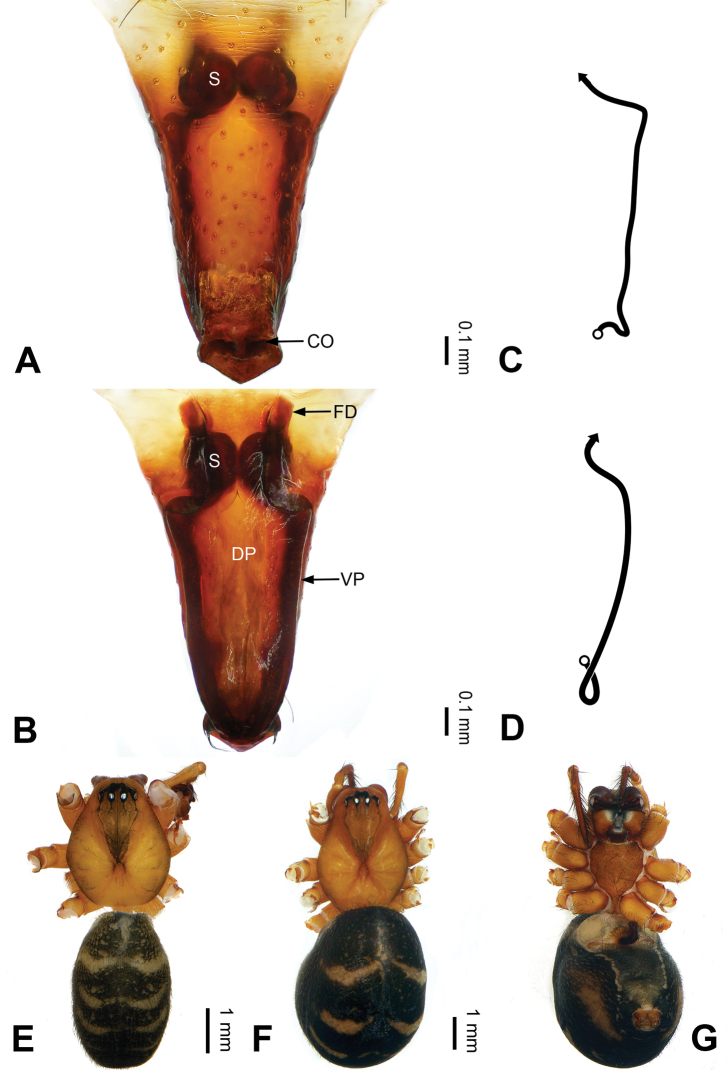
Epigyne and habitus of *Pimoa jinchuan* sp. nov., female paratype and male holotype **A** epigyne, ventral view **B** vulva, dorsal view **C** schematic course of internal duct system, ventral view **D** schematic course of internal duct system, dorsal view **E** male habitus, dorsal view **F** female habitus, dorsal view **G** female habitus, ventral view. Abbreviations: CO = copulatory opening; DP = dorsal plate of the epigyne; FD = fertilization duct; S = spermatheca; VP = ventral plate of epigyne. Scale bars: equal for **F, G**.

Figures 19 , 20 , 55 , 59

##### Type material.

***Holotype*:** ♂ (IZCAS-Ar41957), China, Sichuan, Jinchuan County, the bridge from Xilizhai Village to Xiaojin County, 31.34°N, 102.19°E, ca. 3411 m, 23.XI.2019, Z. Chen leg. ***Paratypes***: 1♂1♀ (IZCAS-Ar41958-Ar41959), same data as holotype.

##### Etymology.

The specific name is a noun in apposition taken from the type locality.

##### Diagnosis.

The male of *Pimoa jinchuan* sp. nov. resembles those of *P. lata* (see Zhang and Li 2019: 6, fig. 3A–C), *P. trifurcata* Xu & Li, 2007 (see Xu and Li 2007: 496, figs 48–54) and *P. yele* sp. nov. (Figs 49A, B, 58A) but can be distinguished by the pimoid embolic process with scales distally and a spine subdistally (Fig. 55C) (vs. distally with two jagged tips in *P. lata*, a distally trifurcate apex in *P. trifurcata* and *P. yele* sp. nov.) and also from *P. lata* by the finger-shaped paracymbium (Fig. 55C) (vs. with a blunt tip), from *P. trifurcata* by the embolus without a spine (Fig. 55C) (vs. with a short, slender spine proximally), and from *P. yele* sp. nov. by the broad cymbial denticulate process (Fig. 55C) (vs. short and distally narrow) and by the embolus without a spine (Fig. 55C) (vs. with a short, slender spine proximally). The female of *P. jinchuan* sp. nov. resembles those of *P. crispa* (see Hormiga 1994a: 63, figs 239–247) and *P. yele* sp. nov. (Fig. 50A–D) but can be distinguished by the tongue-shaped, distally curved ventral plate (Fig. 20A) (vs. triangular in *P. crispa* and broad in *P. yele* sp. nov.) and also distinguished from *P. crispa* by the unseparated spermathecae (Fig. 20A) (vs. separated by short distance) and from *P. yele* sp. nov. by the distally pointed dorsal plate (Fig. 20B) (vs. triangular with a distal tip).

##### Description.

**Male (*holotype*)**: Total length 5.69. Carapace 2.91 long, 2.34 wide. Abdomen 2.78 long, 2.16 wide. Eye sizes and interdistances: AME 0.14, ALE 0.17, PME 0.14, PLE 0.16; AME-AME 0.10, AME-ALE 0.11, PME-PME 0.15, PME-PLE 0.17. Leg measurements: I: 31.10 (8.53, 10.21, 9.45, 2.91); II: 26.32 (7.34, 8.33, 8.09, 2.56); III: 15.88 (4.84, 4.90, 4.48, 1.66); IV: 21.35 (6.44, 6.88, 6.06, 1.97). Habitus as in Fig. 20E. Carapace yellowish with black lateral margins; thoracic fovea and radial grooves distinct; sternum brownish. Abdomen black with yellowish transverse chevrons, nearly oval. Legs brownish without annulations. Palp (Figs 19A, B, 55C): patella short, ca. 1/2 of tibial length, with one retrolateral macroseta; tibia short, ca. 1/2 of cymbial length, with several macrosetae and a dorsal process; paracymbium short, ca. 1/4 of cymbial length, hook shaped; pimoid cymbial sclerite U-shaped, ca. 1/3 of cymbial length; cymbial denticulate process broad, with more than 6 cuspules; median apophysis slender; conductor distinct; pimoid embolic process broad, robust, with scales distally and a spine subdistally, longer than embolus; embolus beginning at the 6:00 o’clock position; embolic tooth absent.

**Female (*paratype*)**: Total length 8.76. Carapace 3.88 long, 2.81 wide. Abdomen 4.88 long, 3.76 wide. Eye sizes and interdistances: AME 0.17, ALE 0.18, PME 0.17, PLE 0.19; AME-AME 0.11, AME-ALE 0.17, PME-PME 0.21, PME-PLE 0.20. Leg measurements: I: 28.81 (8.06, 9.78, 8.19, 2.78); II: 24.27 (7.06, 8.37, 6.75, 2.09); III: 17.69 (5.16, 5.72, 4.97, 1.84); IV: 22.56 (6.78, 7.72, 6.22, 1.84). Habitus as in Fig. 20F, G. Carapace yellowish with black lateral margins; thoracic fovea and radial grooves distinct; sternum brownish. Abdomen black with yellowish transverse chevrons. Legs brownish without annulations. Epigyne (Fig. 20A–D): bullet-shaped; ventral plate tongue shaped, distally curved, width ca. 1/2 of length; dorsal plate subtriangular, distally pointed; copulatory openings distinct; spermathecae oval, unseparated; fertilization ducts yellowish, laterally oriented.

##### Distribution.

Known only from the type locality, Sichuan, China (Fig. 59).

#### Pimoa khaptad

Taxon classificationAnimaliaAraneaePimoidae

Zhang & Li sp. nov.

293FCB27-8B33-571B-8F4A-3B850C53799E

http://zoobank.org/9D438740-8CD2-4E61-BCB3-22A6DE74293B

Figures 21 , 59

##### Type material.

***Holotype*:** ♀ (IZCAS-Ar41960), Nepal, Karnali District, Khaptad National Park 29.34°N, 81.05°E, ca. 2284 m, 18.IV.2019, C. Shrestha leg. ***Paratype***: 1♀ (IZCAS-Ar41961), same data as holotype.

##### Etymology.

The specific name is a noun in apposition taken from the type locality.

##### Diagnosis.

**Figure 21.**
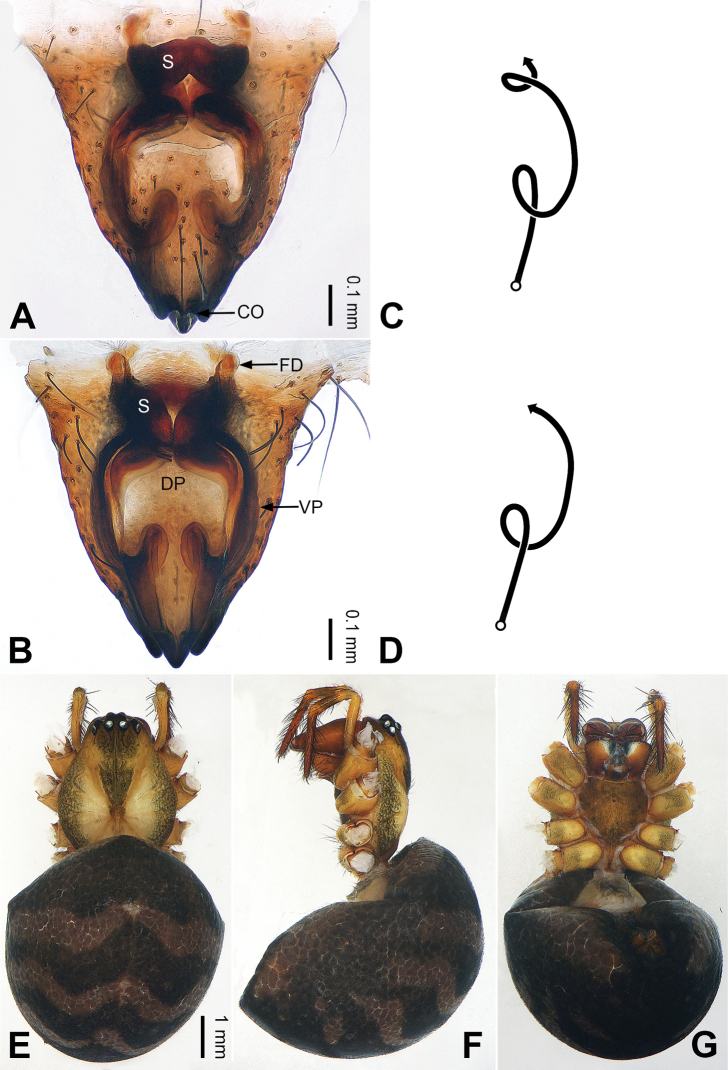
Epigyne and habitus of *Pimoa khaptad* sp. nov., female holotype **A** epigyne, ventral view **B** schematic course of internal duct system, ventral view **C** vulva, dorsal view **D** schematic course of internal duct system, dorsal view **E** female habitus, dorsal view **F** female habitus, lateral view **G** female habitus, ventral view. Abbreviations: CO = copulatory opening; DP = dorsal plate of the epigyne; FD = fertilization duct; S = spermatheca; VP = ventral plate of epigyne. Scale bars: equal for **E–G**.

*Pimoa khaptad* sp. nov. resembles those of *P. rara* sp. nov. (Fig. 40A–D) and *P. samyai* Zhang & Li, 2020 (see Zhang et al. 2020: 97, fig. 13A–D) but can be distinguished by the nearly bean-shaped, unseparated spermathecae (Fig. 21A) (vs. oval, close to each other in *P. rara* sp. nov. and nearly oval, separated by ca. 1/2 width of a spermatheca in *P. samyai*) and also distinguished from *P. samyai* by the pointed dorsal plate (Fig. 21B) (vs. blunt).

##### Description.

**Female (*holotype*)**: Total length 8.31. Carapace 2.72 long, 2.84 wide. Abdomen 5.59 long, 5.36 wide. Eye sizes and interdistances: AME 0.19, ALE 0.17, PME 0.22, PLE 0.22; AME-AME 0.12, AME-ALE 0.18, PME-PME 0.16, PME-PLE 0.20. Leg measurements: I: 27.96 (7.91, 9.09, 7.90, 3.06); II: – (6.69, –, –, –); III: 15.55 (4.81, 4.88, 4.34, 1.52); IV: – (5.01, 6.75, –, –). Habitus as in Fig. 21E–G. Carapace yellowish; thoracic fovea and radial grooves distinct; sternum brownish. Abdomen black with yellowish transverse bands, nearly oval. Legs brownish with black annulations. Epigyne (Fig. 21A–D): triangular; ventral plate broad, length subequal to width; dorsal plate tongue shaped, distally pointed; copulatory openings distinct; spermathecae nearly bean-shaped, unseparated; fertilization ducts yellow, anteriorly oriented.

**Male**: Unknown.

##### Distribution.

Known only from the type locality, Karnali District, Nepal (Fig. 59).

#### Pimoa koshi

Taxon classificationAnimaliaAraneaePimoidae

Zhang & Li sp. nov.

359DD7DB-C00B-5430-B4F9-BF430B01F810

http://zoobank.org/DA50ABC8-E015-4E7A-995A-7FDBCEB2AE2C

Figures 22 , 59

##### Type material.

***Holotype*:** ♀ (IZCAS-Ar41962), Nepal, Koshi District, Tamaphok, 27.16°N, 87.41°E, ca. 2495 m, 31.III.2019, C. Shrestha leg. ***Paratype***: 1♀ (IZCAS-Ar41963), same data as holotype.

##### Etymology.

The specific name is a noun in apposition taken from the type locality.

##### Diagnosis.

**Figure 22.**
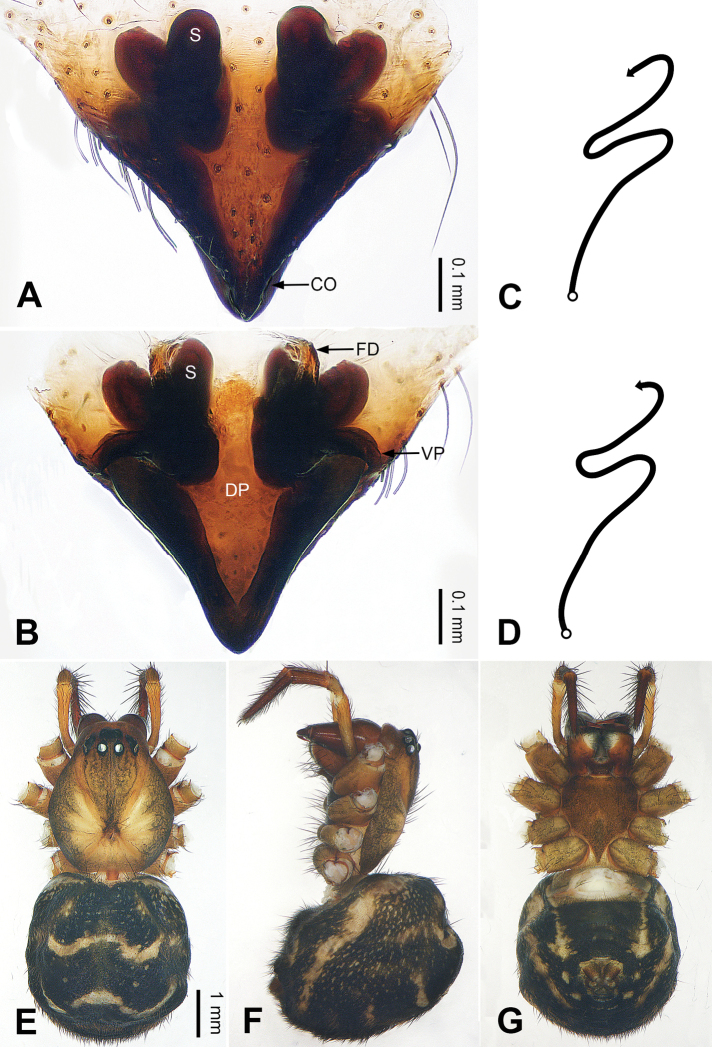
Epigyne and habitus of *Pimoa koshi* sp. nov., female holotype **A** epigyne, ventral view **B** schematic course of internal duct system, ventral view **C** vulva, dorsal view **D** schematic course of internal duct system, dorsal view **E** female habitus, dorsal view **F** female habitus, lateral view **G** female habitus, ventral view. Abbreviations: CO = copulatory opening; DP = dorsal plate of the epigyne; FD = fertilization duct; S = spermatheca; VP = ventral plate of epigyne. Scale bars: equal for **E–G**.

*Pimoa koshi* sp. nov. resembles those of *P. nyalam* sp. nov. (Fig. 36A–D) and *P. nyingchi* (see Zhang et al. 2020: 91, fig. 9A–D) but can be distinguished by the spermathecae separated by the width of a spermatheca (Fig. 22A) (vs. separated by ca. 1/3 width of a spermatheca in *P. nyalam* sp. nov. and unseparated in *P. nyingchi*) and also distinguished from *P. nyingchi* by the anteriorly oriented fertilization ducts (Fig. 22B) (vs. laterally oriented) and from *P. nyalam* sp. nov. by the pointed dorsal plate (Fig. 22B) (vs. blunt).

##### Description.

**Female (*holotype*)**: Total length 6.51. Carapace 3.13 long, 2.56 wide. Abdomen 3.38 long, 3.36 wide. Eye sizes and interdistances: AME 0.17, ALE 0.19, PME 0.18, PLE 0.19; AME-AME 0.10, AME-ALE 0.15, PME-PME 0.16, PME-PLE 0.22. Leg measurements: I: 18.88 (5.22, 6.34, 4.94, 2.38); II: 16.22 (4.72, 5.28, 4.22, 2.00); III: 11.29 (3.48, 3.34, 3.13, 1.34); IV: 15.02 (4.70, 4.72, 3.91, 1.69). Habitus as in Fig. 22E–G. Carapace yellowish; thoracic fovea and radial grooves distinct; sternum yellow. Abdomen black with yellowish transverse bands, nearly oval. Legs brownish with distinct black annulations. Epigyne (Fig. 22A–D): triangular; ventral and dorsal plates broad, length subequal to width; copulatory openings distinct; spermathecae oval, separated by the width of a spermatheca; fertilization ducts yellow, anteriorly oriented.

**Male**: Unknown.

##### Distribution.

Known only from the type locality, Koshi District, Nepal (Fig. 59).

#### Pimoa lhatog

Taxon classificationAnimaliaAraneaePimoidae

Zhang & Li sp. nov.

E28E7F0B-4B4C-5354-837A-BABF55328805

http://zoobank.org/BE5D5F7B-3DAE-464C-84B5-CC68A4B2455A

Figures 23 , 59

##### Type material.

***Holotype*:** ♀ (IZCAS-Ar41964), China, Tibet, Nyingchi, Nang County, Lhatog Town, Lhatog Lake, 28.75°N, 93.08°E, ca. 4122 m, 5.X.2020, Z. Chen leg. ***Paratype***: 1♀ (IZCAS-Ar41965), same data as holotype.

##### Etymology.

The specific name is a noun in apposition taken from the type locality.

##### Diagnosis.

**Figure 23.**
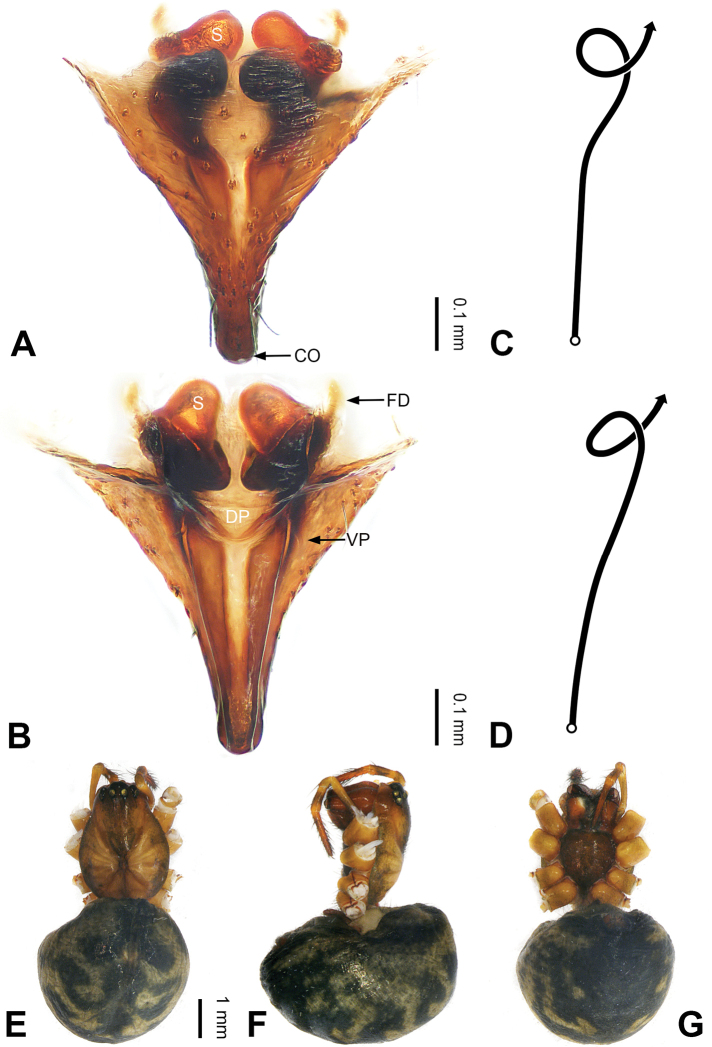
Epigyne and habitus of *Pimoa lhatog* sp. nov., female holotype **A** epigyne, ventral view **B** schematic course of internal duct system, ventral view **C** vulva, dorsal view **D** schematic course of internal duct system, dorsal view **E** female habitus, dorsal view **F** female habitus, lateral view **G** female habitus, ventral view. Abbreviations: CO = copulatory opening; DP = dorsal plate of the epigyne; FD = fertilization duct; S = spermatheca; VP = ventral plate of epigyne. Scale bars: equal for **E–G**.

*Pimoa lhatog* sp. nov. resembles those of *P. crispa* (see Hormiga 1994a: 63, figs 239–247) and *P. mainling* (see Zhang et al. 2020: 89, fig. 7A–D) but can be distinguished from *P. crispa* by the funnel-shaped epigyne (Fig. 23A) (vs. triangular) and from *P. mainling* by the bean-shaped spermathecae (Fig. 23A) (vs. nearly oval).

##### Description.

**Female (*holotype*)**: Total length 7.18. Carapace 2.66 long, 2.34 wide. Abdomen 4.52 long, 3.84 wide. Eye sizes and interdistances: AME 0.19, ALE 0.17, PME 0.15, PLE 0.16; AME-AME 0.05, AME-ALE 0.13, PME-PME 0.15, PME-PLE 0.14. Leg measurements: I: 17.56 (4.88, 6.06, 4.41, 2.21); II: 15.01 (4.38, 5.01, 3.78, 1.84); III: 10.79 (3.25, 3.31, 2.84, 1.39); IV: 13.75 (4.19, 4.63, 3.34, 1.59). Habitus as in Fig. 23E–G. Carapace yellowish with black lateral margins; thoracic fovea and radial grooves distinct; sternum brownish. Abdomen black with brownish, transverse chevrons. Legs brownish with black annulations. Epigyne (Fig. 23A–D): funnel-shaped; ventral plate distally narrow, length subequal to width; dorsal plates narrow, width ca. 1/2 length; copulatory openings distinct; spermathecae bean-shaped, separated by ca. 1/3 width of a spermatheca; fertilization ducts yellow, laterally oriented.

**Male**: Unknown.

##### Distribution.

Known only from the type locality, Tibet, China (Fig. 59).

#### Pimoa mechi

Taxon classificationAnimaliaAraneaePimoidae

Zhang & Li sp. nov.

396A877B-054F-579E-A794-6AAACCE876F3

http://zoobank.org/1C976A2A-967C-4069-A93E-7763E89DAC39

**Figure 24.**
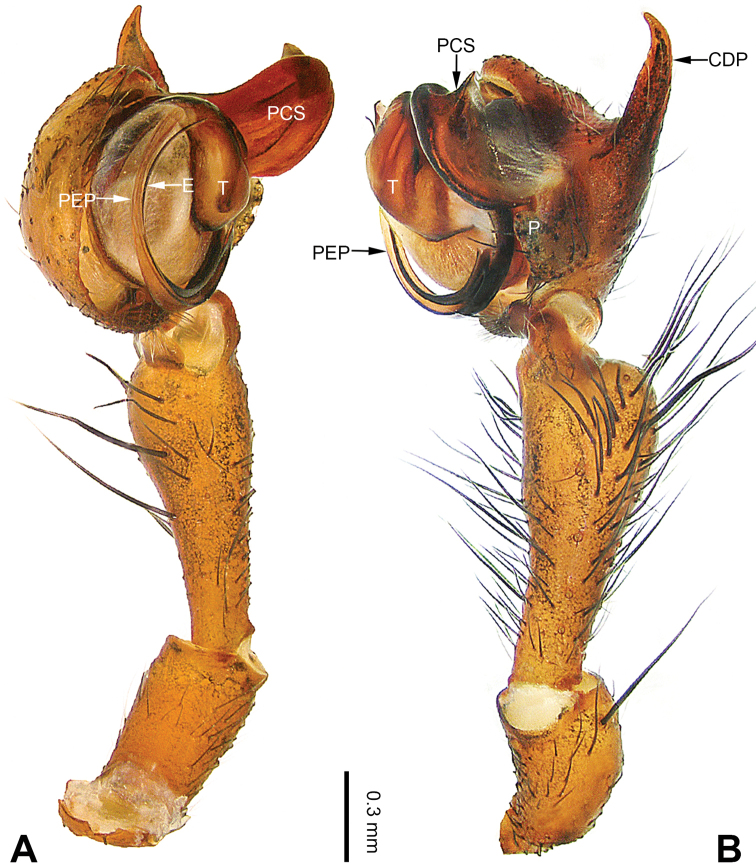
Left palp of *Pimoa mechi* sp. nov., holotype **A** prolateral view **B** retrolateral view. Abbreviations: CDP = cymbial denticulate process; E = embolus; P = paracymbium; PCS = pimoid cymbial sclerite; PEP = pimoid embolic process; T = tegulum. Scale bar: equal for **A, B**.

**Figure 25.**
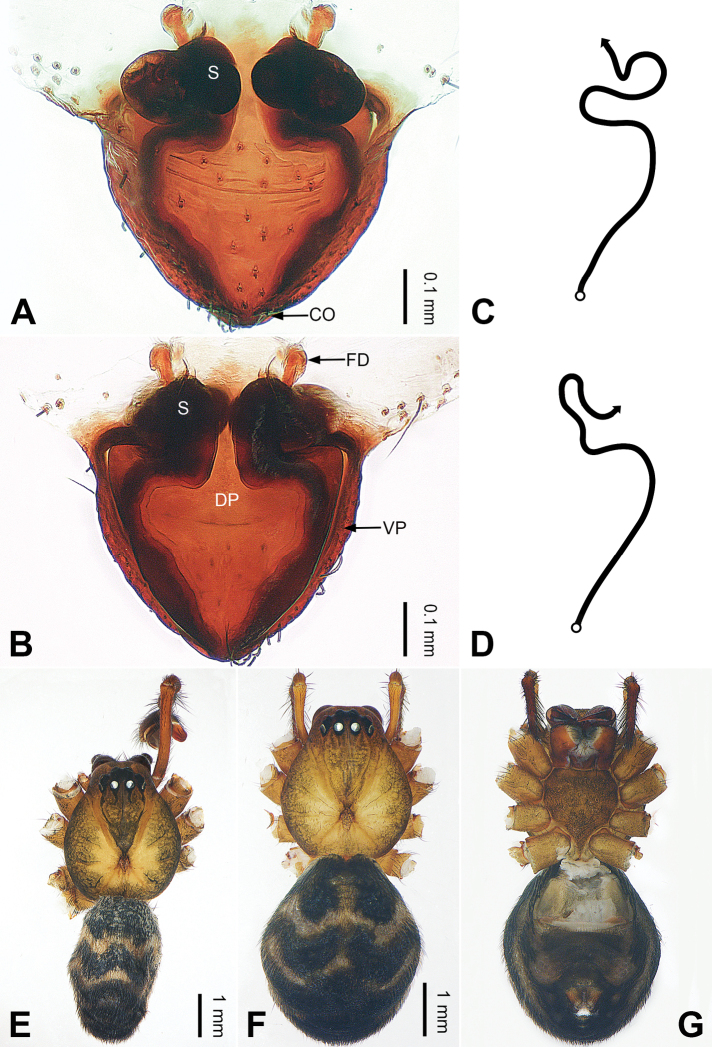
Epigyne and habitus of *Pimoa mechi* sp. nov., female paratype and male holotype **A** epigyne, ventral view **B** vulva, dorsal view **C** schematic course of internal duct system, ventral view **D** schematic course of internal duct system, dorsal view **E** male habitus, dorsal view **F** female habitus, dorsal view **G** female habitus, ventral view. Abbreviations: CO = copulatory opening; DP = dorsal plate of the epigyne; FD = fertilization duct; S = spermatheca; VP = ventral plate of epigyne. Scale bars: equal for **F, G**.

Figures 24 , 25 , 55 , 59

##### Type material.

***Holotype*:** ♂ (IZCAS-Ar41966), Nepal, Mechi District, Taplejung, Rhododendron, 27.37°N, 87.73°E, ca. 2720 m, 3.IV.2019, C. Shrestha leg. ***Paratype***: 1♀ (IZCAS-Ar41967), same data as holotype; 1♀ (IZCAS-Ar41968), Nepal, Mechi District, Taplejung, 27.35°N, 87.70°E , ca. 2452 m, 2.IV.2019, C. Shrestha leg.

##### Etymology.

The specific name is a noun in apposition taken from the type locality.

##### Diagnosis.

The male of *Pimoa mechi* sp. nov. resembles *P. yadong* Zhang & Li, 2020 (see Zhang et al. 2020: 99, fig. 14A–C) but can be distinguished by the large pimoid cymbial sclerite (Fig. 55D) (vs. smaller) and by the long cymbial denticulate process (Fig. 55D) (vs. short). The female of *P. mechi* sp. nov. resembles those of *P. crispa* (see Hormiga 1994a: 63, figs 239–247) and *P. samyai* (see Zhang et al. 2020: 97, fig. 13A–D) but can be distinguished from *P. crispa* by the broad dorsal plate (Fig. 25B) (vs. narrow) and from *P. samyai* by the spermathecae separated by a short distance (Fig. 25A) (vs. separated by ca. 1/2 width of a spermatheca) and also by the distally pointed dorsal plate (Fig. 25B) (vs. blunt).

##### Description.

**Male (*holotype*)**: Total length 6.31. Carapace 3.03 long, 2.63 wide. Abdomen 3.28 long, 1.97 wide. Eye sizes and interdistances: AME 0.16, ALE 0.20, PME 0.19, PLE 0.19; AME-AME 0.14, AME-ALE 0.10, PME-PME 0.18, PME-PLE 0.17. Leg measurements: I: 22.70 (5.91, 7.38, 6.53, 2.88); II: 16.25 (5.16, 4.66, 4.34, 2.09); III: 14.75 (4.34, 4.44, 4.28, 1.69); IV: 19.28 (5.69, 6.06, 5.44, 2.09). Habitus as in Fig. 25E. Carapace yellowish with black lateral margins; thoracic fovea and radial grooves distinct; sternum brownish. Abdomen black with yellowish transverse chevrons. Legs yellowish with black annulations. Palp (Figs 24A, B, 55D): patella short, ca. 1/2 of tibial length, with one retrolateral macroseta; tibia long, ca. 1.5 times of cymbial length, with several macrosetae and a dorsal process; paracymbium short, ca. 1/3 of cymbial length, hook shaped; pimoid cymbial sclerite large, nearly L-shaped, ca. 1/2 of cymbial length; cymbial denticulate process long and distally pointed, with more than 6 cuspules; median apophysis indistinct; conductor distinct; pimoid embolic process distally blunt, length subequal to embolus; embolus beginning at 5:00 o’clock position; embolic tooth absent.

**Female (*paratype*)**: Total length 6.38. Carapace 2.66 long, 2.38 wide. Abdomen 3.72 long, 5.91 wide. Eye sizes and interdistances: AME 0.18, ALE 0.19, PME 0.18, PLE 0.16; AME-AME 0.09, AME-ALE 0.10, PME-PME 0.14, PME-PLE 0.14. Leg measurements: I: 19.48 (5.26, 6.09, 5.70, 2.43); II: 15.49 (4.28, 5.13, 4.17, 1.91); III: 10.35 (3.13, 3.06, 2.78, 1.38); IV: 14.28 (4.56, 4.66, 3.53, 1.53). Habitus as in Fig. 25F, G. Carapace yellowish; thoracic fovea and radial grooves distinct; sternum brownish. Abdomen black with yellowish transverse bands, nearly oval. Legs brownish with black annulations. Epigyne (Fig. 25A–D): subtriangular; ventral and dorsal plates broad, length subequal to width; copulatory openings distinct; spermathecae oval, separated by short distance; fertilization ducts yellow, laterally oriented.

##### Distribution.

Known only from the type locality, Mechi District, Nepal (Fig. 59).

#### Pimoa miandam

Taxon classificationAnimaliaAraneaePimoidae

Zhang & Li sp. nov.

762E43EB-880B-5239-A287-AE1A3DB31744

http://zoobank.org/7EE84081-4BB5-4358-A1E8-E05C4BE9E310

**Figure 26.**
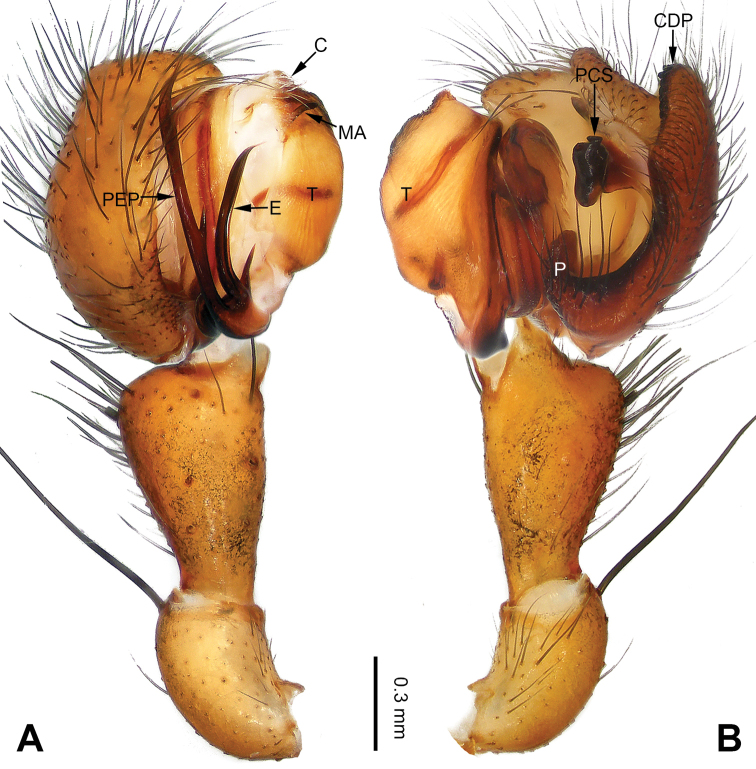
Left palp of *Pimoa miandam* sp. nov., holotype **A** prolateral view **B** retrolateral view. Abbreviations: C = conductor; CDP = cymbial denticulate process; E = embolus; MA = median apophysis; P = paracymbium; PCS = pimoid cymbial sclerite; PEP = pimoid embolic process; T = tegulum. Scale bar: equal for **A, B**.

**Figure 27.**
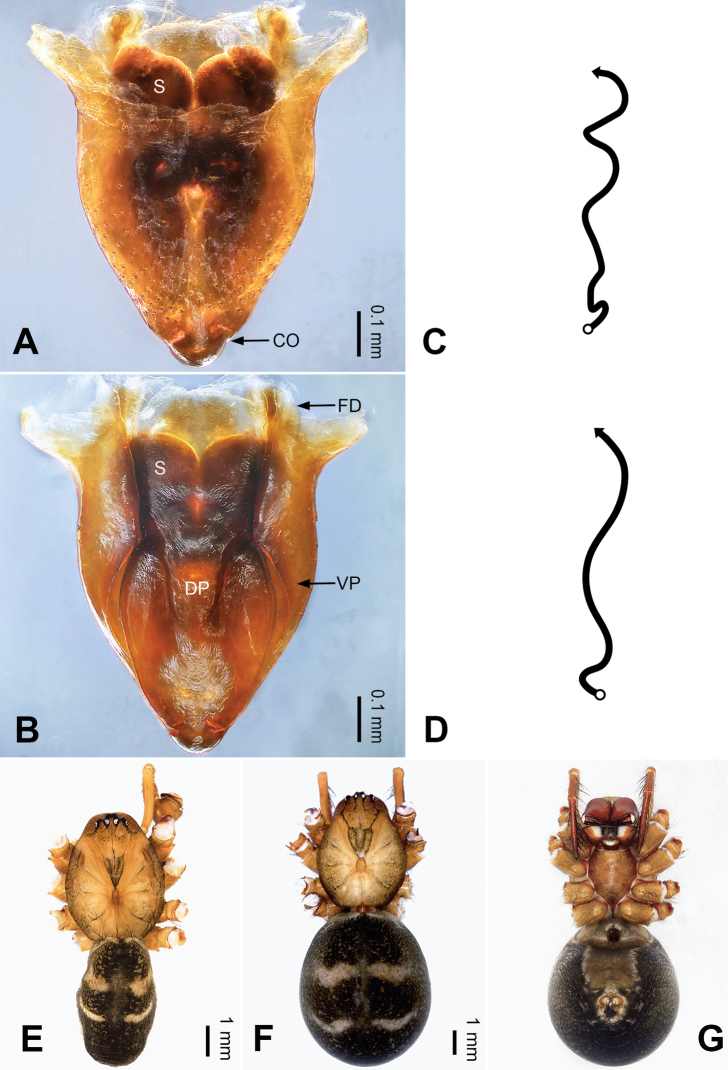
Epigyne and habitus of *Pimoa miandam* sp. nov., female paratype and male holotype **A** epigyne, ventral view **B** vulva, dorsal view **C** schematic course of internal duct system, ventral view **D** schematic course of internal duct system, dorsal view **E** male habitus, dorsal view **F** female habitus, dorsal view **G** female habitus, ventral view. Abbreviations: CO = copulatory opening; DP = dorsal plate of the epigyne; FD = fertilization duct; S = spermatheca; VP = ventral plate of epigyne. Scale bars: equal for **F, G**.

Figures 26 , 27 , 56 , 59

##### Type material.

***Holotype*:** ♂ (IZCAS-Ar41969), Pakistan, Khyber Pakhtunkhwa, Swat, Miandam, near river, 35.03°N, 72.57°E, ca. 2242 m, 31.V.2019, A. Ali leg. ***Paratypes***: 1♂2♀ (IZCAS-Ar41970-Ar41972), same data as holotype.

##### Etymology.

The specific name is a noun in apposition taken from the type locality.

##### Diagnosis.

The male of *Pimoa miandam* sp. nov. resembles those of *P. duiba* Zhang & Li, 2020 (see Zhang et al. 2020: 84, fig. 3A–C) and *P. rongxar* (see Zhang et al. 2020: 94, fig. 10A–C) but can be distinguished from *P. duiba* by the pimoid embolic process with a slender and nearly medial spine (Fig. 56A) (vs. without a spine) and by the embolus beginning at the 8:00 o’clock position (Fig. 56A) (vs. 7:00), and distinguished from *P. rongxar* by the V-shaped, distally curved pimoid cymbial sclerite, ca. 1/3 of cymbial length (Fig. 56A) (vs. large, long, and subdistally wide pimoid cymbial sclerite). The female of *P. miandam* sp. nov. also resembles those of *P. duiba* (see Zhang et al. 2020: 84, fig. 4A–D) and *P. rongxar* (see Zhang et al. 2020: 94, fig. 11A–D) but can be distinguished from *P. duiba* by the unseparated spermathecae (Fig. 27A) (vs. a short distance between the spermathecae) and from *P. rongxar* by the tongue shaped ventral plate (Fig. 27A) (vs. triangular).

##### Description.

**Male (*holotype*)**: Total length 8.24. Carapace 4.41 long, 3.25 wide. Abdomen 3.83 long, 2.69 wide. Eye sizes and interdistances: AME 0.17, ALE 0.21, PME 0.20, PLE 0.19; AME-AME 0.04, AME-ALE 0.17, PME-PME 0.15, PME-PLE 0.24. Leg measurements: I: 47.14 (12.69, 15.38, 15.13, 3.94); II: 37.78 (11.53, 11.31, 11.50, 3.44); III: 31.98 (9.55, 10.09, 10.03, 2.31); IV: – (11.63, 12.38, 11.13, –). Habitus as in Fig. 27E. Carapace yellowish, with black lateral margins; thoracic fovea and radial grooves distinct; sternum brownish. Abdomen black with yellow transverse bands, nearly oval. Legs brownish without annulations. Palp (Figs 26A, B, 56A): patella short, ca. 1/2 of tibial length; tibia long, ca. 1/2 of cymbial length; paracymbium short, ca. 1/3 of cymbial length, hook shaped; pimoid cymbial sclerite V-shaped, distally curved, ca. 1/3 of cymbial length; cymbial denticulate process short and distally blunt, with more than 9 cuspules; median apophysis slender; conductor distinct; pimoid embolic process distally pointed, longer than embolus, with a short slender, nearly medial spine; embolus beginning at the 8:00 o’clock position, with a short, slender spine proximally; embolic tooth absent.

**Female (*paratype*)**: Total length 11.59. Carapace 4.81 long, 3.78 wide. Abdomen 6.78 long, 6.07 wide. Eye sizes and interdistances: AME 0.15, ALE 0.18, PME 0.16, PLE 0.18; AME-AME 0.13, AME-ALE 0.17, PME-PME 0.16, PME-PLE 0.29. Leg measurements: I: 42.97 (11.81, 14.09, 12.94, 4.13); II: 38.20 (10.63, 12.54, 11.44, 3.59); III: 28.51 (8.63, 9.19, 8.44, 2.25); IV: 34.12 (10.68, 11.19, 9.81, 2.44). Habitus as in Fig. 27F, G. Carapace yellowish with black lateral margins; sternum brownish. Abdomen black with yellowish transverse bands. Legs brownish without annulations. Epigyne (Fig. 27A–D): tongue-shaped; ventral and dorsal plate broad, longer than wide; copulatory openings distinct; spermathecae nearly round, unseparated; fertilization ducts yellowish, laterally oriented.

##### Distribution.

Known only from the type locality, Khyber Pakhtunkhwa, Pakistan (Fig. 59).

#### Pimoa miero

Taxon classificationAnimaliaAraneaePimoidae

Zhang & Li sp. nov.

F8AF1951-6949-59EA-951B-E425279F2EAC

http://zoobank.org/A28D23A7-A140-4EF9-92DC-7C3C23B951F9

**Figure 28.**
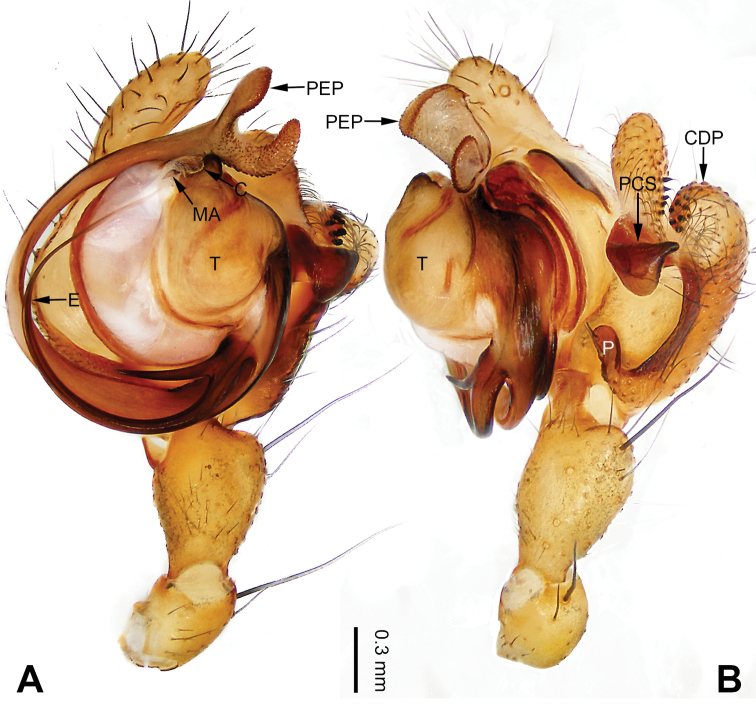
Left palp of *Pimoa miero* sp. nov., holotype **A** prolateral view **B** retrolateral view. Abbreviations: C = conductor; CDP = cymbial denticulate process; E = embolus; MA = median apophysis; P = paracymbium; PCS = pimoid cymbial sclerite; PEP = pimoid embolic process; T = tegulum. Scale bar: equal for **A, B**.

**Figure 29.**
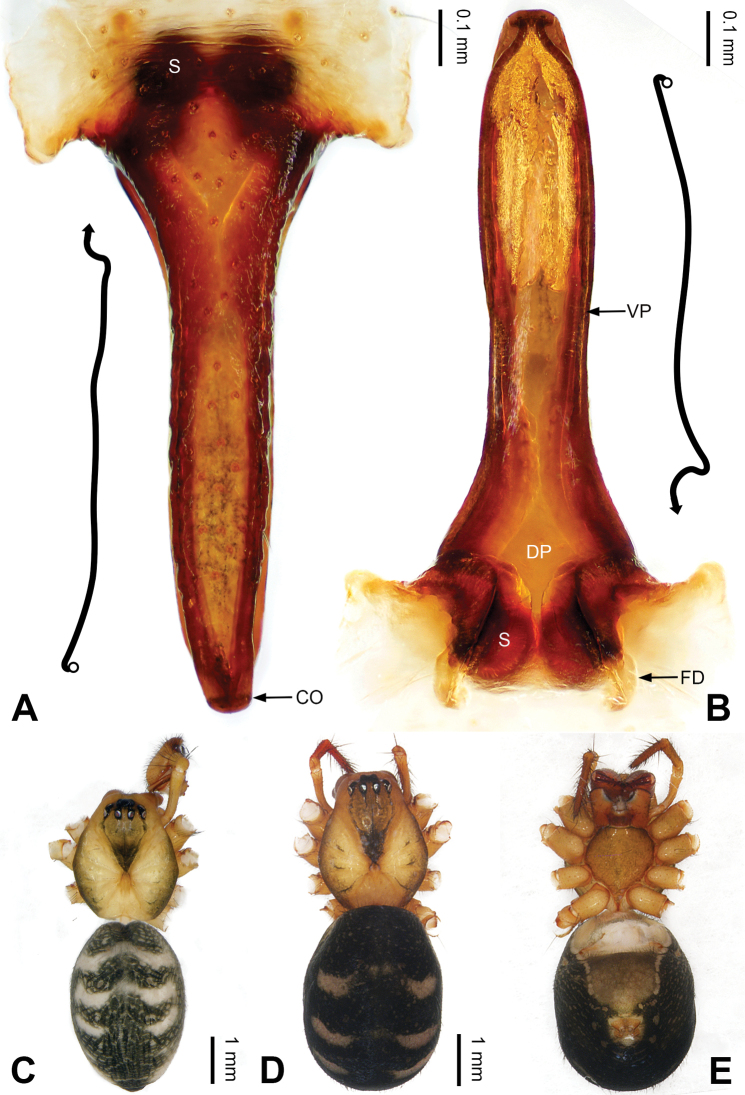
Epigyne and habitus of *Pimoa miero* sp. nov., female paratype and male holotype **A** epigyne, ventral view **B** vulva, dorsal view **C** male habitus, dorsal view **D** female habitus, dorsal view **E** female habitus, ventral view. Abbreviations: CO = copulatory opening; DP = dorsal plate of the epigyne; FD = fertilization duct; S = spermatheca; VP = ventral plate of epigyne. Scale bars: equal for **D, E**.

Figures 28 , 29 , 56 , 59

##### Type material.

***Holotype*:** ♂ (IZCAS-Ar41973), China, Sichuan, Li County, Miero Town, Siboguo Village, 31.67°N, 102.72°E, ca. 3029 m, 21.XI.2019, Z. Chen leg. ***Paratypes***: 1♂2♀ (IZCAS-Ar41974-Ar41976), China, Sichuan, Gakog County, Sajinse Town, on the way from Mt. Zhegu to Shuama Crossing, 31.93°N, 102.65°E, ca. 3458 m, 20.XI.2019, Z. Chen leg.

##### Etymology.

The specific name is a noun in apposition taken from the type locality.

##### Diagnosis.

The male of *Pimoa miero* sp. nov. resembles those of *P. jinchuan* sp. nov. (Figs 19A, B, 55C), *P. trifurcata* (see Xu and Li 2007: 496, figs 48–54), and *P. yele* sp. nov. (Figs 49A, B, 58A) but can be distinguished by the short and broad cymbial denticulate process (Fig. 56B) (vs. narrow and longer in *P. jinchuan* sp. nov. and *P. trifurcata* and distally pointed in *P. yele* sp. nov.), and it can also be distinguished from *P. jinchuan* sp. nov. by a slender spine proximally on the embolus (Fig. 56B) (vs. without a spine), from *P. trifurcata* by the distally bifurcate apex of the pimoid embolic process (Fig. 56B) (vs. trifurcate apex), and from *P. yele* sp. nov. by the embolus beginning at the 5:00 o’clock position (Fig. 56B) (vs. 7:00). The female of *P. miero* sp. nov. can be distinguished from other congeners except for *P. danba* sp. nov. (Fig. 7A–D) by the length of dorsal plate significantly longer than width (Fig. 29A, B) (vs. length subequal to width), and from *P. danba* sp. nov. by the posterior part of epigyne with two tips (Fig. 29A) (vs. without tip).

##### Description.

**Male (*holotype*)**: Total length 5.25. Carapace 2.47 long, 2.27 wide. Abdomen 2.78 long, 2.44 wide. Eye sizes and interdistances: AME 0.12, ALE 0.14, PME 0.13, PLE 0.15; AME-AME 0.11, AME-ALE 0.13, PME-PME 0.12, PME-PLE 0.16. Leg measurements: I: 28.27 (7.76, 9.57, 8.56, 2.38); II: 22.67 (6.26, 7.84, 6.44, 2.13); III: 15.06 (4.52, 4.76, 4.31, 1.47); IV: 19.01 (5.88, 6.12, 5.38, 1.63). Habitus as in Fig. 29C. Carapace yellowish with black lateral margins; thoracic fovea and radial grooves distinct; sternum brownish. Abdomen grayish with yellow transverse chevrons, nearly oval. Legs brownish without annulations. Palp (Figs 28A, B, 56B): patella short, ca. 1/2 tibial length, with one retrolateral macroseta; tibia short, ca. 1/2 of cymbial length, with several macrosetae and a dorsal process; paracymbium short, ca. 1/4 of cymbial length, finger-shaped; pimoid cymbial sclerite V-shaped, ca. 1/3 of cymbial length; cymbial denticulate process short and broad, with more than 10 cuspules; median apophysis slender; conductor distinct; pimoid embolic process broad, robust, with distally bifurcate apex with scales, longer than embolus; embolus beginning at 5:00 o’clock position, with a short, slender spine proximally; embolic tooth absent.

**Female (*paratype*)**: Total length 6.10. Carapace 2.47 long, 2.13 wide. Abdomen 3.63 long, 2.78 wide. Eye sizes and interdistances: AME 0.17, ALE 0.17, PME 0.13, PLE 0.16; AME-AME 0.09, AME-ALE 0.11, PME-PME 0.14, PME-PLE 0.15. Leg measurements: I: 16.18 (4.53, 5.68, 4.28, 1.69); II: 14.11 (3.88, 4.79, 3.78, 1.66); III: 10.90 (3.28, 3.50, 2.84, 1.28); IV: 13.97 (4.19, 4.72, 3.59, 1.47). Habitus as in Fig. 29D, E. Carapace yellowish with black lateral margins; thoracic fovea and radial grooves distinct; sternum brownish. Abdomen black with yellowish transverse bands. Legs brownish without annulations. Epigyne (Fig. 29A, B): rocket-shaped; ventral and dorsal plate narrow, width ca. 1/3 of length; copulatory openings distinct; spermathecae oval, unseparated; fertilization ducts yellowish, laterally oriented.

##### Distribution.

Known only from the type locality, Sichuan, China (Fig. 59).

#### Pimoa mude

Taxon classificationAnimaliaAraneaePimoidae

Zhang & Li sp. nov.

84E738BD-D44D-5141-AF95-1ACDA887EFB6

http://zoobank.org/D9DC28E2-CF7D-4DFC-B38B-05CAC9599C02

Figures 30 , 59

##### Type material.

***Holotype*:** ♀ (IZCAS-Ar41977), Nepal, Baghmati District, Mude, Rhododendron, along the way F032, 27.69°N, 85.94°E, ca. 2653 m, 23.III.2019, C. Shrestha leg. ***Paratype***: 1♀ (IZCAS-Ar41978), same data as holotype.

##### Etymology.

The specific name is a noun in apposition taken from the type locality.

##### Diagnosis.

**Figure 30.**
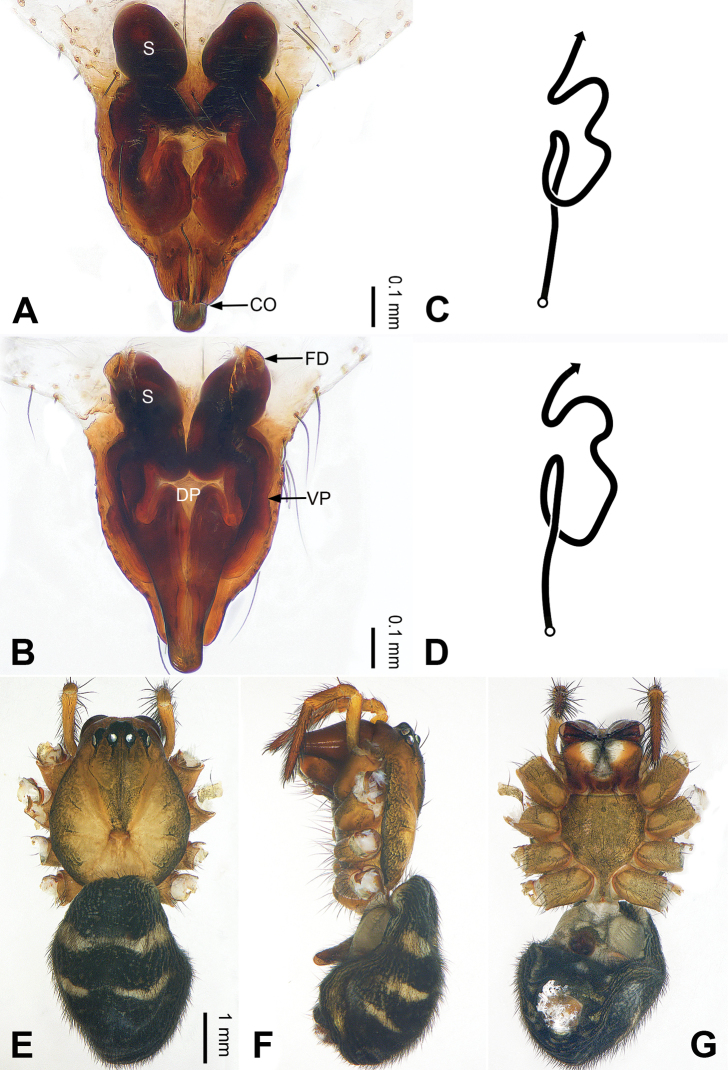
Epigyne and habitus of *Pimoa mude* sp. nov., female holotype **A** epigyne, ventral view **B** schematic course of internal duct system, ventral view **C** vulva, dorsal view **D** schematic course of internal duct system, dorsal view **E** female habitus, dorsal view **F** female habitus, lateral view **G** female habitus, ventral view. Abbreviations: CO = copulatory opening; DP = dorsal plate of the epigyne; FD = fertilization duct; S = spermatheca; VP = ventral plate of epigyne. Scale bars: equal for **E–G**.

*Pimoa mude* sp. nov. resembles those of *P. daman* sp. nov. (Fig. 6A–D) and *P. zhigangi* sp. nov. (Fig. 53A–D) but can be distinguished from the nearly oval spermathecae separated by a short distance (Fig. 30A) (vs. triangular, unseparated in *P. daman* sp. nov. and nearly round, separated by ca. 1/2 width of a spermatheca in *P. zhigangi* sp. nov.) and also distinguished from *P. daman* sp. nov. by the narrow ventral plate (Fig. 30A) (vs. wider), from *P. zhigangi* sp. nov. by the distally narrow ventral plate (Fig. 30A) (vs. broad), and by the distally blunt dorsal plate (Fig. 30B) (vs. pointed).

##### Description.

**Female (*holotype*)**: Total length 6.25. Carapace 3.06 long, 2.66 wide. Abdomen 3.19 long, 2.63 wide. Eye sizes and interdistances: AME 0.18, ALE 0.21, PME 0.17, PLE 0.22; AME-AME 0.10, AME-ALE 0.15, PME-PME 0.17, PME-PLE 0.22. Leg measurements: I: 22.18 (6.31, 7.28, 6.00, 2.59); II: 18.63 (5.38, 6.25, 4.97, 2.03); III: 13.75 (4.37, 4.13, 3.69, 1.56); IV: 17.70 (5.39, 5.84, 4.69, 1.78). Habitus as in Fig. 30E–G. Carapace yellow with black lateral margins; thoracic fovea and radial grooves distinct; sternum brownish. Abdomen black with yellowish transverse chevrons. Legs brownish with black annulations. Epigyne (Fig. 30A–D): tongue-shaped; ventral plate broad, distally narrow, width ca. 1/2 of length; dorsal plates narrow, with a tip distally, width ca. 1/2 of length; copulatory openings distinct; spermathecae nearly oval, separated by short distance; fertilization ducts yellow, medially oriented.

**Male**: Unknown.

##### Distribution.

Known only from the type locality, Baghmati District, Nepal (Fig. 59).

#### Pimoa muli

Taxon classificationAnimaliaAraneaePimoidae

Zhang & Li sp. nov.

DB830ABA-5607-563F-B48D-5109F884D0B2

http://zoobank.org/634382FA-7F33-464C-9CE3-A3207CE228A7

**Figure 31.**
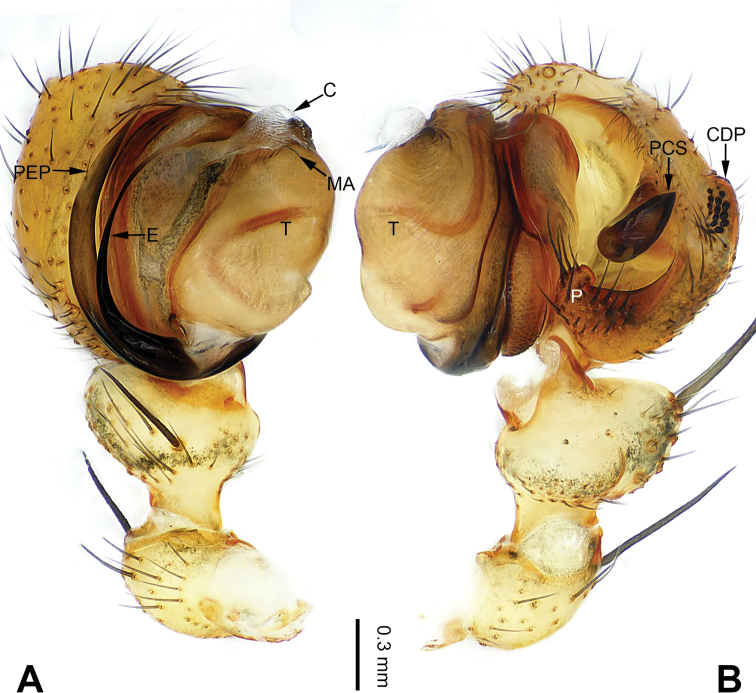
Left palp of *Pimoa muli* sp. nov., holotype **A** prolateral view **B** retrolateral view. Abbreviations: C = conductor; CDP = cymbial denticulate process; E = embolus; MA = median apophysis; P = paracymbium; PCS = pimoid cymbial sclerite; PEP = pimoid embolic process; T = tegulum. Scale bar: equal for **A, B**.

**Figure 32.**
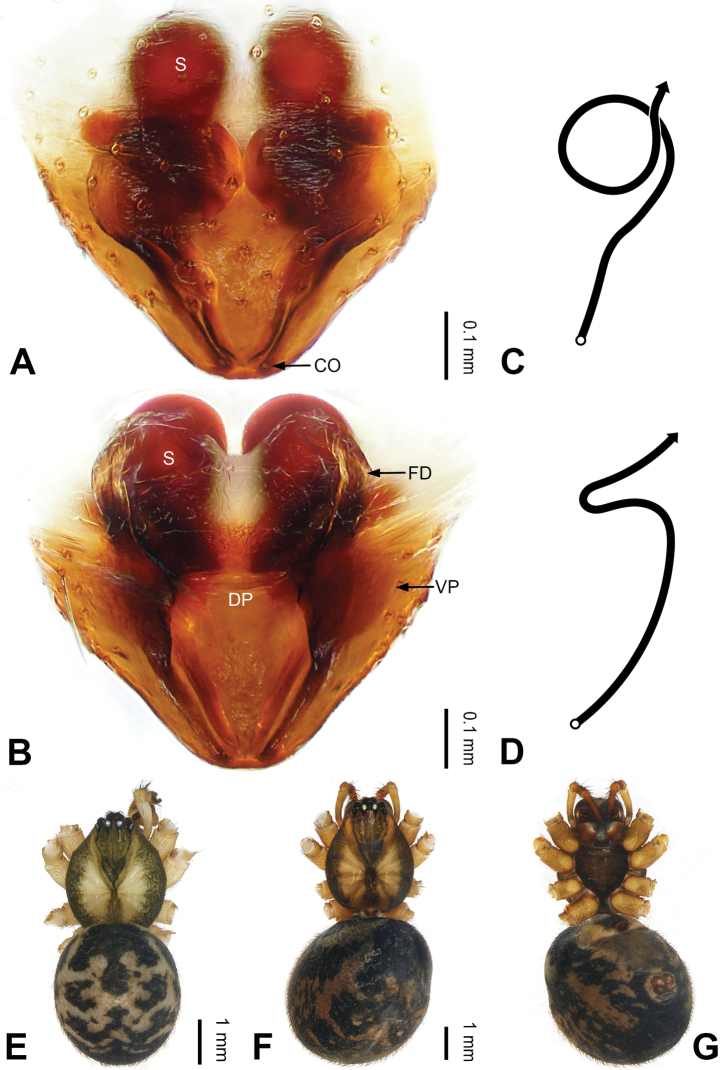
Epigyne and habitus of *Pimoa muli* sp. nov., female paratype and male holotype **A** epigyne, ventral view **B** vulva, dorsal view **C** schematic course of internal duct system, ventral view **D** schematic course of internal duct system, dorsal view **E** male habitus, dorsal view **F** female habitus, dorsal view **G** female habitus, ventral view. Abbreviations: CO = copulatory opening; DP = dorsal plate of the epigyne; FD = fertilization duct; S = spermatheca; VP = ventral plate of epigyne. Scale bars: equal for **F, G**.

Figures 31 , 32 , 56 , 59

##### Type material.

***Holotype*:** ♂ (IZCAS-Ar41979), China, Sichuan, Muli County, Chutouwan Village, 28.02°N, 101.30°E, ca. 3674 m, 11.IX.2020, Z. Chen leg. ***Paratypes***: 1♂2♀ (IZCAS-Ar41980-Ar41982), same data as holotype.

##### Etymology.

The specific name is a noun in apposition taken from the type locality.

##### Diagnosis.

The male of *Pimoa muli* sp. nov. resembles those of *P. clavata* Xu & Li, 2007 (see Xu and Li 2007: 487, figs 21–29) and *P. sinuosa* Hormiga, 1994 (see Hormiga 1994a: 67, figs 256–265) but can be distinguished from *P. clavata* by the embolus subequal to the pimoid embolic process, beginning at the 8:00 o’clock position (Fig. 56C) (vs. longer than pimoid embolic process, the posterior part of embolus beginning at the 4:30 o’clock position), and from *P. sinuosa* by the narrow pimoid cymbial sclerite, the short cymbial denticulate process (Fig. 56C) (vs. large and wide pimoid cymbial sclerite, distally narrow and curved cymbial denticulate process). The female of *P. muli* sp. nov. resembles those of *P. clavata* (see Xu and Li 2007: 487, figs 30–34) and *P. gandhii* Hormiga, 1994 (see Hormiga 1994a: 60, figs 224–231) but can be distinguished from *P. clavata* by the subtriangular epigynum (Fig. 32A) (vs. trapezoidal) and from *P. gandhii* by the dorsal plate shorter than the ventral plate (Fig. 32B) (vs. dorsal plate extending beyond the ventral plate).

##### Description.

**Male (*holotype*)**: Total length 5.58. Carapace 2.49 long, 2.22 wide. Abdomen 3.09 long, 2.81 wide. Eye sizes and interdistances: AME 0.16, ALE 0.17, PME 0.15, PLE 0.16; AME-AME 0.14, AME-ALE 0.11, PME-PME 0.12, PME-PLE 0.14. Leg measurements: I: 18.22 (5.53, 5.87, 4.88, 1.94); II: 18.84 (5.34, 6.31, 5.47, 1.72); III: 11.29 (3.44, 3.57, 3.09, 1.19); IV: 13.09 (3.94, 4.29, 3.66, 1.20). Habitus as in Fig. 32E. Carapace yellowish with black lateral margins; thoracic fovea and radial grooves distinct; sternum brownish. Abdomen black with yellow transverse chevrons and cross band extending to the medial part, nearly oval. Legs yellowish with black annulations, especially distinct on legs III and IV. Palp (Figs 31A, B, 56C): short, ca. 1/2 of cymbial length, with several macrosetae and a dorsal process; paracymbium short, ca. 1/3 of cymbial length, hook-shaped; pimoid cymbial sclerite U-shaped, ca. 1/3 of cymbial length; cymbial denticulate process short, with more than 13 cuspules; median apophysis slender; conductor distinct, membranous with scales; pimoid embolic process broad, distally pointed, almost as long as embolus; embolus beginning at the 8:00 o’clock position; embolic tooth absent.

**Female (*paratype*)**: Total length 8.77. Carapace 3.06 long, 2.47 wide. Abdomen 5.71 long, 3.78 wide. Eye sizes and interdistances: AME 0.17, ALE 0.16, PME 0.15, PLE 0.17; AME-AME 0.09, AME-ALE 0.10, PME-PME 0.13, PME-PLE 0.17. Leg measurements: I: 15.53 (4.56, 5.44, 3.69, 1.84); II: 13.40 (4.06, 4.47, 3.34, 1.53); III: 9.73 (2.94, 3.16, 2.47, 1.16); IV: 12.22 (3.56, 4.16, 3.19, 1.31). Habitus as in Fig. 32F, G. Carapace yellowish with black lateral margins; thoracic fovea and radial grooves distinct; sternum brownish. Abdomen black with yellowish transverse bands. Legs yellowish with black annulations, especially distinct on legs III and IV. Epigyne (Fig. 32A–D): subtriangular; ventral plate broad, width subequal to length; dorsal plate narrow, shorter than ventral plate; copulatory openings distinct; spermathecae round, close to each other; fertilization ducts yellowish, anteriorly oriented.

##### Distribution.

Known only from the type locality, Sichuan, China (Fig. 59).

#### Pimoa naran

Taxon classificationAnimaliaAraneaePimoidae

Zhang & Li sp. nov.

88CDBA28-2A5B-5A5F-83B6-26640EE47786

http://zoobank.org/3A1F49BD-79F0-4E24-93C6-70324A1C71CD

Figures 33 , 59

##### Type material.

***Holotype*:** ♀ (IZCAS-Ar41983), Pakistan, Khyber Pakhtunkhwa, Naran, along Saiful Muluk Road, 34.91°N, 73.66°E, ca. 2553 m, 10.VI.2019, A. Ali leg. ***Paratype***: 1♀ (IZCAS-Ar41984), same data as holotype.

##### Etymology.

The specific name is a noun in apposition taken from the type locality.

##### Diagnosis.

**Figure 33.**
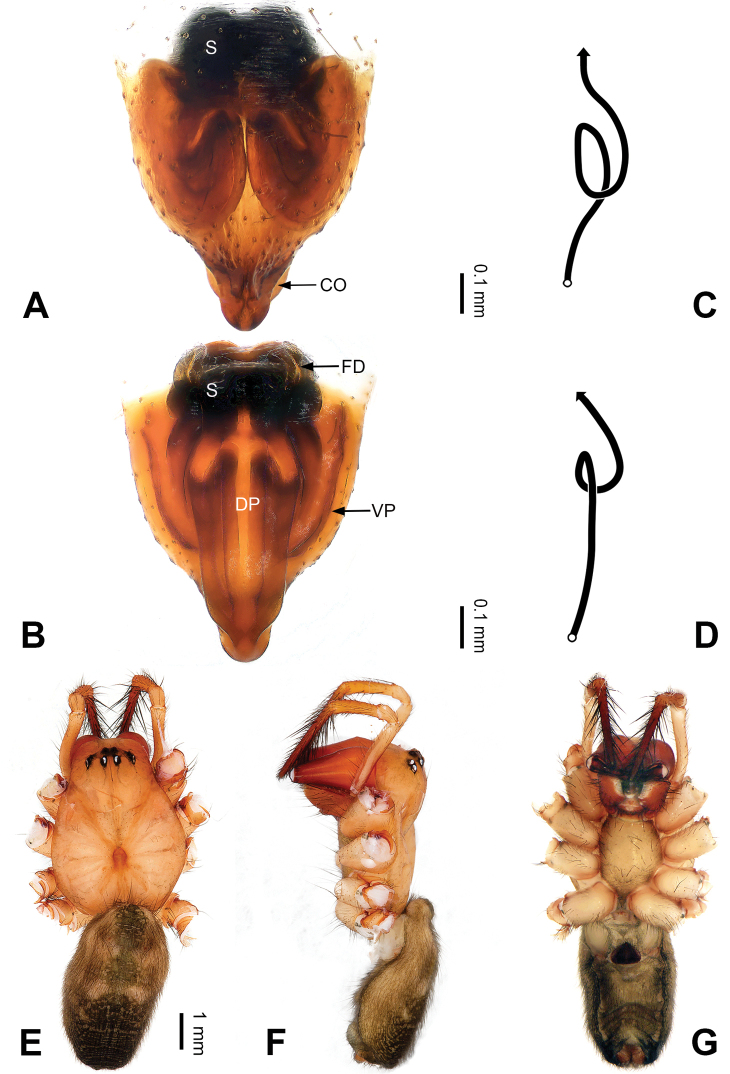
Epigyne and habitus of *Pimoa naran* sp. nov., female holotype **A** epigyne, ventral view **B** schematic course of internal duct system, ventral view **C** vulva, dorsal view **D** schematic course of internal duct system, dorsal view **E** female habitus, dorsal view **F** female habitus, lateral view **G** female habitus, ventral view. Abbreviations: CO = copulatory opening; DP = dorsal plate of the epigyne; FD = fertilization duct; S = spermatheca; VP = ventral plate of epigyne. Scale bars: equal for **E–G**.

*Pimoa naran* sp. nov. resembles those of *P. lata* (see Xu and Li 2009: 57, figs 1–8; Zhang and Li 2019: 6, fig. 4A, B) and *P. samyai* (see Zhang et al. 2020: 97, fig. 13A–D) but can be distinguished by the distally narrow ventral plate (Fig. 33A) (vs. broad) and by the round, unseparated spermatheca (Fig. 33A) (vs. pear-shaped spermatheca separated by a short distance in *P. lata*, and the spermathecae are nearly oval, separated by ca. 1/2 width of a spermatheca in *P. samyai*).

##### Description.

**Female (*holotype*)**: Total length 9.16. Carapace 4.36 long, 3.56 wide. Abdomen 4.80 long, 2.76 wide. Eye sizes and interdistances: AME 0.12, ALE 0.15, PME 0.13, PLE 0.19; AME-AME 0.16, AME-ALE 0.28, PME-PME 0.23, PME-PLE 0.37. Leg measurements: I: 40.75 (11.16, 13.60, 12.36, 3.63); II: 36.44 (10.16, 12.12, 10.92, 3.24); III: 25.74 (7.77, 8.25, 7.56, 2.16); IV: 29.88 (8.63, 10.81, 7.88, 2.56). Habitus as in Fig. 33E–G. Carapace yellowish; thoracic fovea and radial grooves distinct; sternum brownish. Abdomen black with yellowish transverse bands and vertical bands fused. Legs yellowish without annulations. Epigyne (Fig. 33A–D): subtriangular; ventral plates broad, distally narrow with a tip, length subequal to width; dorsal plate longer than wide, nearly tongue-shaped; copulatory openings distinct; spermathecae round, unseparated; fertilization ducts yellowish, medially oriented.

**Male**: Unknown.

##### Distribution.

Known only from the type locality, Khyber Pakhtunkhwa, Pakistan (Fig. 59).

#### Pimoa ninglang

Taxon classificationAnimaliaAraneaePimoidae

Zhang & Li sp. nov.

B5A62722-53D7-551E-B13E-9098A6A583D0

http://zoobank.org/6A56E9BC-67A7-440D-9352-101E53A5C73D

Figures 34 , 59

##### Type material.

***Holotype*:** ♀ (IZCAS-Ar41985), China, Yunnan, Lijiang, Ninglang Yi Autonomous County, Lugu Lake, Nvshen Cave, 27.62°N, 100.81°E, ca. 3540 m, 5.VII.2010, Q. Zhao leg.

##### Etymology.

The specific name is a noun in apposition taken from the type locality.

##### Diagnosis.

**Figure 34.**
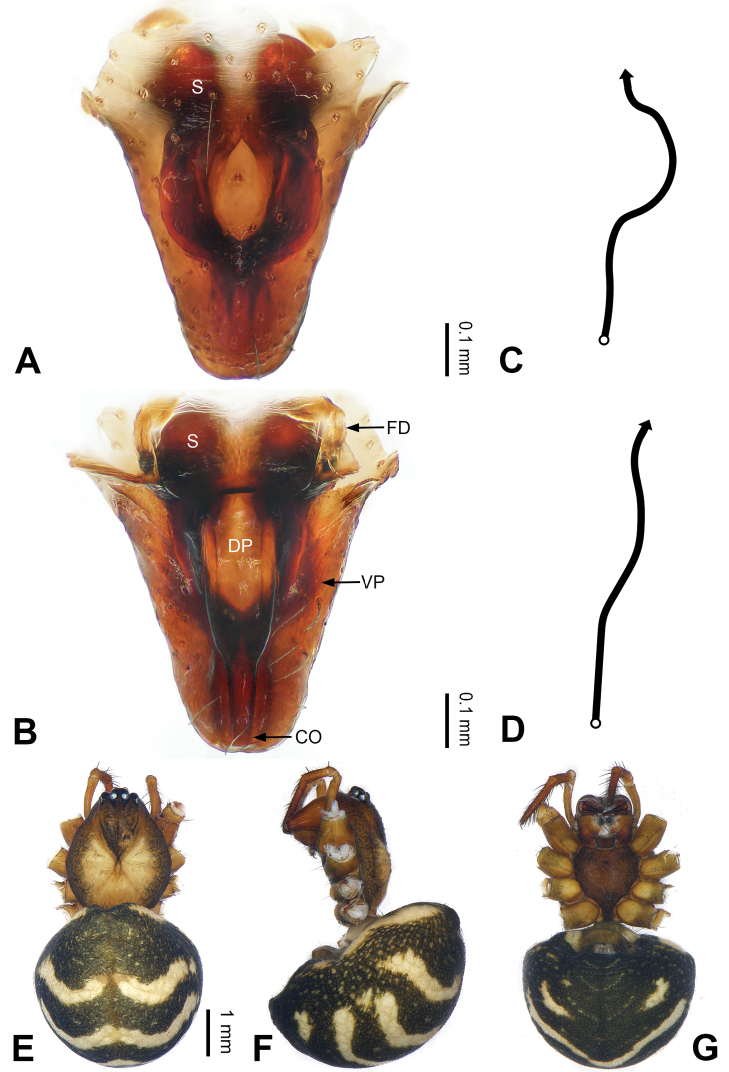
Epigyne and habitus of *Pimoa ninglang* sp. nov., female holotype **A** epigyne, ventral view **B** schematic course of internal duct system, ventral view **C** vulva, dorsal view **D** schematic course of internal duct system, dorsal view **E** female habitus, dorsal view **F** female habitus, lateral view **G** female habitus, ventral view. Abbreviations: CO = copulatory opening; DP = dorsal plate of the epigyne; FD = fertilization duct; S = spermatheca; VP = ventral plate of epigyne. Scale bars: equal for **E–G**.

*Pimoa ninglang* sp. nov. resembles those of *P. clavata* (see Xu and Li 2007: 487, figs 30–34) and *P. gandhii* (see Hormiga 1994a: 60, figs 224–231) but can be distinguished from *P. clavata* by the narrow dorsal plate (Fig. 34B) (vs. wider) and from *P. gandhii* by the trapezoidal ventral plate (Fig. 34A) (vs. triangular) and the fusiform dorsal plate (Fig. 34B) (vs. tongue-shaped).

##### Description.

**Female (*holotype*)**: Total length 5.31. Carapace 2.43 long, 2.28 wide. Abdomen 2.88 long, 3.03 wide. Eye sizes and interdistances: AME 0.14, ALE 0.13, PME 0.17, PLE 0.15; AME-AME 0.09, AME-ALE 0.07, PME-PME 0.10, PME-PLE 0.15. Leg measurements: I: – (5.06, –, –, –); II: 14.47 (4.31, 4.91, 3.53, 1.72); III: 10.71 (3.31, 3.40, 2.66, 1.34); IV: 14.25 (4.34, 4.78, 3.66, 1.47). Habitus as in Fig. 34E–G. Carapace yellowish with black lateral margins; thoracic fovea and radial grooves distinct; sternum brownish. Abdomen grayish with yellowish transverse chevrons. Legs brownish with black annulations. Epigyne (Fig. 34A–D): trapezoidal; ventral plate broad, length subequal to width; dorsal plates narrow; copulatory openings distinct; spermathecae round, separated by ca. 1/3 width of spermatheca; fertilization ducts yellow, laterally oriented.

**Male**: Unknown.

##### Distribution.

Known only from the type locality, Yunnan, China (Fig. 59).

#### Pimoa nyalam

Taxon classificationAnimaliaAraneaePimoidae

Zhang & Li sp. nov.

0294AC86-D114-54C7-95DE-9D1A599A6B75

http://zoobank.org/590E23F7-C150-4CE4-A2A2-6B86B60B4992

**Figure 35.**
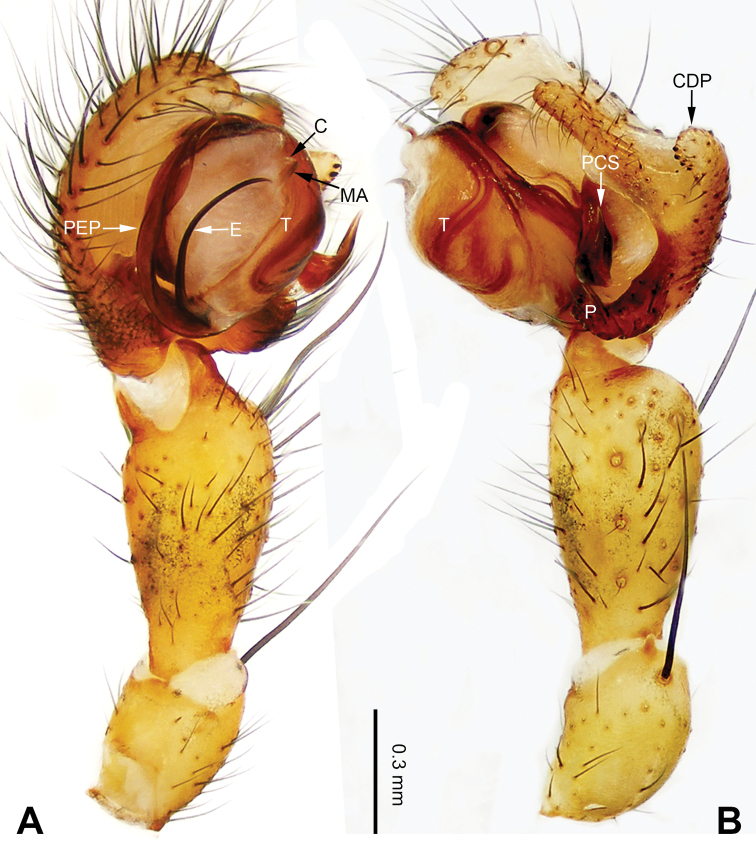
Left palp of *Pimoa nyalam* sp. nov., holotype **A** prolateral view **B** retrolateral view. Abbreviations: C = conductor; CDP = cymbial denticulate process; E = embolus; MA = median apophysis; P = paracymbium; PCS = pimoid cymbial sclerite; PEP = pimoid embolic process; T = tegulum. Scale bar: equal for **A, B**.

**Figure 36.**
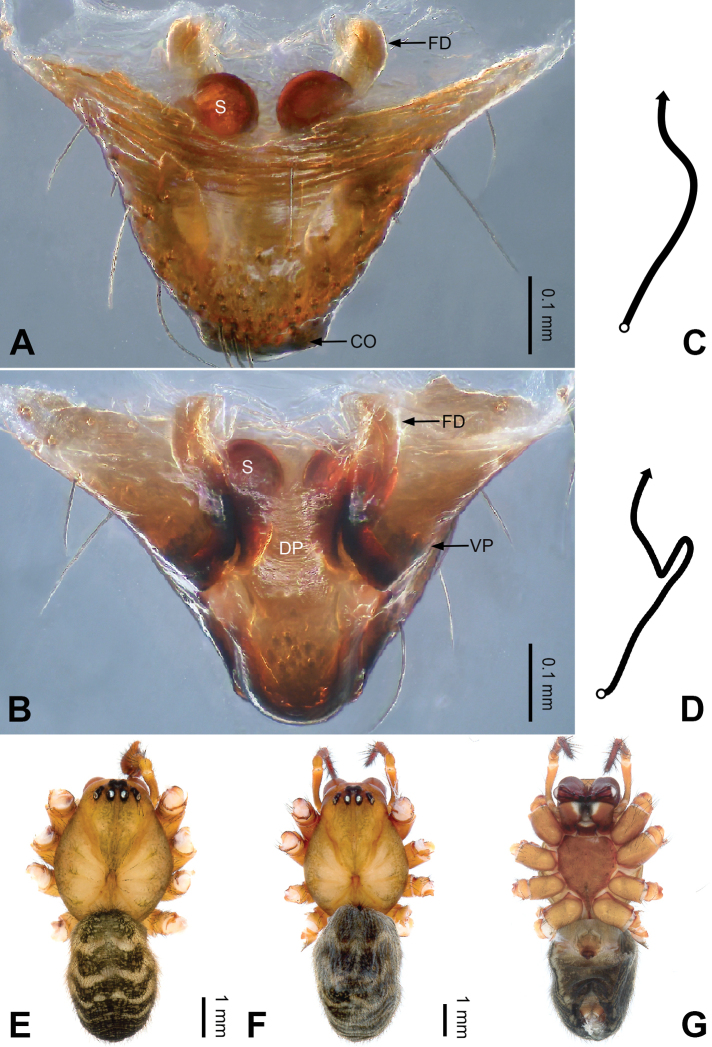
Epigyne and habitus of *Pimoa nyalam* sp. nov., female paratype and male holotype **A** epigyne, ventral view **B** vulva, dorsal view **C** schematic course of internal duct system, ventral view **D** schematic course of internal duct system, dorsal view **E** male habitus, dorsal view **F** female habitus, dorsal view **G** female habitus, ventral view. Abbreviations: CO = copulatory opening; DP = dorsal plate of the epigyne; FD = fertilization duct; S = spermatheca; VP = ventral plate of epigyne. Scale bars: equal for **F, G**.

Figures 35 , 36 , 56 , 59

##### Type material.

***Holotype*:** ♂ (IZCAS-Ar41986), China, Tibet, Shigatse, Nyalam County, Zham Town, near the dam, 28.09°N, 86.00°E, ca. 3326 m, 9.VII.2019, X. Zhang, Z. Bai and J. Liu leg. ***Paratypes***: 1♂2♀ (IZCAS-Ar41987-Ar41989), same data as holotype.

##### Etymology.

The specific name is a noun in apposition taken from the type locality.

##### Diagnosis.

The male of *Pimoa nyalam* sp. nov. resembles those of *P. crispa* (see Hormiga 1994a: 63, figs 233–238; Hormiga 1994b: fig. 1A, B) and *P. gyirong* sp. nov. (see Figs 15A, B, 55A) but can be distinguished by the short and distally narrowed cymbial denticulate process (Fig. 56D) (vs. large and laterally broad in *P. crispa* and wide in *P. gyirong* sp. nov.) and also from *P. gyirong* sp. nov. by the longer palpal tibia (Fig. 56D) (vs. shorter) and the smaller pimoid cymbial sclerite (Fig. 56D) (vs. large). The female of *P. nyalam* sp. nov. also resembles those of *P. crispa* (see Hormiga 1994a: 63, figs 239–247) and *P. samyai* (see Zhang et al. 2020: 97, fig. 13A–D) but can be distinguished from *P. crispa* by the distally blunt dorsal plate (Fig. 36B) (vs. distally narrow, with a tip) and from *P. samyai* by the trapezoidal dorsal plate (Fig. 36A) (vs. tongue shaped).

##### Description.

**Male (*holotype*)**: Total length 6.53. Carapace 3.28 long, 2.92 wide. Abdomen 3.25 long, 2.19 wide. Eye sizes and interdistances: AME 0.09, ALE 0.11, PME 0.10, PLE 0.09; AME-AME 0.08, AME-ALE 0.17, PME-PME 0.16, PME-PLE 0.22. Leg measurements: I: 39.79 (10.66, 12.28, 12.67, 4.18); II: 34.58 (9.51, 10.68, 10.84, 3.55); III: 22.94 (6.59, 7.13, 7.16, 2.06); IV: 28.70 (7.97, 9.13, 8.88, 2.72). Habitus as in Fig. 36E. Carapace yellowish, with black lateral margins; thoracic fovea and radial grooves distinct; sternum brownish. Abdomen grayish with yellow transverse chevrons, nearly oval. Legs brownish without annulations. Palp (Figs 35A, B, 56D): patella short, ca. 1/2 of tibial length, with one retrolateral macroseta; tibia long, almost as long as cymbial length, with several macrosetae and a dorsal process; paracymbium short, ca. 1/3 of cymbial length, finger-shaped; pimoid cymbial sclerite V-shaped, ca. 1/3 of cymbial length; cymbial denticulate process short and distally narrow, with more than 7 cuspules; median apophysis curved; conductor indistinct; pimoid embolic process distally pointed, longer than embolus; embolus beginning at the 7:00 o’clock position; embolic tooth absent.

**Female (*paratype*)**: Total length 8.20. Carapace 3.92 long, 3.38 wide. Abdomen 4.28 long, 2.36 wide. Eye sizes and interdistances: AME 0.19, ALE 0.23, PME 0.18, PLE 0.19; AME-AME 0.13, AME-ALE 0.26, PME-PME 0.22, PME-PLE 0.29. Leg measurements: I: 32.30 (8.75, 10.91, 9.23, 3.41); II: 29.18 (8.34, 9.60, 8.27, 2.97); III: 20.85 (6.56, 6.53, 5.77, 1.99); IV: 25.77 (7.09, 9.02, 7.38, 2.28). Habitus as in Fig. 36F, G. Carapace yellowish; sternum brownish. Abdomen grayish with yellowish transverse chevrons. Legs brownish with black annulations. Epigyne (Fig. 36A–D): trapezoidal; ventral plate broad, length subequal to width; dorsal plate broad, distally blunt; copulatory openings distinct; spermathecae oval, separated by ca. 1/3 width of a spermatheca; fertilization ducts yellowish, laterally oriented.

##### Distribution.

Known only from the type locality, Tibet, China (Fig. 59).

#### Pimoa phaplu

Taxon classificationAnimaliaAraneaePimoidae

Zhang & Li sp. nov.

F7B5E07C-AE47-52F8-8D50-D0D0EDD0E73B

http://zoobank.org/C4C02082-8D11-421C-9865-9F3EBD121173

**Figure 37.**
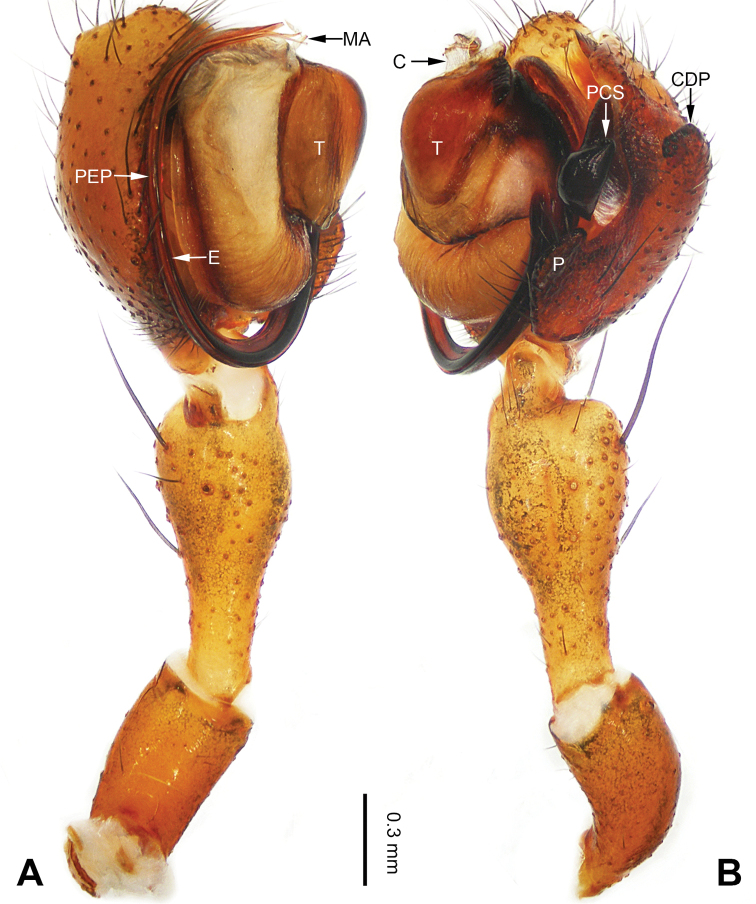
Left palp of *Pimoa phaplu* sp. nov., holotype **A** prolateral view **B** retrolateral view. Abbreviations: C = conductor; CDP = cymbial denticulate process; E = embolus; MA = median apophysis; P = paracymbium; PCS = pimoid cymbial sclerite; PEP = pimoid embolic process; T = tegulum. Scale bar: equal for **A, B**.

**Figure 38.**
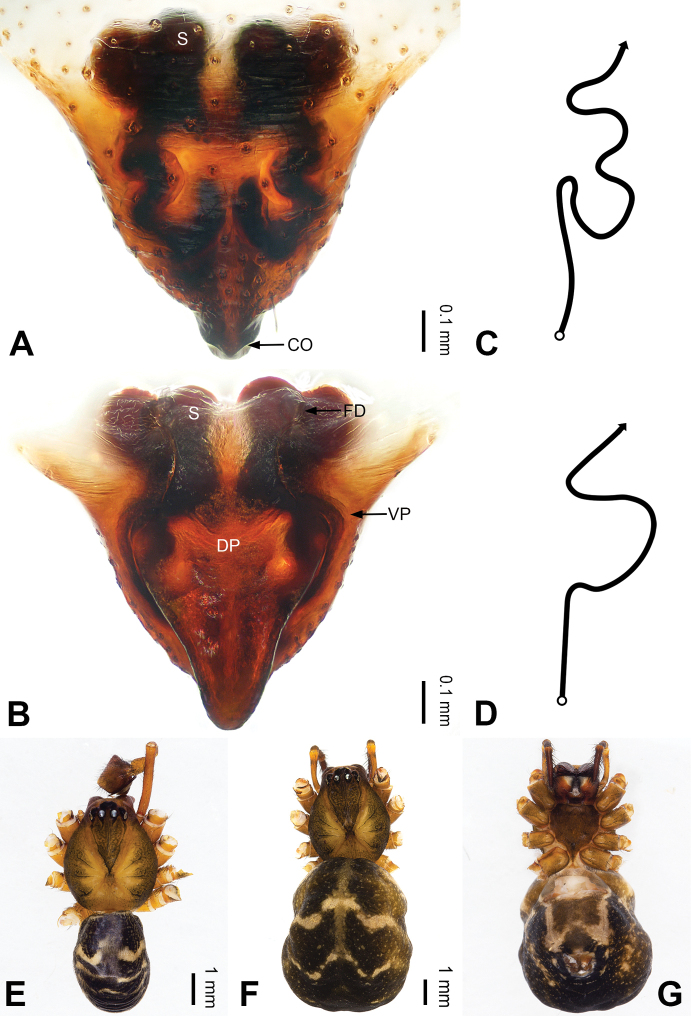
Epigyne and habitus of *Pimoa phaplu* sp. nov., female paratype and male holotype **A** epigyne, ventral view **B** vulva, dorsal view **C** schematic course of internal duct system, ventral view **D** schematic course of internal duct system, dorsal view **E** male habitus, dorsal view **F** female habitus, dorsal view **G** female habitus, ventral view. Abbreviations: CO = copulatory opening; DP = dorsal plate of the epigyne; FD = fertilization duct; S = spermatheca; VP = ventral plate of epigyne. Scale bars: equal for **F, G**.

Figures 37 , 38 , 57 , 59

##### Type material.

***Holotype*:** ♂ (IZCAS-Ar41990), Nepal, Sagarmatha District, Phaplu Airport, 27.53°N, 86.60°E, ca. 2530 m, 26.III.2019, C. Shrestha leg. ***Paratypes***: 2♀ (IZCAS-Ar41991-Ar41992), same data as holotype.

##### Etymology.

The specific name is a noun in apposition taken from the type locality.

##### Diagnosis.

The male of *Pimoa phaplu* sp. nov. resembles those of *P. nematoides* Hormiga, 1994 (see Hormiga 1994a: 71, figs 285–289) and *P. yadong* (see Zhang et al. 2020: 99, fig. 14A–C) but can be distinguished from *P. nematoides* by the longer palpal patella and tibia (Figs 37A, B, 57A) (vs. short) and from *P. yadong* by the unsclerotized median apophysis (Fig. 57A) (vs. sclerotized) and also by the membranous, distinct conductor (Fig. 57A) (vs. indistinct). The female of *P. phaplu* sp. nov. resembles those of *P. sinuosa* (see Hormiga 1994a: 67, figs 266–274) and *P. yadong* (see Zhang et al. 2020: 99, fig. 15A–D) but can be distinguished by the bean-shaped spermathecae, separated by a short distance (Fig. 38A) (vs. unseparated in *P. sinuosa* and round in *P. yadong*) and also from *P. yadong* by the distally narrow dorsal plate (Fig. 38B) (vs. distally blunt).

##### Description.

**Male (*holotype*)**: Total length 7.05. Carapace 3.53 long, 3.09 wide. Abdomen 3.52 long, 2.47 wide. Eye sizes and interdistances: AME 0.21, ALE 0.20, PME 0.21, PLE 0.21; AME-AME 0.15, AME-ALE 0.14, PME-PME 0.17, PME-PLE 0.19. Leg measurements: I: 25.35 (7.63, 7.96, 6.92, 2.84); II: 20.32 (6.16, 6.24, 5.52, 2.40); III: 14.64 (4.60, 4.56, 3.96, 1.52); IV: 20.68 (6.06, 6.81, 5.78, 2.03). Habitus as in Fig. 38E. Carapace yellowish with black lateral margins; thoracic fovea and radial grooves distinct; sternum brownish. Abdomen black with slightly yellowish transverse bands. Legs brownish with black annulations. Palp (Figs 37A, B, 57A): patella long, ca. 1/2 of tibial length; tibia long, subequal to cymbial length, with a dorsal process; paracymbium large, ca. 1/2 of cymbial length; pimoid cymbial sclerite L-shaped, ca. 1/2 of cymbial length; cymbial denticulate process short and distally pointed, with more than 8 cuspules; median apophysis slender; conductor membranous and distinct; pimoid embolic process almost as long as embolus; embolus beginning at the 3:00 o’clock position; embolic tooth absent.

**Female (*paratype*)**: Total length 9.40. Carapace 3.64 long, 3.24 wide. Abdomen 5.76 long, 4.60 wide. Eye sizes and interdistances: AME 0.24, ALE 0.26, PME 0.22, PLE 0.25; AME-AME 0.13, AME-ALE 0.18, PME-PME 0.23, PME-PLE 0.26. Leg measurements: I: 23.00 (6.68, 7.68, 6.04, 2.60); II: 21.20 (6.28, 6.96, 5.60, 2.36); III: 14.16 (4.32, 4.40, 3.76, 1.68); IV: 20.13 (5.96, 6.44, 5.92, 1.81). Habitus as in Fig. 38F, G. Carapace yellowish with black lateral margins; thoracic fovea and radial grooves distinct; sternum brownish. Abdomen black with yellowish transverse chevrons and vertical band extending to the medial part. Legs brownish with black annulations. Epigyne (Fig. 38A–D): subtriangular; ventral plate broad, length subequal to width; dorsal plate tongue shaped, distally extending beyond the ventral plate; copulatory openings distinct; spermathecae bean shaped, separated by short distance; fertilization ducts hyaline, medially oriented.

##### Distribution.

Known only from the type locality, Sagarmatha District, Nepal (Fig. 59).

#### Pimoa putou

Taxon classificationAnimaliaAraneaePimoidae

Zhang & Li sp. nov.

4ED3E4FD-104B-5E1E-9E7B-D85109A213DD

http://zoobank.org/839F876D-C10B-44B6-898C-685B7F4E07E9

Figures 39 , 59

##### Type material.

***Holotype*:** ♀ (IZCAS-Ar41993), China, Sichuan, Li County, Putou Village, Bipeng Valley to Macao Valley, 31.36°N, 102.85°E, ca. 2805 m, 21.XI.2019, Z. Chen leg. ***Paratype***: 1♀ (IZCAS-Ar41994), same data as holotype.

##### Etymology.

The specific name is a noun in apposition taken from the type locality.

##### Diagnosis.

**Figure 39.**
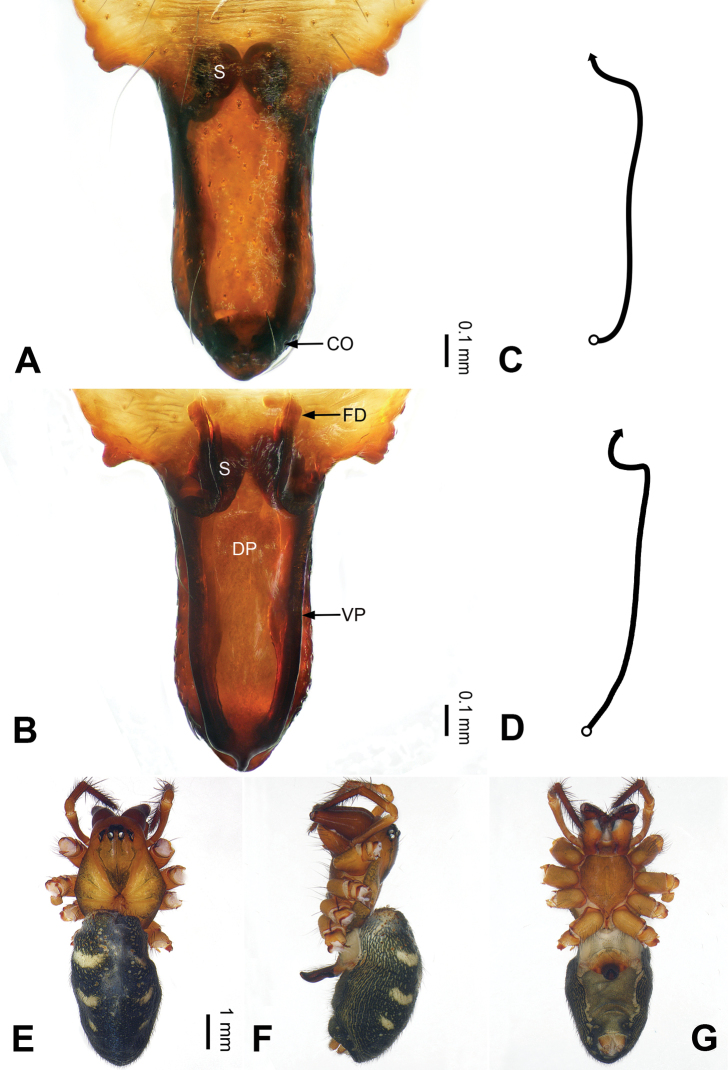
Epigyne and habitus of *Pimoa putou* sp. nov., female holotype **A** epigyne, ventral view **B** schematic course of internal duct system, ventral view **C** vulva, dorsal view **D** schematic course of internal duct system, dorsal view **E** female habitus, dorsal view **F** female habitus, lateral view **G** female habitus, ventral view. Abbreviations: CO = copulatory opening; DP = dorsal plate of the epigyne; FD = fertilization duct; S = spermatheca; VP = ventral plate of epigyne. Scale bars: equal for **E–G**.

*Pimoa putou* sp. nov. resembles those of *P. jinchuan* sp. nov. (Fig. 20A–D) and *P. yele* sp. nov. (Fig. 50A–D) but can be distinguished by the tongue-shaped ventral plate with two processes proximally (Fig. 39A) (vs. distally curved in *P. jinchuan* sp. nov. and broad in *P. yele* sp. nov.) and also distinguished from *P. yele* sp. nov. by the ventral plate slightly longer than the dorsal plate (Fig. 39B) (vs. shorter).

##### Description.

**Female (*holotype*)**: Total length 7.26. Carapace 2.63 long, 2.60 wide. Abdomen 4.63 long, 2.56 wide. Eye sizes and interdistances: AME 0.16, ALE 0.18, PME 0.17, PLE 0.18; AME-AME 0.13, AME-ALE 0.16, PME-PME 0.07, PME-PLE 0.22. Leg measurements: I: 28.10 (7.96, 9.50, 8.28, 2.36); II: 23.74 (6.53, 7.77, 7.16, 2.28); III: 17.06 (5.22, 5.47, 4.59, 1.78); IV: 22.13 (6.63, 7.22, 6.31, 1.97). Habitus as in Fig. 39E–G. Carapace yellow with black lateral margins; thoracic fovea and radial grooves distinct; sternum brownish. Abdomen grayish with yellowish transverse chevrons. Legs brownish without annulations. Epigyne (Fig. 39A–D): bullet-shaped; ventral plate broad, with two processes proximally, length subequal to width; dorsal plate tongue-shaped, with a tip distally, width ca. 1/2 length; copulatory openings distinct; spermathecae round, unseparated; fertilization ducts yellow, laterally oriented.

**Male**: Unknown.

##### Distribution.

Known only from the type locality, Sichuan, China (Fig. 59).

#### Pimoa rara

Taxon classificationAnimaliaAraneaePimoidae

Zhang & Li sp. nov.

BEC399E0-E8CA-54C0-8044-9CDB11B4884E

http://zoobank.org/E3DE9883-5CD7-42A5-8E1E-98962426406E

Figures 40 , 59

##### Type material.

***Holotype*:** ♀ (IZCAS-Ar41995), Nepal, Karnali District, Rara National Park, 29.52°N, 82.08°E, ca. 2974 m, 13.IV.2019, C. Shrestha leg. ***Paratype***: 1♀ (IZCAS-Ar41996), same data as holotype.

##### Etymology.

The specific name is a noun in apposition taken from the type locality.

##### Diagnosis.

**Figure 40.**
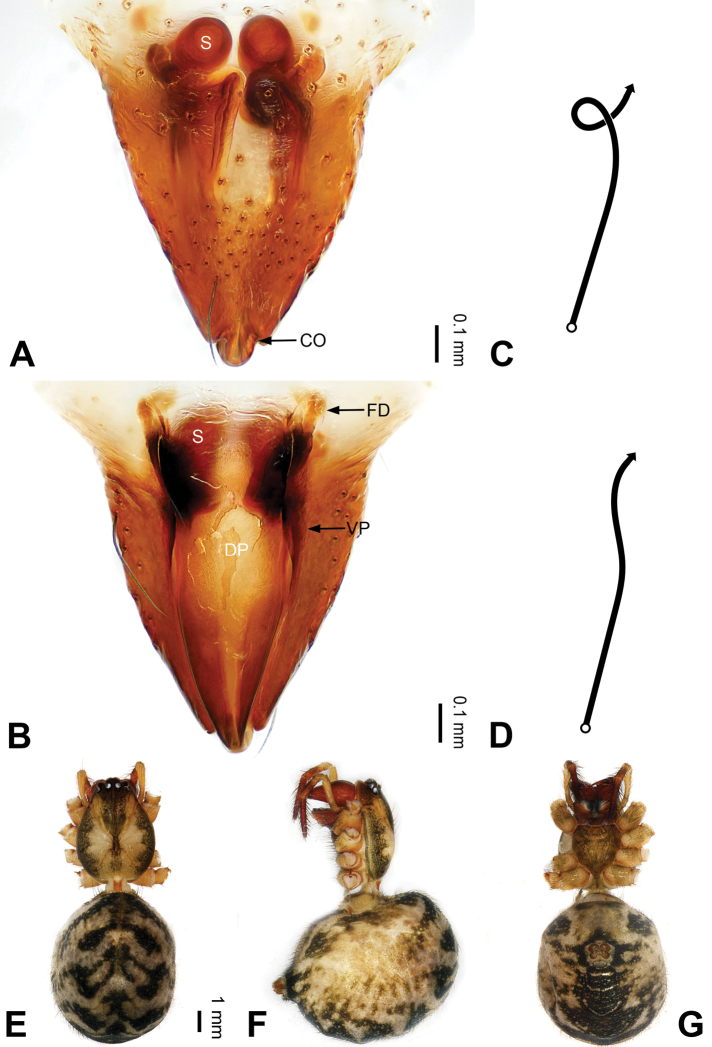
Epigyne and habitus of *Pimoa rara* sp. nov., female holotype **A** epigyne, ventral view **B** schematic course of internal duct system, ventral view **C** vulva, dorsal view **D** schematic course of internal duct system, dorsal view **E** female habitus, dorsal view **F** female habitus, lateral view **G** female habitus, ventral view. Abbreviations: CO = copulatory opening; DP = dorsal plate of the epigyne; FD = fertilization duct; S = spermatheca; VP = ventral plate of epigyne. Scale bars: equal for **E–G**.

*Pimoa rara* sp. nov. resembles those of *P. crispa* (see Hormiga 1994a: 63, figs 239–247) and *P. samyai* (see Zhang et al. 2020: 97, fig. 13A–D) but can be distinguished from *P. crispa* by the ventral plate width ca. 1/2 length (Fig. 40A) (vs. width subequal to length), from *P. samyai* by the bean-shaped spermathecae separated by a short distance (Fig. 40A) (vs. nearly oval, separated by ca. 1/2 width of a spermatheca), and by the distally pointed dorsal plate (Fig. 40B) (vs. distally blunt).

##### Description.

**Female (*holotype*)**: Total length 9.74. Carapace 4.90 long, 3.78 wide. Abdomen 4.84 long, 4.38 wide. Eye sizes and interdistances: AME 0.16, ALE 0.15, PME 0.17, PLE 0.16; AME-AME 0.14, AME-ALE 0.15, PME-PME 0.13, PME-PLE 0.18. Leg measurements: I: 16.96 (4.66, 6.08, 4.22, 2.00); II: 14.46 (4.24, 4.78, 3.63, 1.81); III: – (3.94, –, –, –); IV: – (–, –, –, –). Habitus as in Fig. 40E–G. Carapace yellowish with black lateral margins; thoracic fovea and radial grooves distinct; sternum brownish. Abdomen black with yellowish transverse bands. Legs brownish with black annulations. Epigyne (Fig. 40A–D): triangular; ventral plate broad, length subequal to width; dorsal plate narrow, width ca. 1/2 length; copulatory openings distinct; spermathecae oval, close together; fertilization ducts hyaline, laterally oriented.

**Male**: Unknown.

##### Distribution.

Known only from the type locality, Karnali District, Nepal (Fig. 59).

#### Pimoa sangri

Taxon classificationAnimaliaAraneaePimoidae

Zhang & Li sp. nov.

01CFA593-4E35-514B-904F-82FC3976BF12

http://zoobank.org/512CDF6A-E167-446F-898E-B2C0B921A032

**Figure 41.**
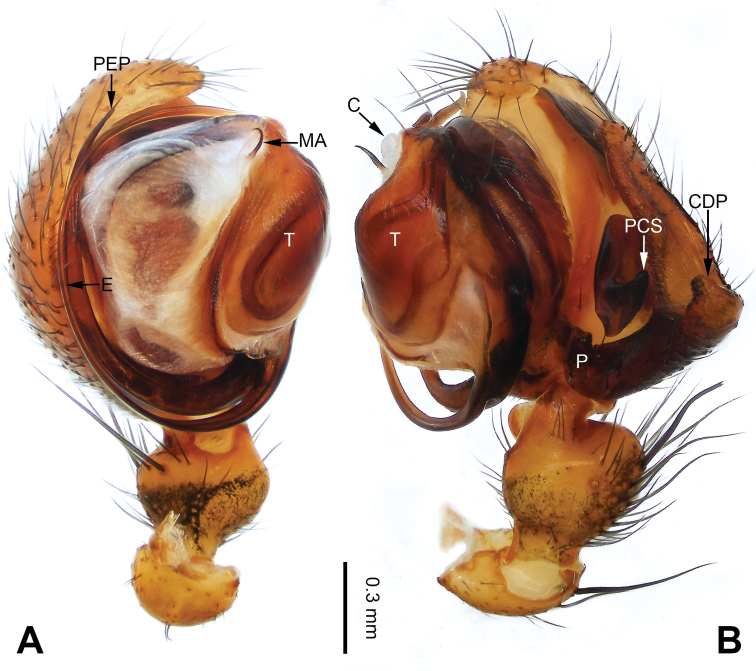
Left palp of *Pimoa sangri* sp. nov., holotype **A** prolateral view **B** retrolateral view. Abbreviations: C = conductor; CDP = cymbial denticulate process; E = embolus; MA = median apophysis; P = paracymbium; PCS = pimoid cymbial sclerite; PEP = pimoid embolic process; T = tegulum. Scale bar: equal for **A, B**.

**Figure 42.**
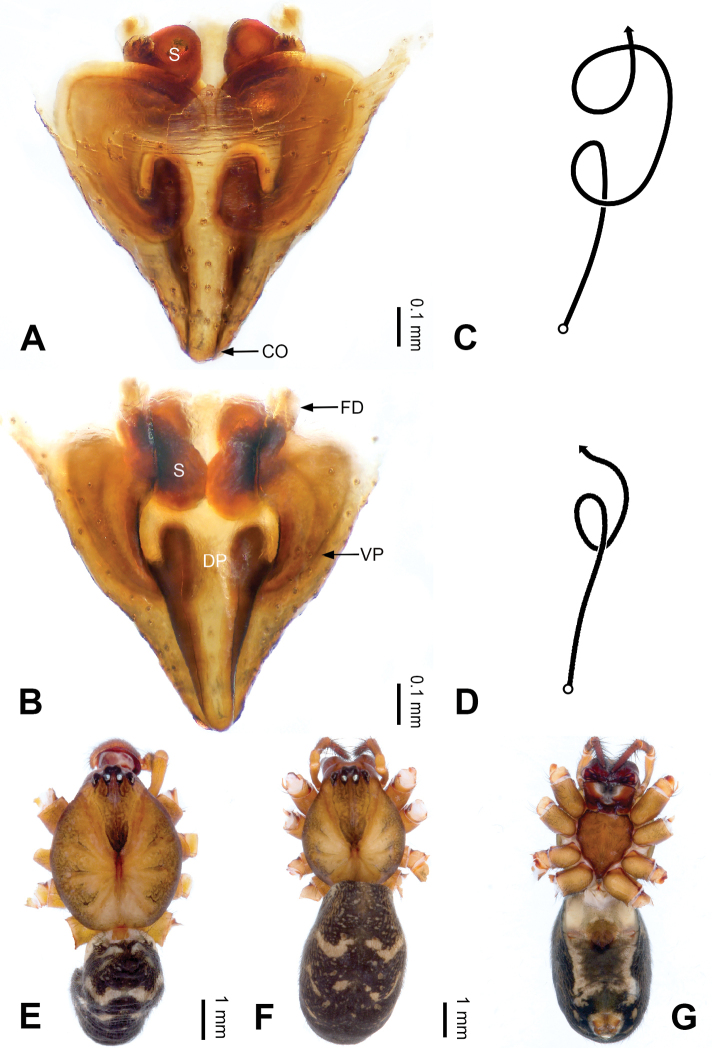
Epigyne and habitus of *Pimoa sangri* sp. nov., female paratype and male holotype **A** epigyne, ventral view **B** vulva, dorsal view **C** schematic course of internal duct system, ventral view **D** schematic course of internal duct system, dorsal view **E** male habitus, dorsal view **F** female habitus, dorsal view **G** female habitus, ventral view. Abbreviations: CO = copulatory opening; DP = dorsal plate of the epigyne; FD = fertilization duct; S = spermatheca; VP = ventral plate of epigyne. Scale bars: equal for **F, G**.

Figures 41 , 42 , 57 , 59

##### Type material.

***Holotype*:** ♂ (IZCAS-Ar41997), China, Tibet, Lhoka, along provincial highway 306 from Gyaca County to Sangri County, 29.05°N, 92.39°E, ca. 4329 m, 27.VIII.2018, X. Zhang, Z. Bai and J. Liu leg. ***Paratypes***: 1♂2♀ (IZCAS-Ar41998-Ar42000), same data as holotype.

##### Etymology.

The specific name is a noun in apposition taken from the type locality.

##### Diagnosis.

The male of *Pimoa sangri* sp. nov. resembles those of *P. gandhii* (see Hormiga 1994a: 60, figs 218–223) and *P. nyingchi* (see Zhang et al. 2020: 91, fig. 8A–C) but can be distinguished from *P. gandhii* by the V-shaped pimoid cymbial sclerite (Fig. 57B) (vs. L-shaped), by having the pimoid embolic process shorter than the embolus (Figs 41A, 57B) (vs. longer), and distinguished from *P. nyingchi* by the embolus beginning at the 4:30 o’clock position (Fig. 57B) (vs. 3:00), and the narrow cymbial denticulate process (Fig. 57B) (vs. broad). The female of *P. sangri* sp. nov. also resembles *P. gandhii* (see Hormiga 1994a: 60, figs 224–231) but can be distinguished by the wide proximal fertilization ducts (Fig. 42A) (vs. narrow) and by the rectangular spermathecae, divided into two parts (Fig. 42A) (vs. oval).

##### Description.

**Male (*holotype*)**: Total length 8.52. Carapace 4.04 long, 3.28 wide. Abdomen 4.48 long, 2.28 wide. Eye sizes and interdistances: AME 0.20, ALE 0.16, PME 0.17, PLE 0.16; AME-AME 0.13, AME-ALE 0.17, PME-PME 0.15, PME-PLE 0.25. Leg measurements: I: 28.94 (8.28, 9.41, 8.22, 3.03); II: 28.36 (7.88, 9.07, 8.75, 2.66); III: 18.87 (5.63, 5.66, 5.74, 1.84); IV: 24.38 (6.97, 7.97, 7.38, 2.06). Habitus as in Fig. 42E. Carapace yellowish with black lateral margins; thoracic fovea and radial grooves distinct; sternum brownish. Abdomen black with yellowish transverse chevrons, nearly oval. Legs brownish with black annulations. Palp (Figs 41A, B, 57B): patella short, almost as long as tibial length, with one retrolateral macroseta; tibia short, ca. 1/2 of cymbial length, with several macrosetae and a dorsal process; paracymbium short, ca. 1/3 of cymbial length, hook shaped; pimoid cymbial sclerite V-shaped, ca. 1/3 of cymbial length; cymbial denticulate process short and distally curved, with more than 15 cuspules; median apophysis slender; conductor indistinct; pimoid embolic process distally pointed, shorter than embolus; embolus beginning at the 4:30 o’clock position; embolic tooth absent.

**Female (*paratype*)**: Total length 8.80. Carapace 3.44 long, 3.22 wide. Abdomen 5.36 long, 3.36 wide. Eye sizes and interdistances: AME 0.19, ALE 0.19, PME 0.20, PLE 0.18; AME-AME 0.14, AME-ALE 0.18, PME-PME 0.17, PME-PLE 0.25. Leg measurements: I: 26.73 (7.25, 9.15, 7.55, 2.78); II: 23.40 (6.49, 7.72, 6.78, 2.41); III: 17.01 (5.19, 5.44, 4.72, 1.66); IV: 21.14 (6.16, 7.07, 5.88, 2.03). Habitus as in Fig. 42F, G. Carapace yellowish with black lateral margins; thoracic fovea and radial grooves distinct; sternum brownish. Abdomen dark brown with yellowish transverse chevrons. Legs brownish with black annulations. Epigyne (Fig. 42A–D): triangular; ventral plate broad, length subequal to width; dorsal plate triangular; copulatory openings distinct; spermathecae nearly rectangular, divided into two parts, separated by ca. 1/2 width of spermatheca; fertilization ducts yellowish, laterally oriented.

##### Distribution.

Known only from the type locality, Tibet, China (Fig. 59).

#### Pimoa shigatse

Taxon classificationAnimaliaAraneaePimoidae

Zhang & Li sp. nov.

74961D62-3917-52CE-9BAB-669D54054747

http://zoobank.org/A3942B8F-2572-49DB-A2BF-F918652BCB17

Figures 43 , 59

##### Type material.

***Holotype*:** ♀ (IZCAS-Ar42001), China, Tibet, Shigatse, the 1220 km marker on the way of Yadong County to Kambu Township, 27.59°N, 88.91°E, ca. 3401 m, 11.X.2020, Z. Chen leg. ***Paratype***: 1♀ (IZCAS-Ar42002), same data as holotype.

##### Etymology.

The specific name is a noun in apposition taken from the type locality.

##### Diagnosis.

**Figure 43.**
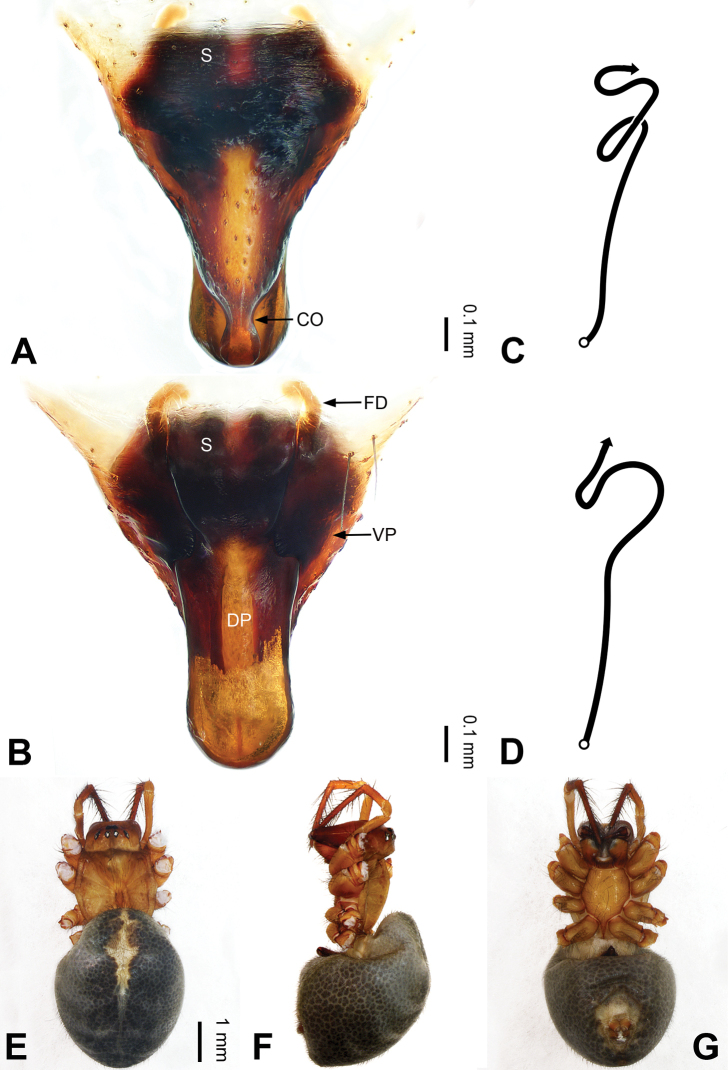
Epigyne and habitus of *Pimoa shigatse* sp. nov., female holotype **A** epigyne, ventral view **B** schematic course of internal duct system, ventral view **C** vulva, dorsal view **D** schematic course of internal duct system, dorsal view **E** female habitus, dorsal view **F** female habitus, lateral view **G** female habitus, ventral view. Abbreviations: CO = copulatory opening; DP = dorsal plate of the epigyne; FD = fertilization duct; S = spermatheca; VP = ventral plate of epigyne. Scale bars: equal for **E–G**.

*Pimoa shigatse* sp. nov. resembles those of *P. nyingchi* (see Zhang et al. 2020: 91, fig. 9A–D) and *P. reniformis* (see Xu and Li 2007: 493, figs 42–47) but can be distinguished by the distally blunt dorsal plate (Fig. 43B) (vs. distally pointed in *P. nyingchi* and distally narrow in *P. reniformis*) and also from *P. reniformis* by the small separation of the spermathecae (Fig. 43A) (vs. unseparated).

##### Description.

**Female (*holotype*)**: Total length 9.52. Carapace 3.08 long, 3.36 wide. Abdomen 6.44 long, 5.52 wide. Eye sizes and interdistances: AME 0.20, ALE 0.18, PME 0.16, PLE 0.18; AME-AME 0.09, AME-ALE 0.33, PME-PME 0.19, PME-PLE 0.32. Leg measurements: I: 37.06 (10.28, 12.34, 10.56, 3.88); II: 34.65 (9.64, 11.24, 10.36, 3.41); III: 23.91 (7.24, 6.59, 7.80, 2.28); IV: 32.07 (9.02, 10.56, 9.81, 2.68). Habitus as in Fig. 43E–G. Carapace yellowish with brown lateral margins; thoracic fovea and radial grooves distinct; sternum brownish. Abdomen gray with brownish transverse bands and a yellowish vertical band not extending to distal part. Legs brownish without annulations. Epigyne (Fig. 43A–D): subtriangular; ventral plate broad, length subequal to width; dorsal plates tongue shaped, distally blunt, width ca. 1/2 to length; copulatory openings distinct; spermathecae bean-shaped, touching each other; fertilization ducts yellow, laterally oriented.

**Male**: Unknown.

##### Distribution.

Known only from the type locality, Tibet, China (Fig. 59).

#### Pimoa tengchong

Taxon classificationAnimaliaAraneaePimoidae

Zhang & Li sp. nov.

F87BC9D1-E803-5862-B18D-785DD98F7531

http://zoobank.org/E59264E1-F0E6-4792-AE36-BE5A5EEB1083

**Figure 44.**
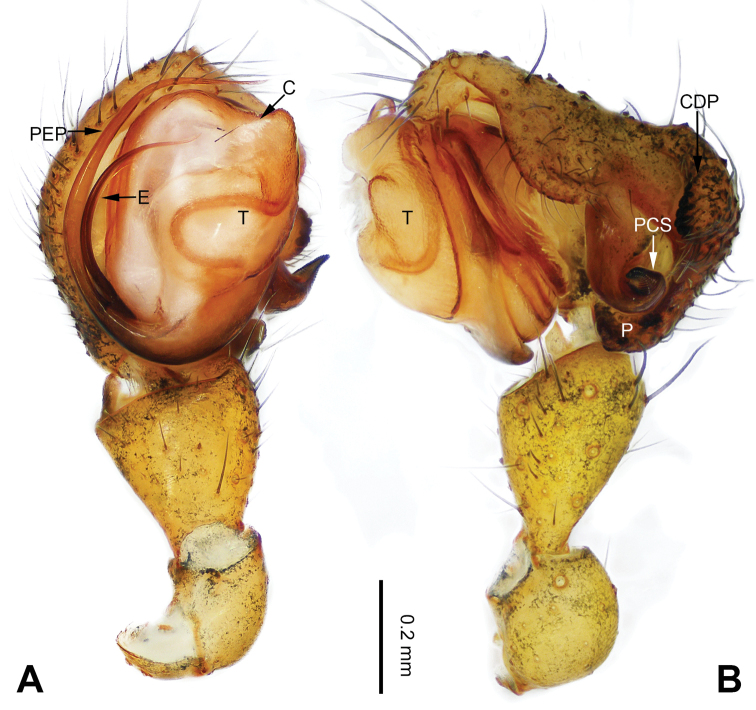
Left palp of *Pimoa tengchong* sp. nov., holotype **A** prolateral view **B** retrolateral view. Abbreviations: C = conductor; CDP = cymbial denticulate process; E = embolus; P = paracymbium; PCS = pimoid cymbial sclerite; PEP = pimoid embolic process; T = tegulum. Scale bar: equal for **A, B**.

**Figure 45.**
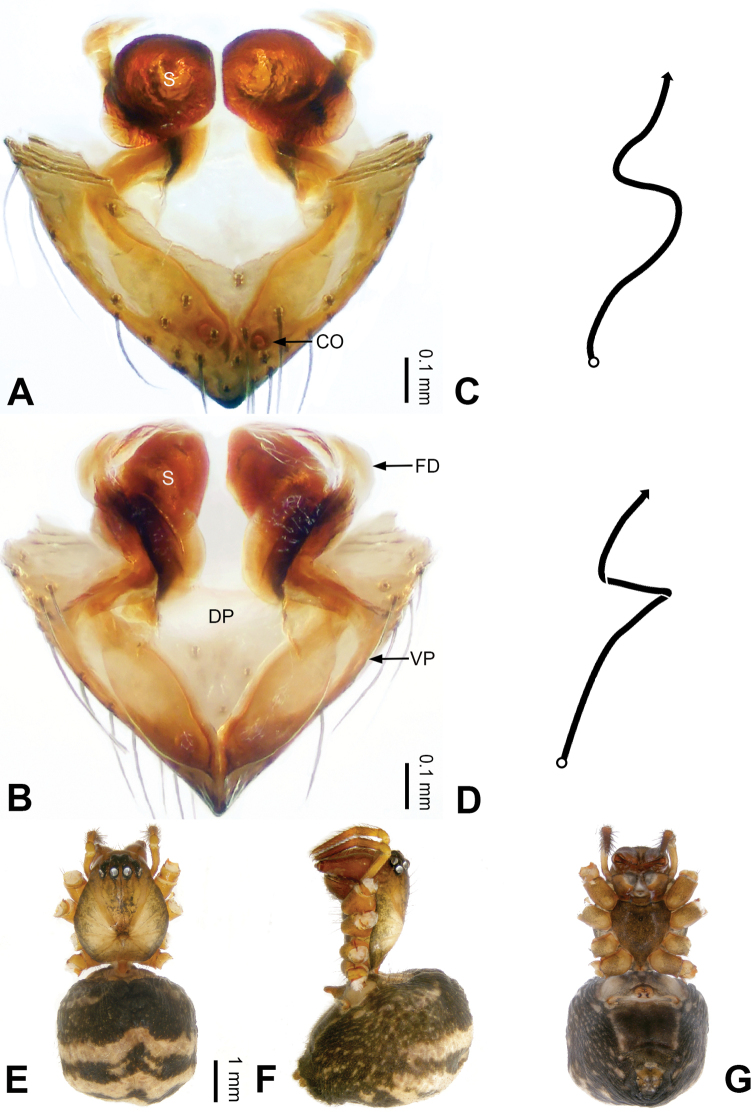
Epigyne and habitus of *Pimoa tengchong* sp. nov., female paratype and male holotype **A** epigyne, ventral view **B** vulva, dorsal view **C** schematic course of internal duct system, ventral view **D** schematic course of internal duct system, dorsal view **E** male habitus, dorsal view **F** female habitus, dorsal view **G** female habitus, ventral view. Abbreviations: CO = copulatory opening; DP = dorsal plate of the epigyne; FD = fertilization duct; S = spermatheca; VP = ventral plate of epigyne. Scale bars: equal for **E, G**.

Figures 44 , 45 , 57 , 59

##### Type material.

***Holotype*:** ♂ (IZCAS-Ar42003), China, Yunnan, Tengchong, Xincheng District, Luoshui Cave, 25.34°N, 98.54°E, ca. 1937 m, 15.VII.2016, Y. Li leg. ***Paratypes***: 2♀ (IZCAS-Ar42004-Ar42005), same data as holotype.

##### Etymology.

The specific name is a noun in apposition taken from the type locality.

##### Diagnosis.

The male of *Pimoa tengchong* sp. nov. resembles those of *P. binchuanensis* (see Zhang and Li 2019: 3, fig. 1A–C) and *P. duiba* (see Zhang et al. 2020: 84, fig. 3A–C) but can be distinguished from *P. binchuanensis* by the short, broad cymbial denticulate process (Fig. 57C) (vs. broad and long) and by the distally pointed pimoid embolic process (Fig. 57C) (vs. tip with fine granulations) and distinguished from *P. duiba* by the embolus without a spine (Fig. 57C) (vs. with a short, slender spine proximally) and by the distally curved pimoid cymbial sclerite (Fig. 57C) (vs. nearly V-shaped). The female of *P. tengchong* sp. nov. resembles *P. duiba* (see Zhang et al. 2020: 84, fig. 4A–D) but can be distinguished by the triangular dorsal plate (Fig. 45B) (vs. tongue-shaped) and by the medially located fertilization ducts separated by ca. 1/2 width of the dorsal plate (Fig. 45B) (vs. smaller separation).

##### Description.

**Male (*holotype*)**: Total length –. Carapace missing. Abdomen 2.31 long, 1.22 wide. Abdomen black with yellow transverse chevrons, nearly oval. Legs missing. Palp (Figs 44A, B, 57C): patella short, ca. 1/2 of tibial length; tibia short, ca. 1/2 of cymbial length, with several macrosetae and a dorsal process; paracymbium short, ca. 1/3 of cymbial length, hook shaped; pimoid cymbial sclerite L-shaped, distally curved, ca. 1/3 of cymbial length; cymbial denticulate process short and broad, with more than 11 cuspules; median apophysis slender; conductor distinct; pimoid embolic process distally pointed, longer than embolus; embolus beginning at the 7:30 o’clock position, suddenly narrowing distally; embolic tooth absent.

**Female (*paratype*)**: Total length 4.91. Carapace 2.28 long, 1.80 wide. Abdomen 2.63 long, 2.66 wide. Eye sizes and interdistances: AME 0.14, ALE 0.18, PME 0.16, PLE 0.15; AME-AME 0.11, AME-ALE 0.07, PME-PME 0.11, PME-PLE 0.12. Leg measurements: I: 11.62 (3.28, 4.31, 2.97, 1.06); II: 9.92 (2.97, 3.44, 2.56, 0.95); III: 7.05 (2.15, 2.24, 1.72, 0.94); IV: 9.68 (2.81, 3.23, 2.44, 1.20). Habitus as in Fig. 45E–G. Carapace yellowish with black lateral margins; thoracic fovea and radial grooves distinct; sternum brownish. Abdomen black with yellowish transverse bands. Legs yellowish with black annulations. Epigyne (Fig. 45A–D): triangular; ventral plate broad, width subequal to length; dorsal plate triangular, with a tip distally; copulatory openings distinct; spermathecae oval, with small separation; fertilization ducts membranous, laterally oriented.

##### Distribution.

Known only from the type locality, Yunnan, China (Fig. 59).

#### Pimoa xiahe

Taxon classificationAnimaliaAraneaePimoidae

Zhang & Li sp. nov.

19D3560D-2295-5E82-9E3D-72572F12FDA4

http://zoobank.org/E40972AB-9699-4C95-95F4-F8745B20F2CB

**Figure 46.**
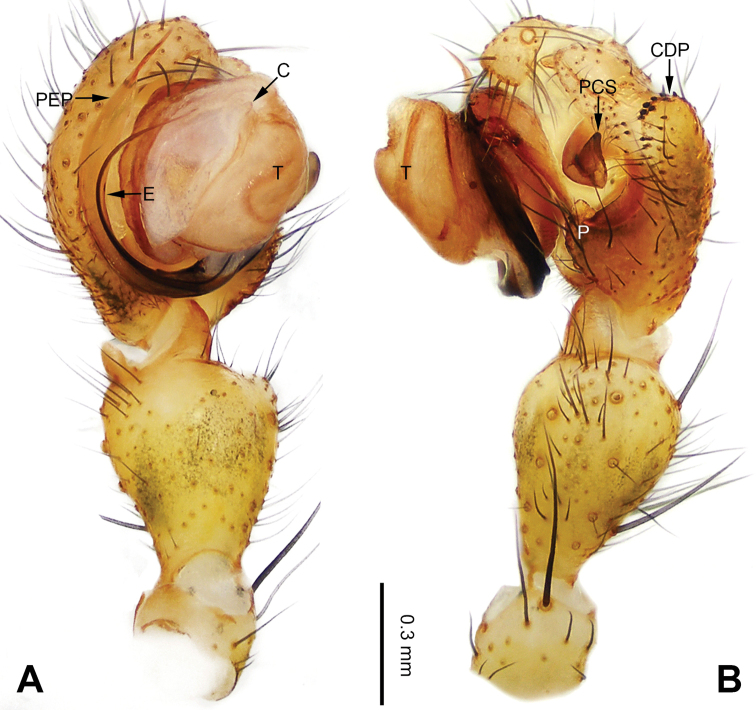
Left palp of *Pimoa xiahe* sp. nov., holotype **A** prolateral view **B** retrolateral view. Abbreviations: C = conductor; CDP = cymbial denticulate process; E = embolus; P = paracymbium; PCS = pimoid cymbial sclerite; PEP = pimoid embolic process; T = tegulum. Scale bar: equal for **A, B**.

**Figure 47.**
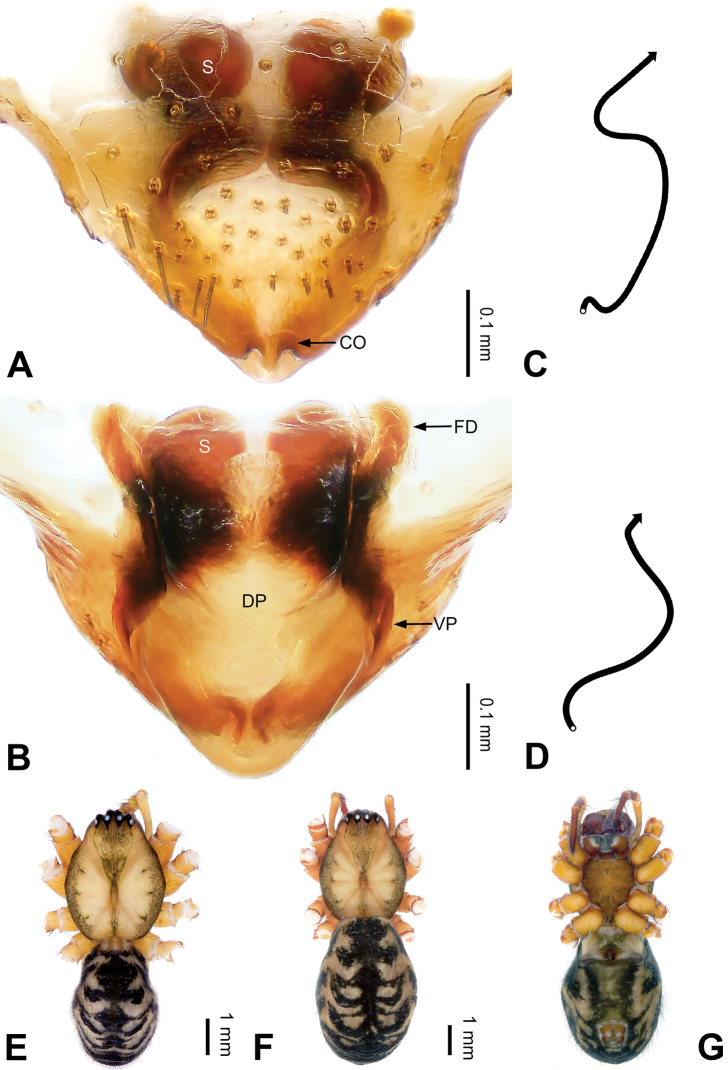
Epigyne and habitus of *Pimoa xiahe* sp. nov., female paratype and male holotype **A** epigyne, ventral view **B** vulva, dorsal view **C** schematic course of internal duct system, ventral view **D** schematic course of internal duct system, dorsal view **E** male habitus, dorsal view **F** female habitus, dorsal view **G** female habitus, ventral view. Abbreviations: CO = copulatory opening; DP = dorsal plate of the epigyne; FD = fertilization duct; S = spermatheca; VP = ventral plate of epigyne. Scale bars: equal for **F, G**.

Figures 46 , 47 , 57 , 59

##### Type material.

***Holotype*:** ♂ (IZCAS-Ar42006), China, Gansu, Gannan Tibetan Autonomous Prefecture, Xiahe County, Damai Township, Xiongmao Valley, 35.16°N, 102.67°E, ca. 3046 m, 10.VII.2020, Y. Lin and Z. Wang leg. ***Paratypes***: 1♂2♀ (IZCAS-Ar42007-Ar42009), same data as holotype.

##### Etymology.

The specific name is a noun in apposition taken from the type locality.

##### Diagnosis.

The male of *Pimoa xiahe* sp. nov. resembles those of *P. samyai* (see Zhang et al. 2020: 97, fig. 12) and *P. crispa* (see Hormiga 1994a: 63, figs 233–238; Hormiga 1994b: fig. 1A, B) but can be distinguished by the short cymbial denticulate process with few cuspules (Figs 46B, 57D) (vs. large, with many cuspules) and a proximal apophysis of the pimoid embolic process (Fig. 57D) (vs. without apophysis). The female of *P. xiahe* sp. nov. also resembles those of *P. crispa* (see Hormiga 1994a: 63, figs 239–247) and *P. samyai* (see Zhang et al. 2020: 97, fig. 13A–D) but can be distinguished by the medially wide dorsal plate (Fig. 47B) (vs. medially narrow).

##### Description.

**Male (*holotype*)**: Total length 6.84. Carapace 3.52 long, 2.78 wide. Abdomen 3.32 long, 2.63 wide. Eye sizes and interdistances: AME 0.13, ALE 0.18, PME 0.15, PLE 0.18; AME-AME 0.17, AME-ALE 0.19, PME-PME 0.19, PME-PLE 0.20. Leg measurements: I: 30.26 (8.25, 10.10, 8.94, 2.97); II: 28.61 (8.09, 9.52, 8.53, 2.47); III: 18.65 (5.59, 5.75, 5.37, 1.94); IV: 23.97 (7.03, 7.81, 6.75, 2.38). Habitus as in Fig. 47E. Carapace yellowish with black lateral margins; thoracic fovea and radial grooves distinct; sternum brownish. Abdomen black with yellowish transverse chevrons. Legs brownish with black annulations. Palp (Figs 46A, B, 57D): patella short, ca. 1/2 of tibial length, with one retrolateral macroseta; tibia short, subequal to cymbial length, with several macrosetae and a dorsal process; paracymbium short, ca. 1/3 of cymbial length, hook shaped; pimoid cymbial sclerite L-shaped, distally with scales, ca. 1/3 of cymbial length; cymbial denticulate process short, with more than 7 cuspules; median apophysis indistinct; conductor distinct and membranous; pimoid embolic process broad, suddenly narrowing distally, with proximal apophysis, longer than embolus; embolus beginning at the 5:30 o’clock position; embolic tooth absent.

**Female (*paratype*)**: Total length 8.30. Carapace 3.68 long, 2.89 wide. Abdomen 4.62 long, 2.87 wide. Eye sizes and interdistances: AME 0.21, ALE 0.20, PME 0.18, PLE 0.19; AME-AME 0.13, AME-ALE 0.14, PME-PME 0.16, PME-PLE 0.20. Leg measurements: I: 23.38 (6.63, 7.96, 6.16, 2.63); II: 21.06 (6.11, 7.07, 5.63, 2.25); III: 14.61 (4.22, 4.67, 4.13, 1.59); IV: 19.22 (5.91, 6.12, 5.28, 1.91). Habitus as in Fig. 47F, G. Carapace yellowish with black lateral margins; thoracic fovea and radial grooves distinct; sternum brownish. Abdomen black with yellowish transverse chevrons. Legs yellowish with black annulations. Epigyne (Fig. 47A–D): triangular; ventral plate broad, width subequal to length; dorsal plate tongue shaped; copulatory openings distinct; spermathecae nearly oval, separated by ca. 1/3 width of spermatheca; fertilization ducts yellowish, laterally oriented.

##### Distribution.

Known only from the type locality, Gansu, China (Fig. 59).

#### Pimoa yejiei

Taxon classificationAnimaliaAraneaePimoidae

Zhang & Li sp. nov.

578EDFF2-E63F-5AD6-9D56-29884932BED6

http://zoobank.org/0B6E2170-5A3A-4ECD-ADCB-C47887CD4EA1

Figures 48 , 59

##### Type material.

***Holotype*:** ♀ (IZCAS-Ar42010), China, Shaanxi, Paoki, Mei County, Taibaishan Nation Forest Park, 34.02°N, 107.87°E, ca. 1656 m, 20.VII.2020, Y. Lin and Z. Wang leg. ***Paratype***: 1♀ (IZCAS-Ar42011), same data as holotype.

##### Etymology.

The specific name is named after the collector Yejie Lin and is a noun (name) in genitive case.

##### Diagnosis.

**Figure 48.**
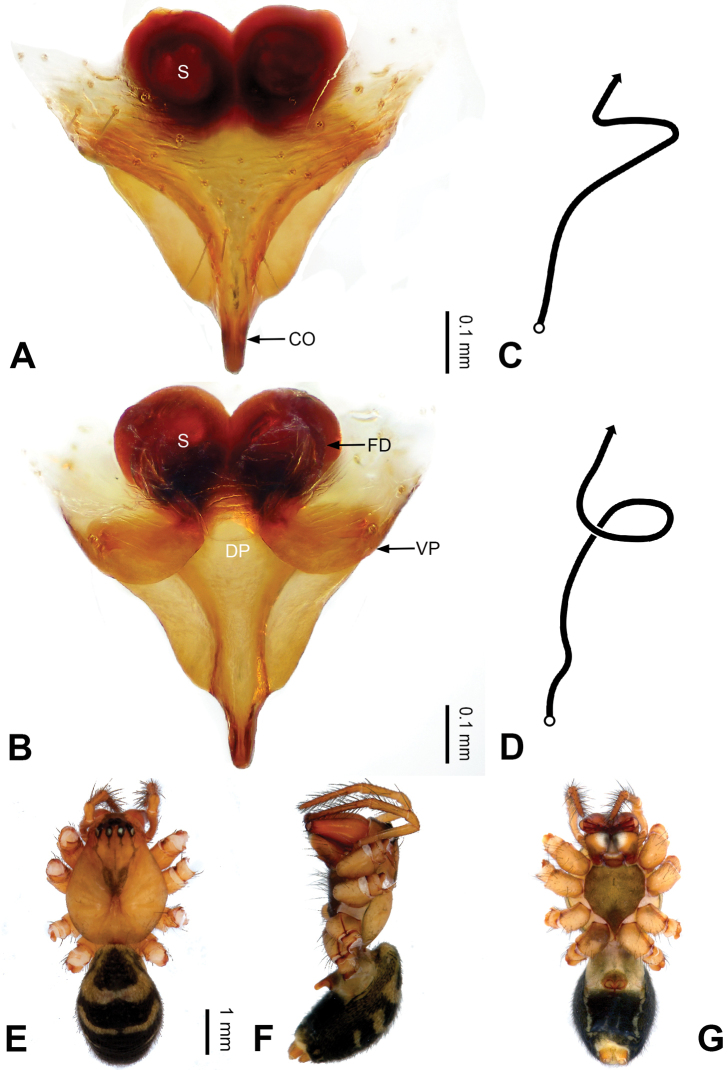
Epigyne and habitus of *Pimoa yejiei* sp. nov., female holotype **A** epigyne, ventral view **B** schematic course of internal duct system, ventral view **C** vulva, dorsal view **D** schematic course of internal duct system, dorsal view **E** female habitus, dorsal view **F** female habitus, lateral view **G** female habitus, ventral view. Abbreviations: CO = copulatory opening; DP = dorsal plate of the epigyne; FD = fertilization duct; S = spermatheca; VP = ventral plate of epigyne. Scale bars: equal for **E–G**.

*Pimoa yejiei* sp. nov. resembles *P. mainling* (see Zhang et al. 2020: 89, fig. 7A–D), but can be distinguished by the broad and round dorsal plates (Fig. 48A–D) (vs. narrow).

##### Description.

**Female (*holotype*)**: Total length 5.62. Carapace 2.84 long, 1.28 wide. Abdomen 2.78 long, 1.94 wide. Eye sizes and interdistances: AME 0.11, ALE 0.16, PME 0.17, PLE 0.18; AME-AME 0.04, AME-ALE 0.12, PME-PME 0.14, PME-PLE 0.13. Leg measurements: I: 19.42 (5.52, 6.50, 5.09, 2.31); II: 17.37 (4.84, 5.78, 4.81, 1.94); III: 13.91 (4.75, 4.25, 3.47, 1.44); IV: 16.10 (4.88, 5.25, 4.28, 1.69). Habitus as in Fig. 48E–G. Carapace yellowish with black lateral margins; thoracic fovea and radial grooves distinct; sternum brownish. Abdomen black with yellowish transverse bands and a vertical band not extending to distal part. Legs yellowish without annulations. Epigyne (Fig. 48A–D): funnel-shaped; ventral plates narrow, with a tip, length subequal to width; copulatory openings distinct; spermathecae round, unseparated; fertilization ducts hyaline, anteriorly oriented.

**Male**: Unknown.

##### Distribution.

Known only from the type locality, Shaanxi, China (Fig. 59).

#### Pimoa yele

Taxon classificationAnimaliaAraneaePimoidae

Zhang & Li sp. nov.

F1A6C2DA-677B-57BC-A6CA-EE2AFBF85C64

http://zoobank.org/933D7B0C-77EE-44AA-8892-7161A194C5E4

**Figure 49.**
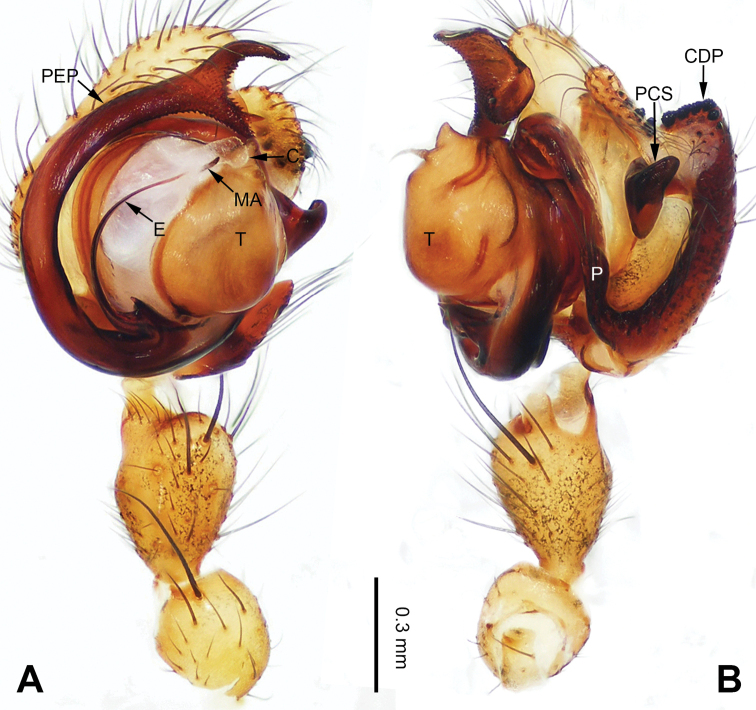
Left palp of *Pimoa yele* sp. nov., holotype **A** prolateral view **B** retrolateral view. Abbreviations: C = conductor; CDP = cymbial denticulate process; E = embolus; MA = median apophysis; P = paracymbium; PCS = pimoid cymbial sclerite; PEP = pimoid embolic process; T = tegulum. Scale bar: equal for **A, B**.

**Figure 50.**
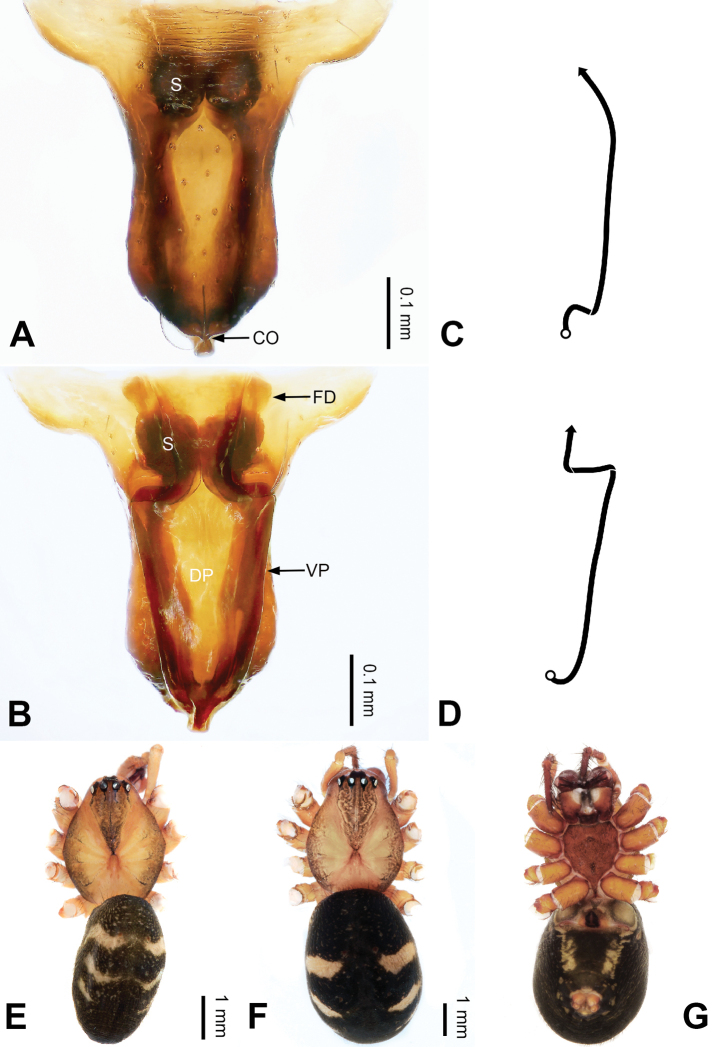
Epigyne and habitus of *Pimoa yele* sp. nov., female paratype and male holotype **A** epigyne, ventral view **B** vulva, dorsal view **C** schematic course of internal duct system, ventral view **D** schematic course of internal duct system, dorsal view **E** male habitus, dorsal view **F** female habitus, dorsal view **G** female habitus, ventral view. Abbreviations: CO = copulatory opening; DP = dorsal plate of the epigyne; FD = fertilization duct; S = spermatheca; VP = ventral plate of epigyne. Scale bars: equal for **F, G**.

Figures 49 , 50 , 58 , 59

##### Type material.

***Holotype*:** ♂ (IZCAS-Ar42012), China, Sichuan, Liangshan, Mianning County, Yele Town, the abandoned mine opposite the Yichang Family, 28.93°N, 102.23°E, ca. 2471 m, 13.III.2019, Z. Chen leg. ***Paratypes***: 1♂2♀ (IZCAS-Ar42013-Ar42015), same data as holotype.

##### Etymology.

The specific name is a noun in apposition taken from the type locality.

##### Diagnosis.

The male of *Pimoa yele* sp. nov. resembles those of *P. jinchuan* sp. nov. (Figs 19A, B, 55C), *P. lata* (see Zhang and Li 2019: 6, fig. 3A–C), and *P. trifurcata* (see Xu and Li 2007: 496, figs 48–54) but can be distinguished from *P. jinchuan* sp. nov. and *P. lata* by the embolus with a short, slender spine proximally (Fig. 58A) (vs. without a spine) and the short and distally narrow cymbial denticulate process (Fig. 58A) (vs. broad) and distinguished from *P. trifurcata* by the distally bifurcate apex of the pimoid embolic process (Fig. 1B) (vs. trifurcate apex). The female of *P. yele* sp. nov. resembles those of *P. crispa* (see Hormiga 1994a: 63, figs 239–247) and *P. jinchuan* sp. nov. (Fig. 20A–D) but can be distinguished by the bullet-shaped ventral plate (Fig. 50A) (vs. triangular in *P. crispa* and tongue shaped and distally curved in *P. jinchuan* sp. nov.) and also from *P. crispa* by the unseparated spermathecae (Fig. 50A) (vs. with small separation).

##### Description.

**Male (*holotype*)**: Total length 6.03. Carapace 2.56 long, 2.38 wide. Abdomen 3.47 long, 2.16 wide. Eye sizes and interdistances: AME 0.11, ALE 0.19, PME 0.17, PLE 0.18; AME-AME 0.09, AME-ALE 0.14, PME-PME 0.14, PME-PLE 0.14. Leg measurements: I: 29.76 (8.66, 9.19, 9.13, 2.78); II: 24.98 (6.69, 8.19, 7.63, 2.47); III: 15.32 (4.44, 4.66, 4.63, 1.59); IV: 20.89 (6.22, 6.82, 5.91, 1.94). Habitus as in Fig. 50E. Carapace yellowish with black lateral margins; thoracic fovea and radial grooves distinct; sternum brownish. Abdomen black with yellow transverse chevrons, nearly oval. Legs brownish without annulations. Palp (Figs 49A, B, 58A): patella short, almost as long as tibial length, with one retrolateral macroseta; tibia short, ca. 1/2 of cymbial length, with several macrosetae and a dorsal process; paracymbium short, ca. 1/3 of cymbial length, finger shaped; pimoid cymbial sclerite V-shaped, ca. 1/3 of cymbial length; cymbial denticulate process short and distally narrow, with more than 12 cuspules; median apophysis slender; conductor distinct; pimoid embolic process broad, robust, with distally bifurcate apex with scales, longer than embolus; embolus beginning at the 8:00 o’clock position, with a short, slender spine proximally; embolic tooth absent.

**Female (*paratype*)**: Total length 8.62. Carapace 3.56 long, 3.03 wide. Abdomen 5.06 long, 3.09 wide. Eye sizes and interdistances: AME 0.19, ALE 0.20, PME 0.19, PLE 0.20; AME-AME 0.06, AME-ALE 0.21, PME-PME 0.21, PME-PLE 0.22. Leg measurements: I: 26.85 (7.69, 9.00, 7.53, 2.63); II: 23.07 (6.56, 7.72, 6.63, 2.16); III: 17.04 (5.44, 5.13, 4.72, 1.75); IV: 21.93 (6.78, 7.12, 6.09, 1.94). Habitus as in Fig. 50F, G. Carapace yellowish with black lateral margins; thoracic fovea and radial grooves distinct; sternum brownish. Abdomen black with yellowish transverse bands. Legs brownish without annulations. Epigyne (Fig. 50A–D): bullet-shaped; ventral plate broad, width ca. 1/2 of length; dorsal plate triangular, with a distal tip; copulatory openings distinct; spermathecae oval, unseparated; fertilization ducts yellowish, laterally oriented.

##### Distribution.

Known only from the type locality, Sichuan, China (Fig. 59).

#### Pimoa zayu

Taxon classificationAnimaliaAraneaePimoidae

Zhang & Li sp. nov.

39A1268F-E083-5E32-8FC9-B430E4B8FA74

http://zoobank.org/F2908187-468A-4DB3-9D50-370695DC16AD

**Figure 51.**
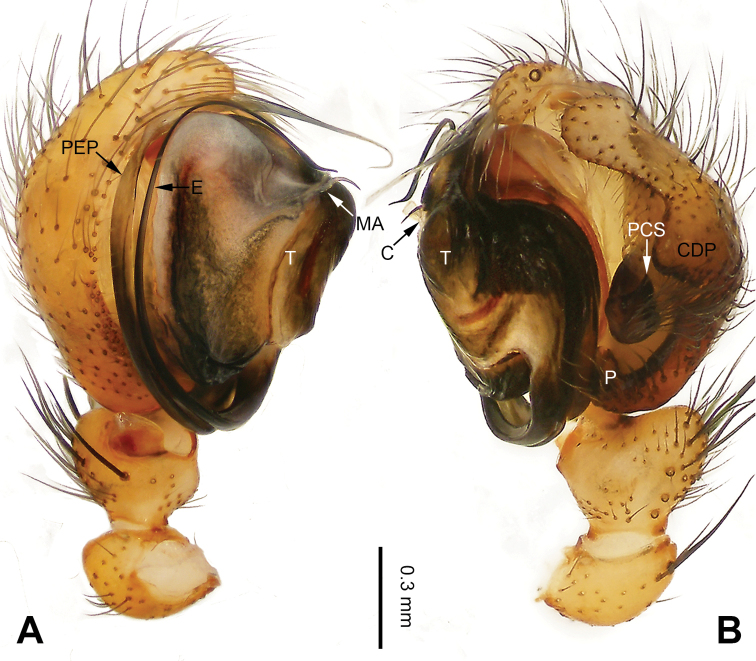
Left palp of *Pimoa zayu* sp. nov., holotype **A** prolateral view **B** retrolateral view. Abbreviations: C = conductor; CDP = cymbial denticulate process; E = embolus; MA = median apophysis; P = paracymbium; PCS = pimoid cymbial sclerite; PEP = pimoid embolic process; T = tegulum. Scale bar: equal for **A, B**.

**Figure 52.**
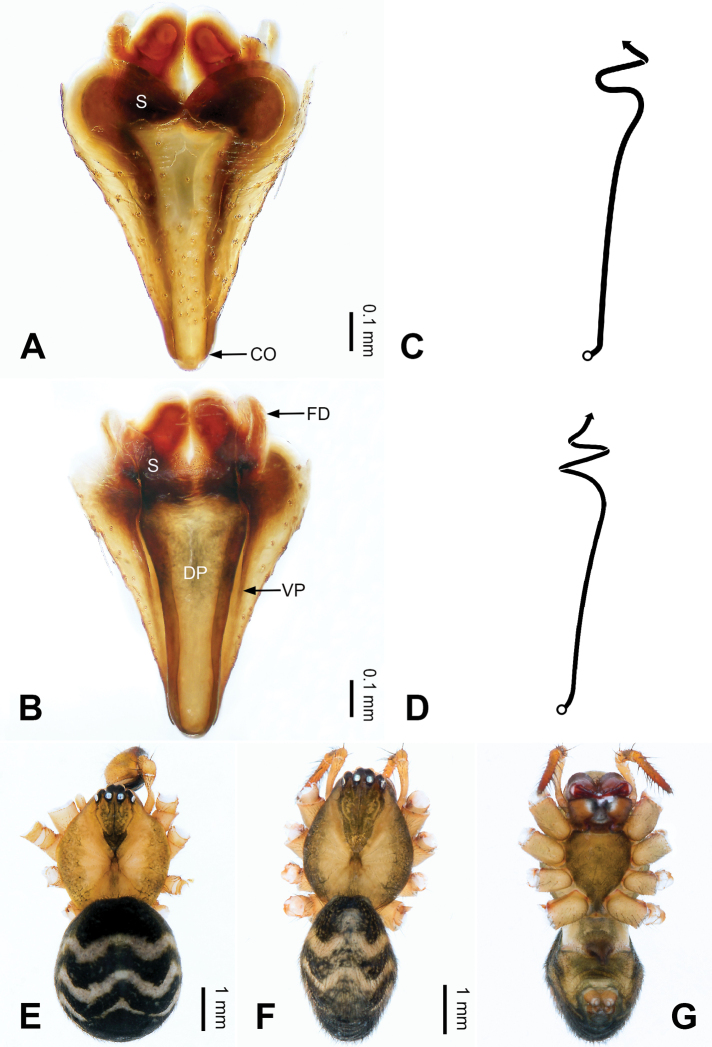
Epigyne and habitus of *Pimoa zayu* sp. nov., female paratype and male holotype **A** epigyne, ventral view **B** vulva, dorsal view **C** schematic course of internal duct system, ventral view **D** schematic course of internal duct system, dorsal view **E** male habitus, dorsal view **F** female habitus, dorsal view **G** female habitus, ventral view. Abbreviations: CO = copulatory opening; DP = dorsal plate of the epigyne; FD = fertilization duct; S = spermatheca; VP = ventral plate of epigyne. Scale bars: equal for **F, G**.

Figures 51 , 52 , 58 , 59

##### Type material.

***Holotype*:** ♂ (IZCAS-Ar2016), China, Tibet, Nyingchi, Zayu County, Ridong Village, 28.52°N, 98.08°E, ca. 3572 m, 30.VII.2019, X. Zhang, Z. Bai and J. Liu leg. ***Paratypes***: 1♂2♀ (IZCAS-Ar42017-Ar42019), same data as holotype.

##### Etymology.

The specific name is a noun in apposition taken from the type locality.

##### Diagnosis.

The male of *Pimoa zayu* sp. nov. resembles those of *P. gandhii* (see Hormiga 1994a: 73, figs 218–223) and *P. nematoides* (see Hormiga 1994a: 71, figs 285–289) but can be distinguished by the cymbial denticulate process with many cuspules (Figs 51B, 58B) (vs. few cuspules), from *P. gandhii* by the longer, membranous pimoid embolic process (Fig. 58B) (vs. almost as long as embolus), and from *P. nematoides* by the broad cymbial denticulate process (Fig. 58B) (vs. narrow). The female of *P. zayu* sp. nov. also resembles *P. gandhii* (see Hormiga 1994a: 73, figs 224–231) but can be distinguished by the unseparated pair of nearly rectangular spermathecae (Fig. 52A) (vs. oval, separated by ca. 1/3 width of a spermatheca).

##### Description.

**Male (*holotype*)**: Total length 6.10. Carapace 2.63 long, 2.66 wide. Abdomen 3.47 long, 3.13 wide. Eye sizes and interdistances: AME 0.16, ALE 0.15, PME 0.17, PLE 0.17; AME-AME 0.19, AME-ALE 0.07, PME-PME 0.12, PME-PLE 0.16. Leg measurements: I: 23.27 (6.13, 7.81, 7.05, 2.28); II: 20.03 (5.69, 6.50, 5.78, 2.06); III: 13.63 (3.91, 4.41, 3.78, 1.53); IV: 16.19 (4.34, 5.41, 4.72, 1.72). Habitus as in Fig. 51E. Carapace yellowish with black lateral margins; thoracic fovea and radial grooves distinct; sternum brownish. Abdomen black with yellowish transverse chevrons. Legs yellowish with black annulations. Palp (Figs 51A, B, 58B): patella short, almost as long as tibial length, with one retrolateral macroseta; tibia short, ca. 1/3 of cymbial length, with several macrosetae and a dorsal process; paracymbium short, ca. 1/4 of cymbial length, finger shaped; pimoid cymbial sclerite U-shaped, ca. 1/4 of cymbial length; cymbial denticulate process short and broad, with more than 23 cuspules; median apophysis slender, membranous; conductor indistinct; pimoid embolic process membranous, longer than embolus; embolus beginning at the 4:00 o’clock position; embolic tooth absent.

**Female (*paratype*)**: Total length 5.49. Carapace 2.61 long, 2.25 wide. Abdomen 2.88 long, 2.28 wide. Eye sizes and interdistances: AME 0.18, ALE 0.19, PME 0.18, PLE 0.19 AME-AME 0.11, AME-ALE 0.10, PME-PME 0.12, PME-PLE 0.16. Leg measurements: I: 18.68 (5.31, 6.22, 4.84, 2.31); II: 15.59 (4.31, 5.31, 4.03, 1.94); III: 11.12 (3.31, 3.63, 2.84, 1.34); IV: 14.44 (4.53, 4.60, 3.78, 1.53). Habitus as in Fig. 52F, G. Carapace yellowish with black lateral margins; thoracic fovea and radial grooves distinct; sternum brownish. Abdomen grayish with yellowish transverse chevrons. Legs yellowish with black annulations. Epigyne (Fig. 52A–D): triangular; ventral plate broad, width ca. 1/2 of length; dorsal plate triangular; copulatory openings distinct; spermathecae nearly oval, unseparated; fertilization ducts yellowish, laterally oriented.

##### Distribution.

Known only from the type locality, Tibet, China (Fig. 59).

#### Pimoa zhigangi

Taxon classificationAnimaliaAraneaePimoidae

Zhang & Li sp. nov.

32F1DCCD-9AFF-5FA5-A426-7F57B1EAC1CD

http://zoobank.org/56CA5D2E-D5CC-47D9-8623-C1BE90B27623

Figures 53 , 59

##### Type material.

***Holotype*:** ♀ (IZCAS-Ar42020), China, Tibet, Nyingchi, Bayi District, Guncang Monba Ethnic Township, Guncang Village, 29.80°N, 94.10°E, ca. 3556 m, 1.X.2020, Z. Chen leg. ***Paratype***: 1♀ (IZCAS-Ar42021), same data as holotype.

##### Etymology.

The specific name is named after the collector Zhigang Chen and is a noun (name) in genitive case.

##### Diagnosis.

**Figure 53.**
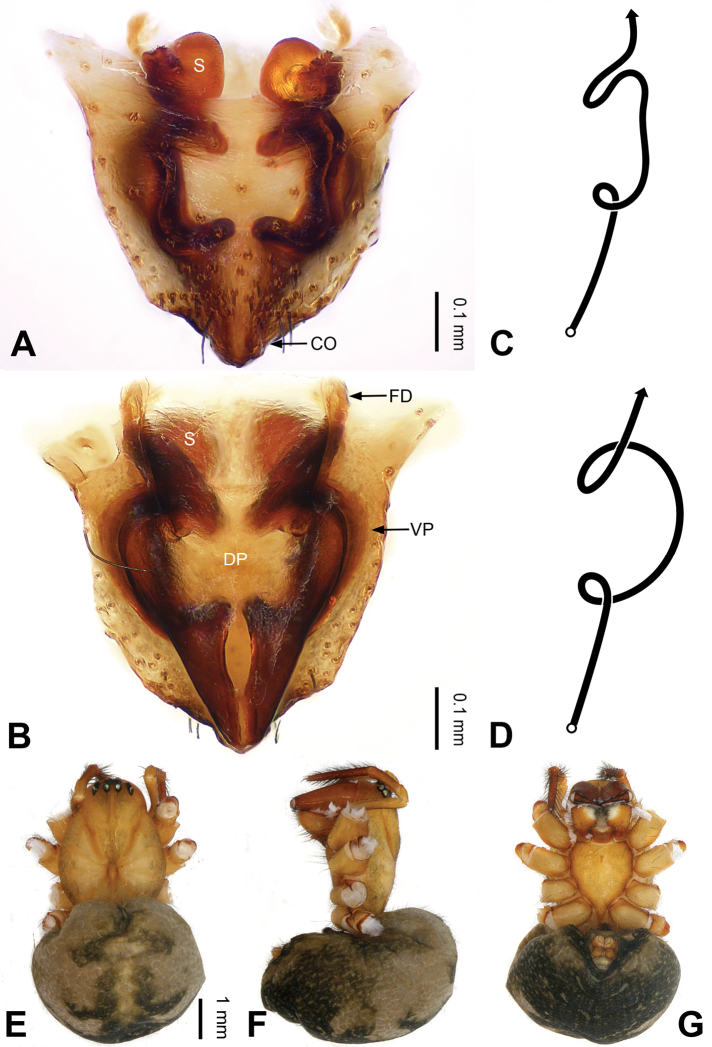
Epigyne and habitus of *Pimoa zhigangi* sp. nov., female holotype **A** epigyne, ventral view **B** schematic course of internal duct system, ventral view **C** vulva, dorsal view **D** schematic course of internal duct system, dorsal view **E** female habitus, dorsal view **F** female habitus, lateral view **G** female habitus, ventral view. Abbreviations: CO = copulatory opening; DP = dorsal plate of the epigyne; FD = fertilization duct; S = spermatheca; VP = ventral plate of epigyne. Scale bars: equal for **E–G**.

*Pimoa zhigangi* sp. nov. resembles those of *P. nyingchi* (see Zhang et al. 2020: 91, fig. 9A–D) and *P. reniformis* (see Xu and Li 2007: 493, figs 42–47) but can be distinguished by the spermathecae which are separated by ca. 1/2 the width of a spermatheca (Fig. 53A) (vs. unseparated) and also from *P. nyingchi* by the distally narrow dorsal plate (Fig. 53B) (vs. distally pointed).

##### Description.

**Female (*holotype*)**: Total length 6.49. Carapace 3.24 long, 2.66 wide. Abdomen 3.25 long, 4.28 wide. Eye sizes and interdistances: AME 0.17, ALE 0.19, PME 0.15, PLE 0.19; AME-AME 0.10, AME-ALE 0.13, PME-PME 0.14, PME-PLE 0.19. Leg measurements: I: 25.70 (7.41, 8.69, 7.22, 2.38); II: 21.65 (6.44, 6.81, 6.09, 2.31); III: 15.75 (4.81, 4.94, 4.41, 1.59); IV: 20.62 (6.34, 6.75, 5.66, 1.87). Habitus as in Fig. 53E–G. Carapace yellowish; thoracic fovea and radial grooves distinct; sternum yellow. Abdomen grayish with brownish transverse bands and a yellowish vertical band not extending to distal part. Legs brownish without annulations. Epigyne (Fig. 53A–D): subtriangular; ventral plate broad, length subequal to width; dorsal plates triangular, width ca. 1/2 to length; copulatory openings distinct; spermathecae nearly round, separated by ca. 1/2 width of spermatheca; fertilization ducts yellow, laterally oriented.

**Male**: Unknown.

##### Distribution.

Known only from the type locality, Tibet, China (Fig. 59).

**Figure 54.**
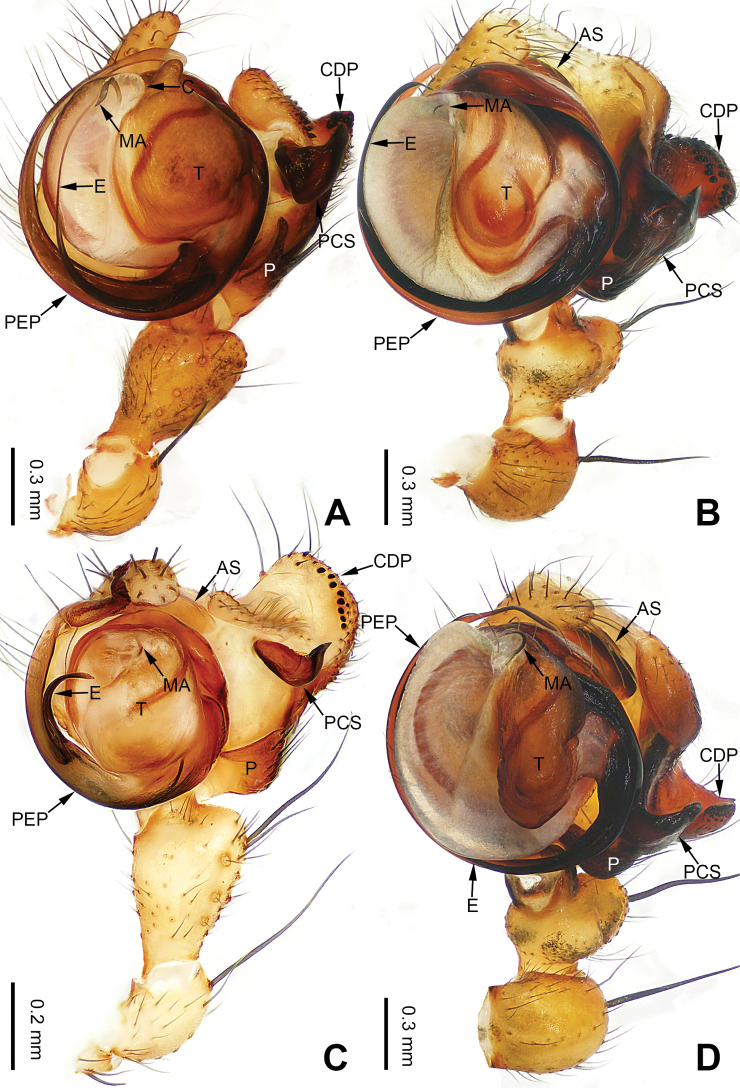
Left palps of *Pimoa* new species, ventral view, **A***Pimoa anning* sp. nov. **B***Pimoa bomi* sp. nov. **C***Pimoa dongjiu* sp. nov. **D***Pimoa gyara* sp. nov. Abbreviations: AS = alveolar sclerite; C = conductor; CDP = cymbial denticulate process; E = embolus; MA = median apophysis; P = paracymbium; PCS = pimoid cymbial sclerite; PEP = pimoid embolic process; T = tegulum.

**Figure 55.**
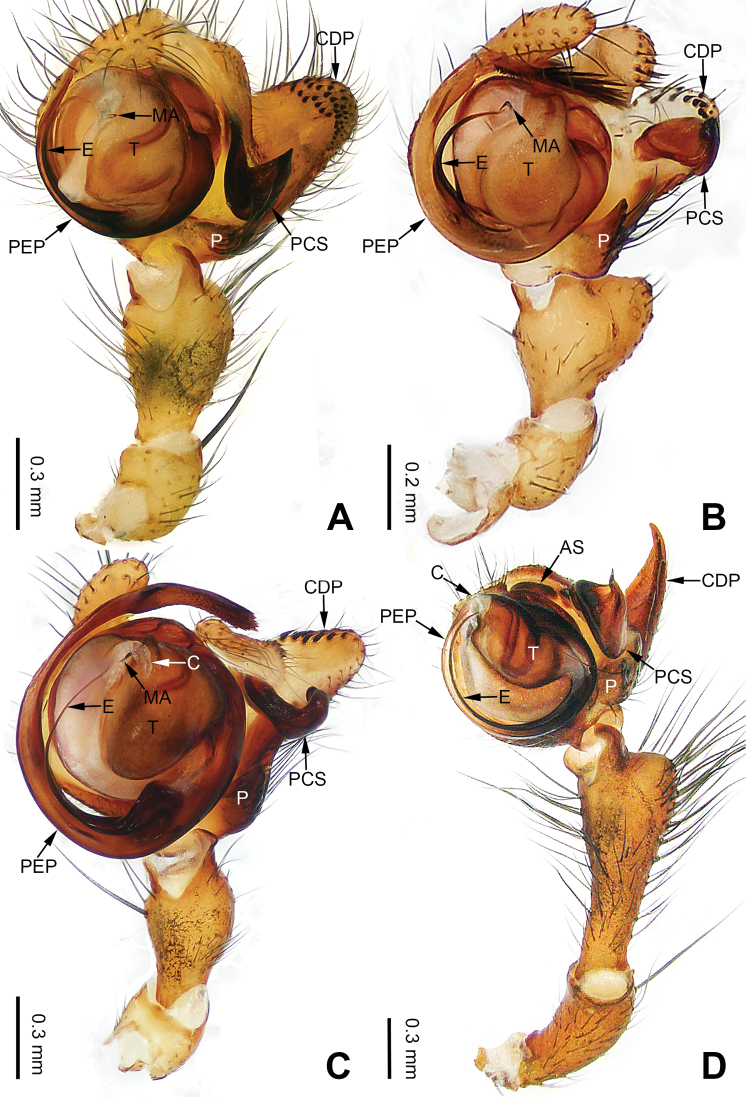
Left palps of *Pimoa* new species, ventral view, **A***Pimoa gyirong* sp. nov. **B***Pimoa heishui* sp. nov. **C***Pimoa jinchuan* sp. nov. **D***Pimoa mechi* sp. nov. Abbreviations: AS = alveolar sclerite; C = conductor; CDP = cymbial denticulate process; E = embolus; MA = median apophysis; P = paracymbium; PCS = pimoid cymbial sclerite; PEP = pimoid embolic process; T = tegulum.

**Figure 56.**
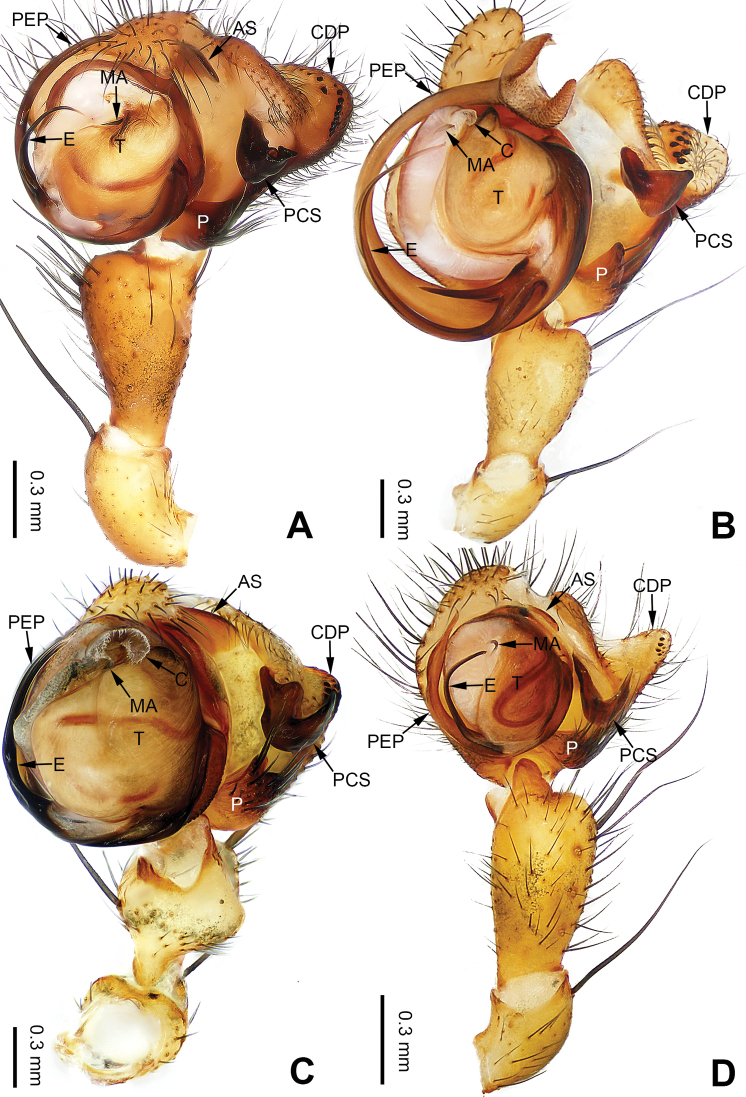
Left palps of *Pimoa* new species, ventral view **A***Pimoa miandam* sp. nov. **B***Pimoa miero* sp. nov. **C***Pimoa muli* sp. nov. **D***Pimoa nyalam* sp. nov. Abbreviations: AS = alveolar sclerite; C = conductor; CDP = cymbial denticulate process; E = embolus; MA = median apophysis; P = paracymbium; PCS = pimoid cymbial sclerite; PEP = pimoid embolic process; T = tegulum.

**Figure 57.**
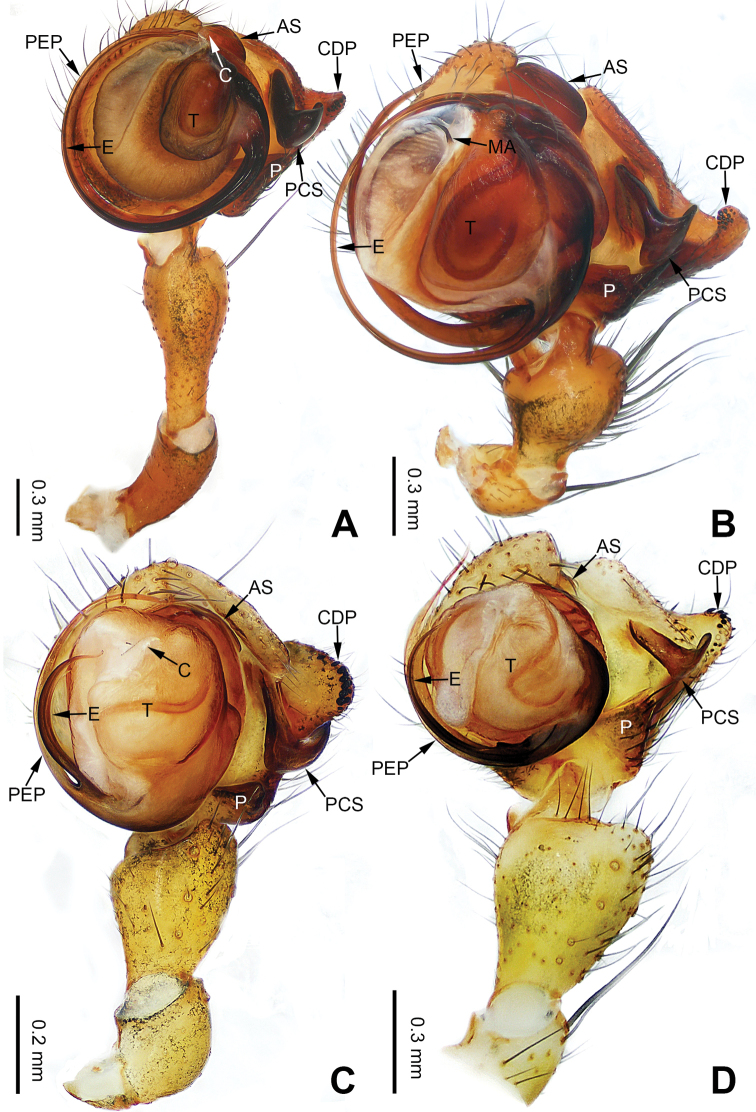
Left palps of *Pimoa* new species, ventral view **A***Pimoa phaplu* sp. nov. **B***Pimoa sangri* sp. nov. **C***Pimoa tengchong* sp. nov. **D***Pimoa xiahe* sp. nov. Abbreviations: AS = alveolar sclerite; C = conductor; CDP = cymbial denticulate process; E = embolus; MA = median apophysis; P = paracymbium; PCS = pimoid cymbial sclerite; PEP = pimoid embolic process; T = tegulum.

**Figure 58.**
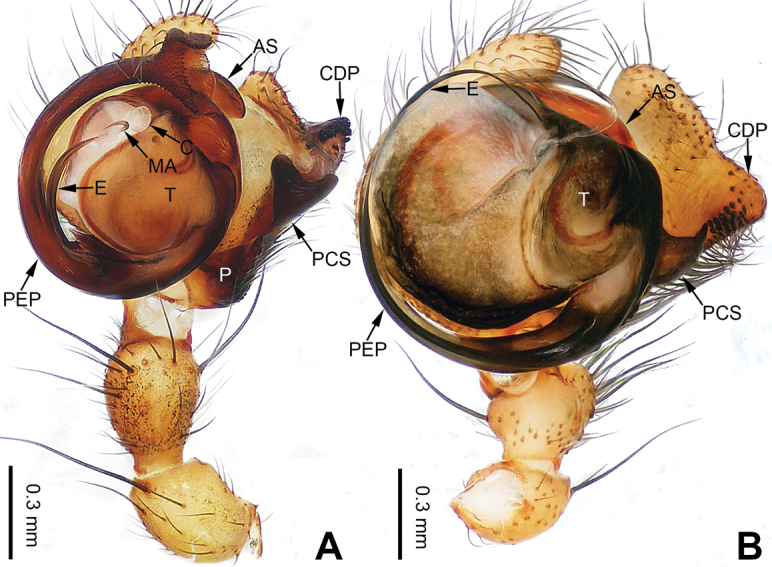
Left palps of *Pimoa* new species, ventral view **A***Pimoa yele* sp. nov. **B***Pimoa zayu* sp. nov. Abbreviations: AS = alveolar sclerite; C = conductor; CDP = cymbial denticulate process; E = embolus; MA = median apophysis; P = paracymbium; PCS = pimoid cymbial sclerite; PEP = pimoid embolic process; T = tegulum.

**Figure 59.**
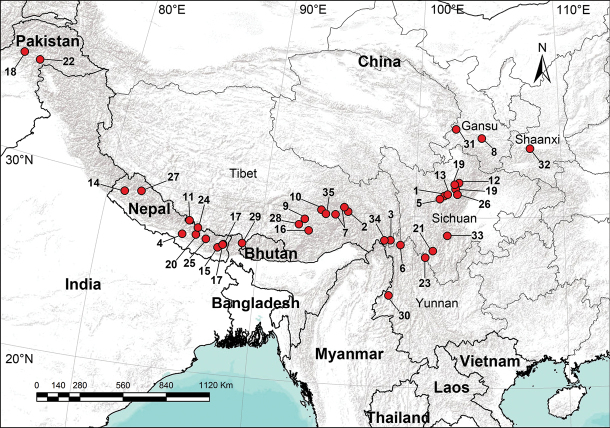
Distribution records of *Pimoa* new species in this paper **1***P. anning* sp. nov. **2***P. bomi* sp. nov. **3***P. cawarong* sp. nov. **4***P. daman* sp. nov. **5***P. danba* sp. nov. **6***P. deqen* sp. nov. **7***P. dongjiu* sp. nov. **8***P. guiqing* sp. nov. **9***P. gyaca* sp. nov. **10***P. gyara* sp. nov. **11***P. gyirong* sp. nov. **12***P. heishui* sp. nov. **13***P. jinchuan* sp. nov. **14***P. khaptad* sp. nov. **15***P. koshi* sp. nov. **16***P. lhatog* sp. nov. **17***P. mechi* sp. nov. **18***P. miandam* sp. nov. **19***P. miero* sp. nov. **20***P. mude* sp. nov. **21***P. muli* sp. nov. **22***P. naran* sp. nov. **23***P. ninglang* sp. nov. **24***P. nyalam* sp. nov. **25***P. phaplu* sp. nov. **26***P. putou* sp. nov. **27***P. rara* sp. nov. **28***P. sangri* sp. nov. **29***P. shigatse* sp. nov. **30***P. tengchong* sp. nov. **31***P. xiahe* sp. nov. **32***P. yejiei* sp. nov. **33***P. yele* sp. nov. **34***P. zayu* sp. nov. **35***P. zhigangi* sp. nov.

## Supplementary Material

XML Treatment for Pimoa

XML Treatment for Pimoa anning

XML Treatment for Pimoa bomi

XML Treatment for Pimoa cawarong

XML Treatment for Pimoa daman

XML Treatment for Pimoa danba

XML Treatment for Pimoa deqen

XML Treatment for Pimoa dongjiu

XML Treatment for Pimoa guiqing

XML Treatment for Pimoa gyaca

XML Treatment for Pimoa gyara

XML Treatment for Pimoa gyirong

XML Treatment for Pimoa heishui

XML Treatment for Pimoa jinchuan

XML Treatment for Pimoa khaptad

XML Treatment for Pimoa koshi

XML Treatment for Pimoa lhatog

XML Treatment for Pimoa mechi

XML Treatment for Pimoa miandam

XML Treatment for Pimoa miero

XML Treatment for Pimoa mude

XML Treatment for Pimoa muli

XML Treatment for Pimoa naran

XML Treatment for Pimoa ninglang

XML Treatment for Pimoa nyalam

XML Treatment for Pimoa phaplu

XML Treatment for Pimoa putou

XML Treatment for Pimoa rara

XML Treatment for Pimoa sangri

XML Treatment for Pimoa shigatse

XML Treatment for Pimoa tengchong

XML Treatment for Pimoa xiahe

XML Treatment for Pimoa yejiei

XML Treatment for Pimoa yele

XML Treatment for Pimoa zayu

XML Treatment for Pimoa zhigangi
